# Phase II Trial of Sorafenib in Combination with Carboplatin and Paclitaxel in Patients with Metastatic Uveal Melanoma: SWOG S0512

**DOI:** 10.1371/journal.pone.0048787

**Published:** 2012-11-30

**Authors:** Shailender Bhatia, James Moon, Kim A. Margolin, Jeffrey S. Weber, Christopher D. Lao, Megan Othus, Ana M. Aparicio, Antoni Ribas, Vernon K. Sondak

**Affiliations:** 1 Medical Oncology, University of Washington, Seattle, Washington, United States of America; 2 Biostatistics, SWOG Statistical Center, Seattle, Washington, United States of America; 3 Cutaneous Oncology, H. Lee Moffitt Cancer Center, Tampa, Florida, United States of America; 4 Medical Oncology, University of Michigan, Ann Arbor, Michigan, United States of America; 5 Medical Oncology, MD Anderson Cancer Center/University of Texas, Houston, Texas, United States of America; 6 Medical Oncology, University of California Los Angeles Medical Center, Los Angeles, California, United States of America; Davidoff Center, Israel

## Abstract

**Background:**

Sorafenib, a multikinase inhibitor of cell proliferation and angiogenesis, inhibits the mitogen-activated protein kinase pathway that is activated in most uveal melanoma tumors. This phase II study was conducted by the SWOG cooperative group to evaluate the efficacy of sorafenib in combination with carboplatin and paclitaxel (CP) in metastatic uveal melanoma.

**Methods:**

Twenty-five patients with stage IV uveal melanoma who had received 0–1 prior systemic therapy were enrolled. Treatment included up to 6 cycles of carboplatin (AUC = 6) and paclitaxel (225 mg/m^2^) administered IV on day 1 plus sorafenib (400 mg PO twice daily), followed by sorafenib monotherapy until disease progression. The primary endpoint was objective response rate (ORR); a two-stage design was used with the study to be terminated if no confirmed responses were observed in the first 20 evaluable patients. Secondary efficacy endpoints included progression-free survival (PFS) and overall survival (OS).

**Results:**

No confirmed objective responses occurred among the 24 evaluable patients (ORR = 0% [95% CI: 0–14%]) and the study was terminated at the first stage. Minor responses (tumor regression less than 30%) were seen in eleven of 24 (45%) patients. The median PFS was 4 months [95% CI: 1–6 months] and the 6-month PFS was 29% [95% CI: 13%–48%]. The median OS was 11 months [95% CI: 7–14 months].

**Conclusion:**

In this study, the overall efficacy of CP plus sorafenib in metastatic uveal melanoma did not warrant further clinical testing when assessed by ORR, although minor tumor responses and stable disease were observed in some patients.

**Trial Registration:**

ClinicalTrials.gov NCT00329641

## Introduction

Uveal melanoma, arising from melanocytes within the uveal tract (the iris, ciliary body, and choroid of the eye), represents 3–5% of all melanomas with an age-adjusted incidence of 5.1 per million [1], [2], [3]. Despite the advances in treatment of patients with clinically localized disease, the 5-year relative survival rate (∼80%) of uveal melanoma has remained stable in the United States from 1973 to 2008 [3] and disease recurrence is common, with relapses sometimes seen decades after the initial presentation [4], [5]. Fatal metastatic disease eventually develops in up to 50% of patients with uveal melanoma [4], [5].

The development of metastatic disease was associated with a median survival of 3.6 months in the largest reported series of unselected cases [5]. Liver is usually the predominant site of metastases, and hepatic involvement has been associated with poor prognosis [6], [7]. Loco-regional therapies to the liver like radiofrequency ablation, cryoablation, chemoembolization or isolated hepatic perfusion therapy may delay the progression of hepatic metastases and may lead to palliation of cancer symptoms in selected patients [8]. The administration of these complex therapies is mostly restricted to ‘surgically-fit’ patients at major academic centers and has not shown survival benefit [9]. Systemic therapies for uveal melanoma, generally chosen based on the experiences in cutaneous melanoma, have been associated with modest efficacy in most clinical trials [10]. However, the growing availability of targeted agents for cancer therapy and the recent advances in our understanding of the molecular biology of uveal melanoma have fueled renewed efforts to study novel systemic therapies in this disease [11].

Sorafenib is a multi-kinase inhibitor with anti-proliferative and anti-angiogenic effects via inhibition of several receptor tyrosine kinases including the vascular endothelial growth factor receptors (VEGFR) −1, −2, −3, and platelet derived growth factor receptors (PDGFR) −α, −β; it also inhibits the mitogen-activated protein kinase (MAPK) pathway at the level of Raf kinases [12]. The MAPK signaling (Ras-Raf-MEK-ERK) pathway, which mediates cellular responses to growth signals [13], appears to be constitutively active in most uveal melanoma tumors [14]. Preclinical studies suggested that sorafenib, besides inhibiting ERK phosphorylation [12], also enhanced the antitumor activity of chemotherapy [15], [16]. Subsequently, the combination of sorafenib with chemotherapy agents, including dacarbazine, taxanes, and platinum-compounds was shown to be well-tolerated [17], [18], [19], [20], [21]. In a phase I trial of carboplatin and paclitaxel (CP) plus sorafenib for patients with advanced solid tumors, 105 patients had advanced refractory melanoma. Among this subset, the overall response rate (ORR) was 27%, with 26 partial responses (PR) and one complete response (CR), and the median progression-free survival (PFS) was 8.8 months [22]. These results supported the rationale for investigation of sorafenib plus CP in patients with metastatic uveal melanoma. While this study was being conducted, results of a large randomized phase III trial testing the activity of CP plus sorafenib as second-line therapy for patients with advanced melanoma (uveal melanoma was excluded) demonstrated little contribution of sorafenib to the regimen [23]. In this article, we report the final results of the phase II trial of sorafenib in combination with carboplatin and paclitaxel for treatment of patients with metastatic uveal melanoma.

## Patients and Methods

The protocol for this trial and supporting TREND checklist are available as supporting information; see Checklist S1 and Protocol S1.

### Patient Eligibility

Eligible patients were at least 18 years of age with histologically confirmed stage IV metastatic uveal melanoma and could have received up to one prior systemic therapy. Additional eligibility criteria were Zubrod performance status of 0 or 1; measurable disease as defined per Response Evaluation Criteria in Solid Tumors (RECIST) v1.0 [24]; adequate bone marrow, hepatic, and renal function. Patients were excluded if they had uncontrolled hypertension (defined as blood pressure greater than 140/90 mm Hg), had a bleeding/coagulation disorder or required anticoagulation, had received more than one prior regimen in the metastatic setting, or had received prior treatment with inhibitors of the Raf/Ras or VEGF pathways.

### Study Treatment and Monitoring

The study, registered with ClinicalTrials.gov Identifier as NCT00329641, was activated in March, 2006 and closed to accrual January, 2009. This phase II single-arm study was conducted by the SWOG cancer clinical trials cooperative group, with patients enrolled at 7 member sites. Patients were informed of the investigational nature of the study and signed a written informed consent. The study was approved by the ethics committee or institutional review board at each participating site and complied with the provisions of the Declaration of Helsinki, Good Clinical Practice guidelines, and local laws and regulations.

Eligible patients received carboplatin (initial dose area under curve [AUC] = 6, using the Cockcroft-Gault formula for calculating creatinine clearance [25]) and paclitaxel (initial dose 225 mg/m^2^) administered intravenously on day 1, plus sorafenib (initial dose 400 mg orally twice daily or BID) on days 2–19 of each 3-week cycle. Patients could receive up to 6 cycles of CP plus sorafenib. After discontinuation of CP (for either completion of planned 6 cycles of therapy, excess toxicity or patient request), sorafenib monotherapy (administered on days 1–21) could be continued in those patients whose disease status was stable disease (SD) or better until the occurrence of unacceptable toxicity, disease progression, or death. Guidelines for dose adjustments and dose interruptions to CP or sorafenib, as directed by the most likely attribution of toxicities, were detailed in the study protocol. Up to two dose-reductions were allowed in CP (to C[AUC = 5] plus P[175 mg/m2] or to C[AUC = 4] plus P[150 mg/m2]). The dose of sorafenib could be reduced to 200 mg twice daily or to 200 mg once daily.

Restaging radiologic evaluation was performed at baseline and then every 2 cycles (6 weeks) during administration of CP plus sorafenib, and every 3 cycles (9 weeks) during sorafenib monotherapy. Disease progression and tumor response were evaluated by the investigators using RECIST v1.0 guidelines [24]. An objective (partial or complete) response had to be confirmed with a subsequent radiologic evaluation at least 4 weeks after the preceding evaluation. Patients were monitored for toxicity weekly during the first cycle of therapy, and then prior to each cycle of therapy. Adverse events were graded according to National Cancer Institute Common Toxicity Criteria for Adverse Events, version 3.0.

### Statistical Methods

The primary objective of the study was to assess the efficacy of CP plus sorafenib in this patient population, as measured by the overall objective response rate (ORR) that was defined as the proportion of patients with confirmed complete or partial response. Per the study design, the clinical efficacy of the CP plus sorafenib regimen was to be considered as insufficient if the true ORR was less than 5% (null hypothesis) or of considerable interest if the true ORR was 20% or higher (alternative hypothesis). A two-stage design was used with 20 eligible and evaluable patients to be enrolled in the first stage [26]. If no confirmed responses were observed in the first 20 evaluable patients, the study would be closed to further accrual and the regimen concluded to be inactive. If one or more confirmed responses were observed in the first 20 eligible patients, an additional 20 eligible patients would be accrued to better estimate the true ORR of this regimen. Five or more responses among 40 eligible patients were to be considered as evidence of sufficient activity to support further investigation of this regimen for metastatic uveal melanoma. This design had a power of 92% and a type-1 error of 5%. Assuming that 10% of the enrolled patients will be ineligible or not evaluable for responses, it was expected to accrue around 23 patients to the first phase of the study. The patients who were already in screening at the completion of expected accrual were allowed to register and participate in the study.

Progression-free survival (PFS) and overall survival (OS) were also analyzed as secondary endpoints of the study. PFS was defined as the time from the date of enrollment until the first date of documented disease progression (per RECIST), symptomatic deterioration, or death due to any cause. Patients last known to be alive and progression-free were censored at the date of last contact. Overall survival (OS) was defined as the time from the date of enrollment until the date of death due to any cause. Patients last known to be alive were censored at the date of last contact. OS and PFS estimates were calculated using the Kaplan-Meier method [27] and 95% confidence intervals (CIs) for the medians were constructed using the Brookmeyer and Crowley method [28].

## Results

### Patient characteristics and treatment administration

A total of 25 patients were enrolled in the first stage of the study. One patient never received protocol therapy due to discovery of new brain metastases shortly after enrollment, and was not considered evaluable for the study endpoints. Demographic data for the 24 evaluable patients are shown in **Table 1**. The majority of patients had elevated LDH (63%) and liver metastases (83%), which are associated with poor prognosis for this disease.

**Table 1:** Baseline Characteristics and Demographics of 24 Evaluable Patients.

AGE (in years)		
Median (Range)	61	(48–73)
SEX		
Males	12	50%
Females	12	50%
RACE		
Caucasian	24	100%
PERFORMANCE STATUS		
0	18	75%
1	5	21%
Missing	1	4%
ELEVATED LDH		
No	9	38%
Yes	15	63%
SITE(S) of DISTANT METASTASES		
Bone	4	17%
Brain	1	4%
Liver	20	83%
Lung	8	33%
Distant nodes, skin, soft tissue	4	17%
Other visceral	3	13%
PRIOR SYSTEMIC TREATMENT FOR ADVANCED DISEASE		
None	20	83%
Chemotherapy	4	17%
Biologic/Immunotherapy	0	0%
TIME (in years) FROM INITIAL DIAGNOSIS TO FIRST DIAGNOSIS OF STAGE IV DISEASE		
Median (Range)	3.8	(0–25)

Eight patients completed all six cycles of CP (this included one patient who erroneously received 11 cycles of sorafenib plus CP followed by 18 more cycles of sorafenib monotherapy, despite having disease progression after the first 6 cycles of sorafenib plus CP). Seven patients received sorafenib monotherapy after CP was discontinued for either completion of the planned 6 cycles of therapy (n = 6) or excess toxicity attributable to CP (n = 1); the median number of cycles of sorafenib monotherapy received by these 7 patients was 9 [range: 1–48].

### Efficacy

**Figure 1:**
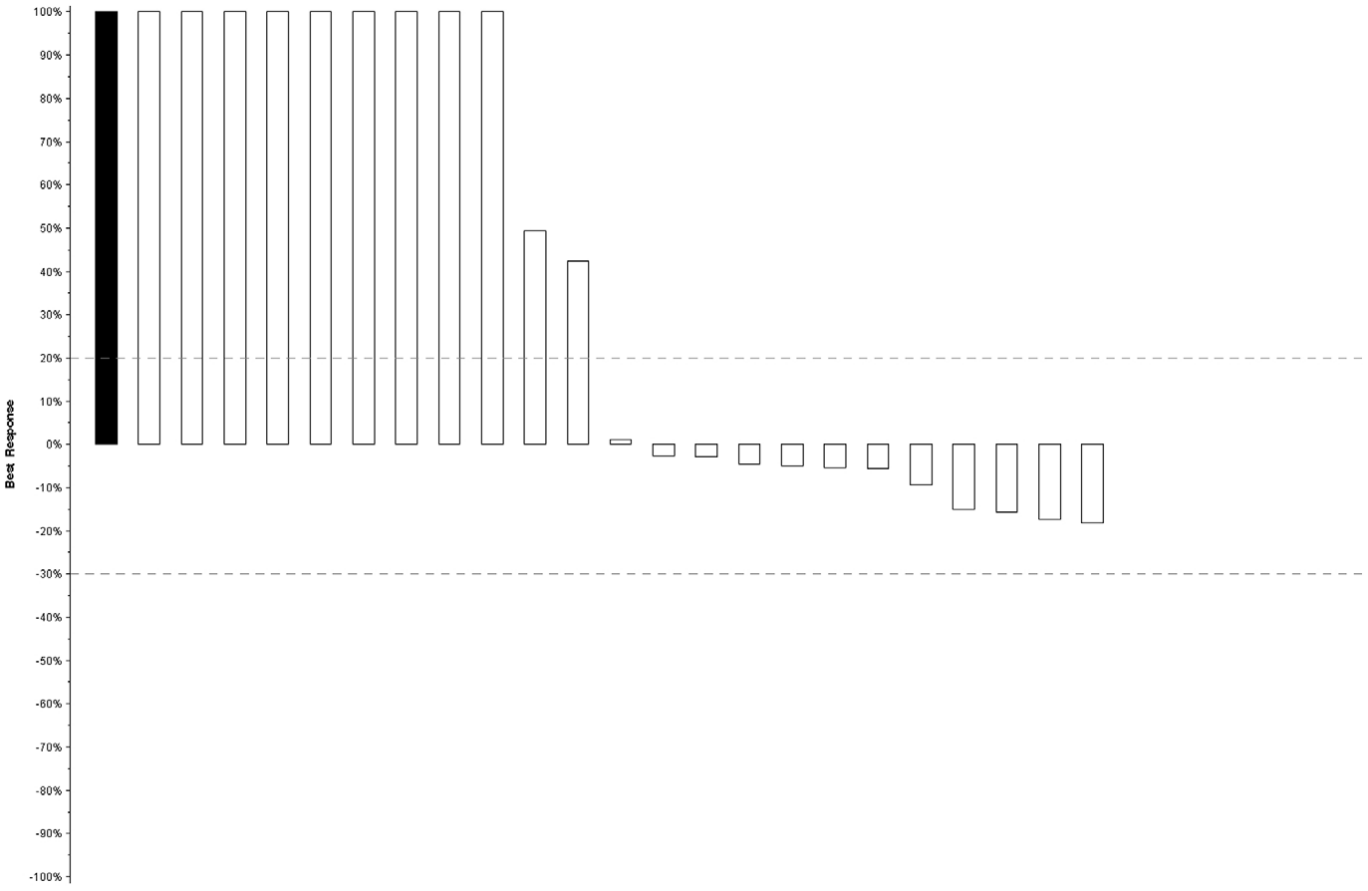
Best tumor response (waterfall plot) in evaluable patients (n = 24). The bars on each plot represent the largest decrease under baseline of the sum of longest diameters of all target measurable lesions, or if no decrease was observed, the smallest increase in the sum of longest diameters of target measurable lesions. Patients whose best response was progression due to new lesions, death (due to disease), or clear worsening of non-measurable disease are represented by a bar showing a 100% increase. In addition, patients whose best response could not be determined due to inadequate assessment are represented on the far left side of the plot as a solid bar showing 100% increase.

**Figure 2:**
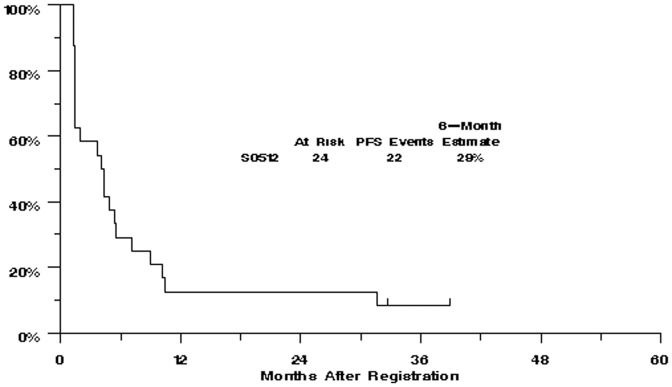
Kaplan-Meier curve for progression-free survival in evaluable patients (n = 24).

**Figure 3:**
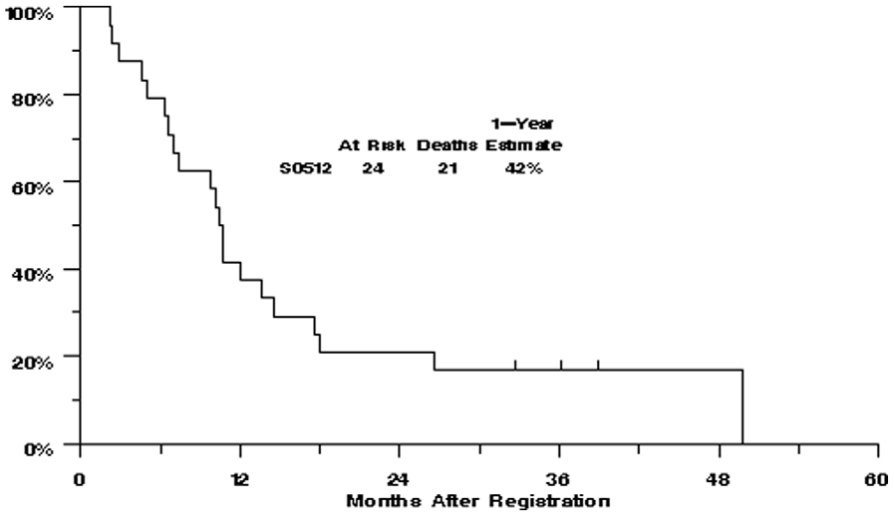
Kaplan-Meier curve for overall survival in evaluable patients (n = 24).

There were no confirmed objective tumor responses by RECIST criteria among the first 24 patients on study (ORR = 0% [95% CI: 0–14%]), which led to the clinical trial not proceeding to the second stage. **Figure 1** provides a waterfall plot of each patient's best response (i.e., maximum reduction or minimum increase in the sum of the longest tumor diameters). Minor responses (tumor regression less than 30%, or unconfirmed tumor regression) were seen in eleven of 24 (45%) patients. The median PFS was 4 months [95% CI: 1–6 months] and the 6-month PFS was 29% [95% CI: 13%–48%] in this study (**Figure 2** **)**. The median OS was 11 months [95% CI: 7–14 months] and the estimated one-year OS was 42% [95% CI: 22%–60%] (**Figure 3**).

The observed 6-month PFS of 29% in this trial was not significantly better than the predicted 6-month PFS rate of 17% based on the model presented in the Korn metaanalysis [29] (p = 0.18). Similarly, the observed one-year overall survival of 42% was not significantly better than the predicted one-year OS of 24% (p = 0.07). However, it should be kept in mind that the applicability of the Korn metaanalysis to uveal melanoma patients is unknown at this time.

A few patients had prolonged stable disease. One such intriguing patient received 5 cycles of CP plus sorafenib followed by sorafenib monotherapy for an additional 39 months. CP was discontinued after 5 cycles due to myelosuppression. Also, the dose of sorafenib had to be reduced to 400 mg/day during the second cycle of CP due to skin rash. After discontinuation of CP, she continued to receive sorafenib monotherapy at the reduced dose of 400 mg/day for 12 months, but was then noted to have slight progression in the size of her tumors (still SD per RECIST). At this time, the sorafenib dose was escalated back to the full dose of 800 mg/day without any recurrence of rash, suggesting that the rash was attributable to CP plus sorafenib combination. The patient remained progression-free on full dose sorafenib for an additional 27 months.

### Toxicities

Toxicities were reported using CTCAE version 3.0. Grades 3 and higher adverse events are summarized in **Table 2**. Seven of the 24 patients (29%) experienced Grade 4 adverse events, all hematologic. Three patients discontinued protocol therapy due to toxicity, all during the first 6 cycles of chemotherapy due to myelosuppression (n = 2) or neuropathy (n = 1). Eighteen patients (75%) required dose modifications in either CP or sorafenib or both due to toxicity. As mentioned earlier, one patient whose sorafenib was reduced because of grade 3 rash was able to tolerate a re-escalation to full dose sorafenib after the cytotoxic agents were discontinued, without recurrence of the rash.

**Table 2:** Summary of Grade 3 or 4 Treatment-related Adverse Events.

NCI CTCAE 3.0 Category/Term	Grade 3 Number	Grade 4 Number
**Any event**	12	7
**Ocular/Visual**		
Blurred vision	1	0
Cataract	1	0
**Constitutional**		
Fatigue	1	0
**Gastrointestinal**		
Diarrhea	2	0
Mucositis, functional: pharynx	1	0
**Metabolic/Laboratory**		
Hypocalcemia	1	0
**Blood/Bone Marrow**		
Hemoglobin	1	1
Leukocytes	2	2
Lymphopenia	1	1
Neutrophils	4	6
Platelets	3	1
**Neurology**		
Neuropathy-sensory	2	0
**Infection**		
Febrile neutropenia	0	1
Urinary Tract Infection with Grade 3–4 ANC	1	0
**Dermatology/Skin**		
Pruritus	1	0
Rash	5	0

*Abbreviations*: ANC, Absolute Neutrophil Count; CTCAE, Common Terminology Criteria for Adverse Events; NCI, National Cancer Institute.

## Discussion

This phase II clinical trial investigated the activity of sorafenib in combination with carboplatin plus paclitaxel in patients with metastatic uveal melanoma. Among 24 evaluable patients enrolled in the first stage of the study, several patients experienced a minor response or stable disease, but there were no confirmed objective responses and the antitumor activity did not meet the predefined threshold to proceed to the second stage of the study. The toxicity profile was consistent with that previously reported for this combination [23].

There are several limitations to interpreting data from this small non-randomized phase II clinical trial in an uncommon disease. The single-arm phase II design to assess efficacy of a novel regimen is best suited for disease settings where the behavior of historical controls is predictable and stable over time, the study population is less likely to be heterogeneous, and the desired therapeutic effect of study intervention is large. Also, the choice of ORR as the primary endpoint for determination of efficacy is less well-suited for a study involving a cytostatic therapy such as sorafenib. Furthermore, the logistics of conducting a study with large number of patients are fairly challenging in uveal melanoma and restrict the sample size, which in turn can adversely affect the false-positive and false-negative rates of the study. Nevertheless, the successful conduct of our study highlights the feasibility of investigating new therapies through the cooperative group mechanisms for a low-prevalence cancer like uveal melanoma.

The results of this study raise doubts about the suitability of sorafenib to target the biological pathways that are implicated in pathogenesis of uveal melanoma. While the MAPK pathway is constitutively active in most uveal melanomas [14], it is clear from the cutaneous melanoma experience that sorafenib is, at best, only a weak inhibitor of this pathway at doses that are administered clinically [23]. Also, the mechanisms of activation of MAPK-pathway in uveal melanoma appear to be distinct from cutaneous melanoma, as mutations in *NRAS* or *BRAF* are rarely found [30]. Instead, mutually exclusive mutations in *GNAQ* and *GNA11* (genes encoding small GTPases, which usually mediate signals from G-coupled receptors) that lead to the constitutive activation of several downstream signaling pathways including the MAPK-pathway have been implicated in the pathogenesis of the majority (greater than 80%) of uveal melanomas [31], [32]. The recent discovery of paradoxical activation of the MAPK-pathway with RAF inhibitors in *BRAF*-wild type tumors [33] raises the possibility that the use of a RAF inhibitor (sorafenib) could even have been counter-productive in uveal melanoma with *GNAQ/GNA11* mutations, and may have negated the potential therapeutic benefits of sorafenib through mechanisms other than MAPK-inhibition. Nevertheless, a subset of patients did appear to have clinical benefit from CP plus sorafenib and from subsequent sorafenib monotherapy. This benefit mostly manifested as stabilization of disease (in contrast to the rapid-onset major tumor regressions seen with mutant-BRAF inhibitors in cutaneous melanoma [34]) raising the speculation that it may have been mediated by mechanisms other than inhibition of the MAPK-pathway. Indeed, there is increasing evidence of the importance of neo-angiogenesis in pathogenesis of uveal melanoma [35], [36], [37], [38] and it is plausible that the anti-angiogenic effects of sorafenib contributed to the disease stabilization and minor tumor responses observed in these patients.

The modest activity of this regimen may also have implications for the optimal design of future regimens for uveal melanoma, especially regarding the inclusion of cytotoxic chemotherapy. The efficacy of systemic cytotoxic chemotherapy against uveal melanoma has been modest in most clinical trials [39]. In spite of the differences in biology and chemosensitivity profile of the two malignancies [40], [41], [42], the chemotherapy regimens for uveal melanoma have been mostly extrapolated from cutaneous melanoma, with disappointing results [39]. It is possible that the inclusion of CP may have confounded the observed activity of sorafenib in uveal melanoma via the increased frequency of dose-reductions in sorafenib when administered as part of the combination (the rate of sorafenib dose reductions was 13% in monotherapy [43] and 33% in CP plus sorafenib [23] in phase III trials). This hypothesis is supported by the observations in one study patient who required a dose-reduction in sorafenib to 400 mg/day due to skin rash while receiving CP plus sorafenib, but was able to tolerate the full dose of sorafenib monotherapy after discontinuation of CP. This patient had also started to have early progression of disease while receiving the reduced dose of sorafenib; an escalation of sorafenib dose to 800 mg/day reversed the early progression and resulted in prolonged SD (greater than 3 years). Hence, we suggest that the possible adverse impact of cytotoxic chemotherapy on a promising investigational therapy should be considered carefully while designing therapeutic regimens for this uncommon malignancy.

## Conclusions

The overall efficacy of CP plus sorafenib in metastatic uveal melanoma did not warrant further clinical testing of this combination when assessed by objective response rates, although minor tumor responses and prolonged stable disease were observed in a few patients. There continues to be a need for development of effective therapies for this life-threatening disease and for personalization of therapy to match the unique biology of each patient's disease.

## Supporting Information

Figure S1 **Consort Diagram.**
(DOC)

Protocol S1 **Trial Protocol.**
(PDF)

Checklist S1 **TREND Checklist.**
(PDF)
