# Supplementary material for: Phase II Trial of Sorafenib in Combination with Carboplatin and Paclitaxel in Patients with Metastatic Uveal Melanoma: SWOG S0512
Source: PLoS One. 2012 Nov 30;7(11):e48787. doi: 10.1371/journal.pone.0048787 (PMC3511501; doi:10.1371/journal.pone.0048787)
Supplement: Figure S1 — Consort Diagram. (DOC) [file pone.0048787.s001.doc]

**Supplemental Figure 1: Consort Diagram for S0512**

Enrollment (n=25)

♦  Not Treated (n=1)

Completed 6 Cycles of Carboplatin + Paclitaxel (n=8)

Off Treatment (n=3)

♦ Disease Progression (n=3)

Off Treatment (n=15)

♦ Disease Progression (n=12)

♦ Toxicity (n=3)

Sorafenib + Carboplatin + Paclitaxel

(n=24)

Maintenance Sorafenib (n=6)

(includes one patient who received only 5 cycles of Carboplatin + Paclitaxel)

Off Treatment (n=5)

♦ Disease Progression (n=4)

♦ Other (n=1)

On Treatment (n=1)

Currently 54 total cycles of treatment including 5 cycles of Carboplatin + Paclitaxel
