# Supplementary material for: Phase II Trial of Sorafenib in Combination with Carboplatin and Paclitaxel in Patients with Metastatic Uveal Melanoma: SWOG S0512
Source: PLoS One. 2012 Nov 30;7(11):e48787. doi: 10.1371/journal.pone.0048787 (PMC3511501; doi:10.1371/journal.pone.0048787)
Supplement: Protocol S1 — Trial Protocol. (PDF) [file pone.0048787.s002.pdf]

Southwest Oncology Group
A National Clinical Research Group

July 1, 2007

TO: ALL SOUTHWEST ONCOLOGY GROUP MEMBER, CCOP, UCOP AND AFFILIATE MEDICAL ONCOLOGISTS, SURGEONS AND PATHOLOGISTS

FROM: Southwest Oncology Group Operations Office

RE: IND Safety Report for BAY 43-9006

MEMORANDUM

IRB Review Requirements

( ) Full board review required. Reason:
- ( ) Initial activation (should your institution choose to participate)
- ( ) Increased risk to patient
- ( ) Complete study redesign
- ( ) Addition of tissue banking requirements
- ( ) Study closure due to new risk information

(√) Expedited review allowed

( ) No review required

MEMORANDUM

The following new safety report has been posted regarding an adverse event that occurred in association with the drug BAY 43-9006. Please access this safety report via the study's abstract page or the safety report link on the Southwest Oncology Group website (https://swog.org/safetyreports/safetyreports.asp).

This safety report pertains to the following studies:	Report:	
**S0412** Genitourinary	June 11, 2007	AE #1117502
**S0434** Myeloma		
**S0435** Lung		
**S0438** Melanoma		
**S0505** Sarcoma		
**S0512** Melanoma		

Protocol amendments are not necessary at this time, but your consent form may be revised to include the information provided in this report if it is deemed necessary by your institution. Please append this notice and this report to your copy of the protocol and forward a copy to your Institutional Review Board (IRB) as required by your local policies and procedures. Should any further information regarding this adverse event be made available, it will be forwarded to you.

This memorandum serves to notify the NCI and Southwest Oncology Group Statistical Center.

cc: PROTOCOL & INFORMATION OFFICE
John J. Crowley, Ph. D.
Antje Hoering, Ph.D.
Michael LeBlanc, Ph.D.
P. Y. Liu, Ph.D.
Mary W. Redman, Ph.D.
Cathy M. Tangen, Dr.P.H.
Vanessa Bolejack, M.P.H.
Bryan Goldman, M.S.
Kari Chansky, M.S.
Mike A. Hussey, M.S.
James Moon, M.S.
Cathryn Rankin, M.S.
Monica Toth, M.S.
Camille White, C.C.R.P.
Larry Kaye
Jean Barce
Amy Edwards
Stephanie Edwards
Lisa Gavigan
Scott Kurruk
Janice Leaman
Brian Zeller

Southwest
Oncology Group
A National Clinical Research Group

June 15, 2007

TO: ALL SOUTHWEST ONCOLOGY GROUP MEMBER, CCOP, UCOP AND AFFILIATE MEDICAL ONCOLOGISTS, SURGEONS AND PATHOLOGISTS

FROM: Southwest Oncology Group Operations Office

RE: IND Safety Report for BAY 43-9006

MEMORANDUM

IRB Review Requirements

( ) Full board review required. Reason:
- ( ) Initial activation (should your institution choose to participate)
- ( ) Increased risk to patient
- ( ) Complete study redesign
- ( ) Addition of tissue banking requirements
- ( ) Study closure due to new risk information

(√) Expedited review allowed

( ) No review required

MEMORANDUM

The following new safety reports have been posted regarding adverse events that occurred in association with the drug BAY 43-9006. Please access these safety reports via the study's abstract page or the safety report link on the Southwest Oncology Group website (https://swog.org/safetyreports/safetyreports.asp).

These safety reports pertain to the following studies:

S0412 Genitourinary
S0434 Myeloma
S0435 Lung
S0438 Melanoma
S0505 Sarcoma
S0512 Melanoma

Reports:

May 25, 2007	AE #1273968
May 25, 2007	AE #1333296(1)
May 27, 2007	AE #1989015

Protocol amendments are not necessary at this time, but your consent form may be revised to include the information provided in these reports if it is deemed necessary by your institution. Please append this notice and these reports to your copy of the protocol and forward copies to your Institutional Review Board (IRB) as required by your local policies and procedures. Should any further information regarding these adverse events be made available, it will be forwarded to you.

This memorandum serves to notify the NCI and Southwest Oncology Group Statistical Center.

cc: PROTOCOL & INFORMATION OFFICE
John J. Crowley, Ph. D.
Antje Hoering, Ph.D.
Michael LeBlanc, Ph.D.
P. Y. Liu, Ph.D.
Mary W. Redman, Ph.D.
Cathy M. Tangen, Dr.P.H.
Vanessa Bolejack, M.P.H.
Bryan Goldman, M.S.
Kari Chansky, M.S.
Mike A. Hussey, M.S.
James Moon, M.S.
Cathryn Rankin, M.S.
Monica Toth, M.S.
Camille White, C.C.R.P.
Larry Kaye
Jean Barce
Amy Edwards
Stephanie Edwards
Lisa Gavigan
Scott Kurruk
Janice Leaman
Brian Zeller

Operations Office
14980 Omicron Drive • San Antonio, TX 78245-3217 • Telephone 210-450-8808 • FAX 210-677-0006 • http://www.swog.org

Southwest
Oncology Group
A National Clinical Research Group

May 1, 2007

TO: ALL SOUTHWEST ONCOLOGY GROUP MEMBER, CCOP, UCOP AND AFFILIATE MEDICAL ONCOLOGISTS, SURGEONS AND PATHOLOGISTS

FROM: Southwest Oncology Group Operations Office

RE: IND Safety Report for BAY 43-9006

MEMORANDUM

IRB Review Requirements

( ) Full board review required. Reason:
- ( ) Initial activation (should your institution choose to participate)
- ( ) Increased risk to patient
- ( ) Complete study redesign
- ( ) Addition of tissue banking requirements
- ( ) Study closure due to new risk information

(√) Expedited review allowed

( ) No review required

MEMORANDUM

The following new safety report has been posted regarding an adverse event that occurred in association with the drug BAY 43-9006. Please access this safety report via the study's abstract page or the safety report link on the Southwest Oncology Group website (https://swog.org/safetyreports/safetyreports.asp).

This safety report pertains to the following studies:

S0412 Genitourinary
S0434 Myeloma
S0435 Lung
S0505 Sarcoma
S0512 Melanoma

Report:

April 11, 2007 AE #1893074

Protocol amendments are not necessary at this time, but your consent form may be revised to include the information provided in this report if it is deemed necessary by your institution. Please append this notice and this report to your copy of the protocol and forward a copy to your Institutional Review Board (IRB) as required by your local policies and procedures. Should any further information regarding this adverse event be made available, it will be forwarded to you.

This memorandum serves to notify the NCI and Southwest Oncology Group Statistical Center.

cc: PROTOCOL & INFORMATION OFFICE
John J. Crowley, Ph. D.
Antje Hoering, Ph.D.
Michael LeBlanc, Ph.D.
P. Y. Liu, Ph.D.
Mary W. Redman, Ph.D.
Cathy M. Tangen, Dr.P.H.
Vanessa Bolejack, M.P.H.
Bryan Goldman, M.S.
Kari Chansky, M.S.
Mike A. Hussey, M.S.
James Moon, M.S.
Cathryn Rankin, M.S.
Monica Toth, M.S.
Camille White, C.C.R.P.
Larry Kaye
Jean Barce
Amy Edwards
Stephanie Edwards
Lisa Gavigan
Scott Kurruk
Janice Leaman
Brian Zeller

Southwest
Oncology Group
A National Clinical Research Group

March 15, 2007

TO: ALL SOUTHWEST ONCOLOGY GROUP MEMBER, CCOP, UCOP AND AFFILIATE MEDICAL ONCOLOGISTS, SURGEONS AND PATHOLOGISTS

FROM: Southwest Oncology Group Operations Office

RE: IND Safety Reports for BAY 43-9006

MEMORANDUM

IRB Review Requirements

( ) Full board review required. Reason:
- ( ) Initial activation (should your institution choose to participate)
- ( ) Increased risk to patient
- ( ) Complete study redesign
- ( ) Addition of tissue banking requirements
- ( ) Study closure due to new risk information

(√) Expedited review allowed

( ) No review required

MEMORANDUM

The following new safety reports have been posted regarding adverse events that occurred in association with the drug BAY 43-9006. Please access these safety reports via the study's abstract page or the safety report link on the Southwest Oncology Group website (https://swog.org/safetyreports/safetyreports.asp).

These safety reports pertain to the following studies:

S0412 Genitourinary
S0420 Head and Neck
S0434 Myeloma
S0435 Lung
S0505 Sarcoma
S0512 Melanoma
S0514 Gastrointestinal

Reports:

January 9, 2007	AE #1447378
January 19, 2007	AE #1333296
January 26, 2007	AE #1007443

Protocol amendments are not necessary at this time, but your consent form may be revised to include the information provided in these reports if it is deemed necessary by your institution. Please append this notice and these reports to your copy of the protocol and forward copies to your Institutional Review Board (IRB) as required by your local policies and procedures. Should any further information regarding these adverse events be made available, it will be forwarded to you.

This memorandum serves to notify the NCI and Southwest Oncology Group Statistical Center.

cc: PROTOCOL & INFORMATION OFFICE
John J. Crowley, Ph. D.
Jacqueline Benedetti, Ph.D.
Antje Hoering, Ph.D.
Michael LeBlanc, Ph.D.
P. Y. Liu, Ph.D.
Mary W. Redman, Ph.D.
Cathy M. Tangen, Dr.P.H.
Vanessa Bolejack, M.P.H.
Bryan Goldman, M.S.
Kari Chansky, M.S.
Mike A. Hussey, M.S.
James Moon, M.S.
Cathryn Rankin, M.S.
Monica Toth, M.S.
Camille White, C.C.R.P.
Larry Kaye
Jean Barce
Jennie Barrett
Amy Edwards
Stephanie Edwards
Lisa Gavigan
Scott Kurruk
Janice Leaman
Christine McLeod
Rodney Sutter
Brian Zeller

Southwest Oncology Group
A National Clinical Research Group

Distribution Date: September 15, 2006
CTEP Submission Date: July 6, 2006
Date Action Letter Distributed: June 27, 2006

TO: ALL SOUTHWEST ONCOLOGY GROUP MEMBER, CCOP AND AFFILIATE MEDICAL ONCOLOGISTS AND PATHOLOGISTS

FROM: Katherine "Katy" Robert, Protocol Coordinator

RE: **S0512**, "Phase II Trial of BAY 43-9006 (Sorafenib; NSC-724772) in Combination with Carboplatin and Paclitaxel in Patients with Metastatic Uveal Melanoma". Study Coordinator: Dr. A. Aparicio.

AMENDMENT #2

Study Coordinator: Ana Aparicio, M.D.
Phone: 713/563-6969
E-mail: aaparicio@mdanderson.org

IRB Review Requirements

(√) Full board review required. Reason:
- ( ) Initial activation (should your institution choose to participate)
- (√) Increased risk to patient
- ( ) Complete study redesign
- ( ) Addition of tissue banking requirements
- ( ) Study closure not built into study design
- ( ) Study closure due to new risk information

( ) Expedited review allowed

( ) No review required

AMENDMENT #2

This amendment is response to a memo from Dr. J. Wright (wright@ctep.nci.nih.gov, Phone: 301/496-1196) regarding the development of GI-Perforation (NOS). The above-referenced protocol has been amended as follows:

1. Title Page: Dr. Aparicio's contact information has been updated. The version date has also been revised.

2. Pages 10 - 12a, Section 3.1b: The Comprehensive Adverse Events and Potential Risks (CAEPR) list has been amended as a result of an IND Safety Report for BAY 43-9006. The following adverse event has been added to the updated version 2.1 CAEPR for this drug under the "Rare but Serious" category: GI Perforation (NOS).

3. Page 21, Section 7.0 and Page 29, Section 8.7: Dr. Aparicio's contact information has been updated.

4. Page 27, Section 8.3l: This section has been inserted to outline the discontinuation of protocol treatment in the event of the aforementioned risk.

5. Page 58a, Model Consent Form: "Tear in stomach/intestines that may be painful and possibly fatal" has been added to the Rare but Serious risks as a result of the updated Version 2.1 CAEPR for BAY 43-9006. Institutions must update their consent forms to include this information for future registration. Patients currently being treated on this study must be informed of this information in the manner determined by the local Institutional Review Board (IRB).

Replacements are included for those pages listed above. Please attach a copy of this memorandum to your protocol, insert the replacement pages, and forward to your Institutional Review Board (IRB).

This memorandum serves to notify the NCI and Southwest Oncology Group Statistical Center.

cc: PROTOCOL & INFORMATION OFFICE
P.Y. Liu, Ph.D.
James Moon, M.S.
Brian Zeller

Southwest
Oncology Group
A National Clinical Research Group

June 15, 2006

TO: ALL SOUTHWEST ONCOLOGY GROUP MEMBER, CCOP, UCOP AND AFFILIATE MEDICAL ONCOLOGISTS, SURGEONS AND PATHOLOGISTS

FROM: Southwest Oncology Group Operations Office

RE: IND Safety Reports for BAY 43-9006

MEMORANDUM

IRB Review Requirements

( ) Full board review required. Reason:
- ( ) Initial activation (should your institution choose to participate)
- ( ) Increased risk to patient
- ( ) Complete study redesign
- ( ) Addition of tissue banking requirements
- ( ) Study closure due to new risk information

(√) Expedited review allowed

( ) No review required

MEMORANDUM

The following new safety reports have been posted regarding adverse events that occurred in association with the drug BAY 43-9006. Please access these safety reports via the study's abstract page or the safety report link on the Southwest Oncology Group website (https://swog.org/safetyreports/safetyreports.asp).

These safety reports pertain to the following studies:

S0412 Genitourinary
S0420 Head and Neck
S0434 Myeloma
S0435 Lung
S0505 Sarcoma
S0512 Melanoma
S0514 Gastrointestinal

Reports:

May 16, 2006 AE #1900831
May 22, 2006 AE #1108237

Protocol amendments are not necessary at this time, but your consent form may be revised to include the information provided in these reports if it is deemed necessary by your institution. Please append this notice and these reports to your copy of the protocol and forward copies to your Institutional Review Board (IRB) as required by FDA regulation. Should any further information regarding these adverse events be made available, it will be forwarded to you.

This memorandum serves to notify the NCI and Southwest Oncology Group Statistical Center.

cc: PROTOCOL & INFORMATION OFFICE
John J. Crowley, Ph. D.
Jacqueline Benedetti, Ph.D.
Antje Hoering, Ph.D.
Michael LeBlanc, Ph.D.
P. Y. Liu, Ph.D.
Mary W. Redman, Ph.D.
Cathy M. Tangen, Dr.P.H.
Bryan Goldman, M.S.
Kari Chansky, M.S.
Mike A. Hussey, M.S.
James Moon, M.S.
Cathryn Rankin, M.S.
Monica Toth, M.S.
Camille White, B.S., C.C.R.P.
Larry Kaye, B.A.
Jean Barce
Jennie Barrett
Amy Edwards
Stephanie Edwards
Lisa Gavigan
Scott Kurruk
Janice Leaman
Christine McLeod
Rodney Sutter
Brian Zeller

Distribution Date: June 15, 2006
CTEP Submission Date: June 1, 2006

TO: ALL SOUTHWEST ONCOLOGY GROUP MEMBER, CCOP AND AFFILIATE MEDICAL ONCOLOGISTS AND PATHOLOGISTS

FROM: Courtney K. Wille, Protocol Coordinator

RE: **S0512**, "Phase II Trial of BAY 43-9006 (Sorafenib; NSC-724772) in Combination with Carboplatin and Paclitaxel in Patients with Metastatic Uveal Melanoma". Study Coordinator: Dr. A. Aparicio.

REVISION #1

Study Coordinator: Ana Aparicio, M.D.
Phone: 323/865-0470
E-mail: aaparici@usc.edu

IRB Review Requirements

( ) Full board review required. Reason:
- ( ) Initial activation (should your institution choose to participate)
- ( ) Increased risk to patient
- ( ) Complete study redesign
- ( ) Addition of tissue banking requirements
- ( ) Study closure not built into study design
- ( ) Study closure due to new risk information

(√) Expedited review allowed

( ) No review required

REVISION #1

The above-referenced protocol has been revised as follows:

1. The Version Date on the Title Page has been updated.

2. Funding for the post-treatment (Day 17) biopsy was not approved. Therefore, the post-treatment biopsy has been removed from the protocol and changes were made to the following sections:

   - Section 1.4 (page 2): This section has been revised to read "To investigate in a preliminary manner whether changes in VEGF levels in plasma and urine and changes in ERK ½ phosphorylation in stimulated lymphocytes can be correlated with response to treatment".

   - Section 2.0, Correlative Studies (page 8): The second sentence in the first paragraph has been revised to read "We propose to study a number of molecular markers in baseline tumor biopsies as well as in pre- and post-treatment blood and urine samples, to explore whether there is a correlation between these markers and response to treatment with this regimen in patients with metastatic uveal melanoma"

- Section 2.0, Correlative Studies (page 8): The fourth sentence in the first paragraph "In patients with accessible..." has been deleted.

- Section 2.0, Correlative Studies (page 8): The sixth sentence in the first paragraph "In patients with accessible tumors..." has been deleted.

- Section 2.0, subheading "Microvessel density in tumor biopsies" (page 8): The last two sentences "We will also explore the possibility..." have been deleted.

- Section 2.0 (page 8): The subheading for ERK ½ phosphorylation has been revised to remove "and tumor biopsies".

- Section 2.0, subheading "ERK ½ phosphorylation" (page 8): The last two sentences "We will also explore the possibility..." have been deleted.

- Section 2.0, subheading "VEGFR-2 phosphorylation in tumor biopsies" (page 9): This section has been deleted.

- Section 7.8 (page 24): This section has been deleted and the remaining sections were renumbered accordingly.

- Section 9.0 (page 30): The row for biopsy/tissue specimen has been deleted and the "∑" footnote has been revised to delete the last sentence "In patients with accessible tumors...".

- Section 11.5 (page 34): This section has been revised to read "The analysis of the relationship between clinical outcomes and various tumor markers (baseline MVD and changes in VEGF in plasma and urine, as well as ERK ½ phosphorylation in peripheral blood mononuclear cells) will be exploratory and will not generate definitive results. However, they will provide the basis for the generation of hypotheses to be tested in larger patient cohorts"

- Section 15.4, (page 39): The first, third and fourth paragraphs have been deleted and the remaining sections have been renumbered accordingly.

- Section 15.6 (page 40): The first sentence in the first paragraph has been revised to read "Tumor sample sections will be deparaffinized and stained with hematoxylin and eosin (H&E) to visualize tumor architecture"

- Section 15.6 (page 40): The second sentence in the first paragraph has been deleted.

- Section 15.6 (page 40): The fifth sentence in the first paragraph has been revised to read "Antibodies for CD31 will be obtained from Santa Cruz Biotechnology (Santa Cruz, CA)".

- Section 15.6 (page 40): The second paragraph has been deleted.

- Model Informed Consent, "What are the costs of taking part in this study?" (page 60): The last sentence in the first paragraph has been deleted.

- Model Informed Consent, "Specimen Submission" (page 62): The last two sentences in the first paragraph have been deleted.

- Model Informed Consent, "About Using Specimens for Research" (page 63): The sentence "You may also have had another tissue sample taken at Week 3" has been deleted.

Patients who are currently being treated on this study and have given informed consent to the post-treatment (Day 17) biopsy must be informed of this change in the manner determined by the local Institutional Review Board (IRB).

The Title Page with the revised Version Date and revised pages 2, 8, 9, 24, 30, 34, 39, 40, 60, 62 and 63 are enclosed for insertion into your copy of the protocol.

Please append this notice and the attachments to your copy of the protocol.

This memorandum serves to notify the NCI and Southwest Oncology Group Statistical Center.

cc: PROTOCOL & INFORMATION OFFICE
P.Y. Liu, Ph.D.
James Moon, M.S.
Brian Zeller

Southwest
Oncology Group
A National Clinical Research Group

Distribution Datre: April 15, 2006
CTEP Submission Date: March 31, 2006

TO: ALL SOUTHWEST ONCOLOGY GROUP MEMBER, CCOP AND AFFILIATE MEDICAL ONCOLOGISTS AND PATHOLOGISTS

FROM: Courtney K. Watson, Protocol Coordinator

RE: **S0512**, "Phase II Trial of BAY 43-9006 (Sorafenib; NSC-724772) in Combination with Carboplatin and Paclitaxel in Patients with Metastatic Uveal Melanoma". Study Coordinator: Dr. A. Aparicio.

AMENDMENT #1

Study Coordinator: Ana Aparicio, M.D.
Phone: 323/865-0470
E-mail: aaparici@usc.edu

IRB Review Requirements

(√) Full board review required. Reason:
- ( ) Initial activation (should your institution choose to participate)
- (√) Increased risk to patient
- ( ) Complete study redesign
- ( ) Addition of tissue banking requirements
- ( ) Study closure not built into study design
- ( ) Study closure due to new risk information

( ) Expedited review allowed

( ) No review required

AMENDMENT #1

The above-referenced protocol has been amended as follows:

1. The Version Date on the Title Page has been updated.

2. Section 3.1b (pages 10-12) has been amended to update the Comprehensive Adverse Events and Potential Risks (CAEPR) list. "Hemoglobin", "Leukocytes (total WBC)", "Lymphopenia", "Neutrophils/granulocytes (ANC/AGC)" and "Platelets" have been added as possible Blood/Bone Marrow adverse events. "Flushing" has been added as a possible dermatologic adverse event. "Erythema multiforme", "voice changes" and "erectile dysfunction" have been added as reported but with relationship to sorafenib still undetermined. Page 12a has been added to prevent extensive repagination.

3. One cycle in this study should be defined as "21 days" rather than "21 calendar days". Therefore "calendar" has been deleted from the note under the table in Section 7.2 (page 22), the last sentence in Section 7.6 (page 23) and in Section 14.6 (page 37).

4. The spelling of sorafenib has been corrected throughout the Study Calendar in Section 9.0 (page 30).

5. The following risks have been added to the BAY 43-9006 (sorafenib) list of risks that are considered less likely (page 58): sudden reddening of the face and/or neck; decrease in the protein in red blood cells, responsible for carrying oxygen throughout the body; decrease in the total number of white blood cells; decrease in a type of white blood cells; decrease in the number of blood cells that help the blood to clot. Page 58a has been added to prevent extensive repagination. Institutions must update their consent forms to include this information for future registrations. Patients currently being treated on this study must be informed of this information in the manner determined by the local Institutional Review Board (IRB).

The Title Page with the amended Version Date and amended pages 10-12a, 22, 23, 30, 37, 58 and 58a are enclosed for insertion into your copy of the protocol.

Please append this notice and the attachments to your copy of the protocol.

This memorandum serves to notify the NCI and Southwest Oncology Group Statistical Center.

cc: PROTOCOL & INFORMATION OFFICE
P.Y. Liu, Ph.D.
James Moon, M.S.
Brian Zeller

March 15, 2006

TO: ALL SOUTHWEST ONCOLOGY GROUP MEMBER, CCOP AND AFFILIATE MEDICAL ONCOLOGISTS AND PATHOLOGISTS

FROM: Courtney K. Watson, Protocol Coordinator

RE: **S0512**, "Phase II Trial of BAY 43-9006 (Sorafenib; NSC-724772) in Combination with Carboplatin and Paclitaxel in Patients with Metastatic Uveal Melanoma". Study Coordinator: Dr. A. Aparicio.

STATUS NOTICE

Study Coordinator: Ana Aparicio, M.D.
Phone: 323/865-0470
E-mail: aaparici@usc.edu

IRB Review Requirements

(√) Full board review required. Reason:
- (√) Initial activation (should your institution choose to participate)
- ( ) Increased risk to patient
- ( ) Complete study redesign
- ( ) Addition of tissue banking requirements
- ( ) Study closure not built into study design
- ( ) Study closure due to new risk information

( ) Expedited review allowed

( ) No review required

ACTIVATION

The study referenced above is now open for participation. Entire copies of the protocol are enclosed for your use.

This memorandum serves to notify the NCI and Southwest Oncology Group Statistical Center.

cc: PROTOCOL & INFORMATION OFFICE
P.Y. Liu, Ph.D.
James Moon, M.S.
Brian Zeller

PRIVILEGED COMMUNICATION
FOR INVESTIGATIONAL USE ONLY

S0512
Activated March 15, 2006
Amended 7/6/06

SOUTHWEST ONCOLOGY GROUP

PHASE II TRIAL OF BAY 43-9006 (SORAFENIB; NSC-724772) IN COMBINATION WITH CARBOPLATIN AND PACLITAXEL IN PATIENTS WITH METASTATIC UVEAL MELANOMA

		Page
1.0	OBJECTIVES	2
2.0	BACKGROUND	2
3.0	DRUG INFORMATION	9
4.0	STAGING CRITERIA	17
5.0	ELIGIBILITY CRITERIA	18
6.0	STRATIFICATION FACTORS	21
7.0	TREATMENT PLAN	21
8.0	TOXICITIES TO BE MONITORED AND DOSAGE MODIFICATIONS	24
9.0	STUDY CALENDAR	30
10.0	CRITERIA FOR EVALUATION AND ENDPOINT DEFINITIONS	31
11.0	STATISTICAL CONSIDERATIONS	33
12.0	DISCIPLINE REVIEW	34
13.0	REGISTRATION GUIDELINES	34
14.0	DATA SUBMISSION SCHEDULE	35
15.0	SPECIAL INSTRUCTIONS	38
16.0	ETHICAL AND REGULATORY CONSIDERATIONS	41
17.0	BIBLIOGRAPHY	47
18.0	MASTER FORMS SET	51
19.0	APPENDIX	82

PARTICIPANTS: ALL SOUTHWEST ONCOLOGY GROUP MEMBER, CCOP AND AFFILIATE MEDICAL ONCOLOGISTS AND PATHOLOGISTS

STUDY COORDINATORS:

Ana Aparicio, M.D. (Medical Oncology)
GU Medical Oncology - Unit 1374
University of Texas - MD Anderson Cancer Center
1515 Holcombe Avenue
Houston, TX 77030
Phone: 713/563-6969
E-mail: aaparicio@mdanderson.org

Jeffrey S. Weber, M.D., Ph.D. (Medical Oncology)
USC/Norris Cancer Center
1441 Eastlake Avenue
M/S-34 (Suite 3440)
Los Angeles, CA 90089
Phone: 323/865-3962
FAX: 323/865-0061
E-mail: jweber@usc.edu

AGENTS:

BAY 43-9006 (sorafenib; NSC-724772) (IND-69896)
Carboplatin (CBDCA) (NSC-241240)
Paclitaxel, Taxol® (NSC-673089)

James Moon, M.S. (Biostatistics)
P.Y. Liu, Ph.D.
Southwest Oncology Group Statistical Center
at Fred Hutchinson Cancer Research Center
1100 Fairview Avenue North, M3-C102
P.O. Box 19024
Seattle, WA 98109-1024
Phone: 206/667-4623
FAX: 206/667-4408
E-mail: jmoon@fhcrc.org
E-mail: pliu@fhcrc.org

1.0 OBJECTIVES

The primary objective of this study is:

1.1 To assess the response rate (confirmed and unconfirmed, complete and partial responses) of patients with metastatic uveal melanoma treated with BAY 43-9006 (sorafenib) in combination with carboplatin and paclitaxel.

The secondary objectives of this study are:

1.2 To estimate progression-free survival and overall survival of patients with metastatic uveal melanoma treated with BAY 43-9006 (sorafenib) in combination with carboplatin and paclitaxel.

1.3 To assess the toxicity of the combination of BAY 43-9006 (sorafenib), carboplatin and paclitaxel in this patient population.

1.4 To investigate in a preliminary manner whether changes in VEGF levels in plasma and urine and changes in ERK 1/2 phosphorylation in stimulated lymphocytes can be correlated with response to treatment.

2.0 BACKGROUND

Study Disease

Melanoma accounts for 70% of all primary ocular malignancies. Approximately 0.7 new cases per 100,000 population are diagnosed annually in the United States. Intraocular melanomas arise from uveal melanocytes located in the uveal stroma (in the iris, ciliary body and choroids). They metastasize hematogenously, but not via the lymphatic circulation given the absence of lymphatics draining these intraocular compartments. Lymph node metastasis may occur if there is invasion of adjacent tissues that do have lymphatic drainage. This is distinct from melanomas arising in the conjunctiva which do spread regionally through the lymphatic system, similarly to the cutaneous melanomas. Conjunctival melanomas are rare and will not be included in this trial. *(1)*

The risk of metastatic disease is high following a primary diagnosis of uveal melanoma although metastasis may become apparent after a prolonged tumor-free interval. Risk factors associated with an increased risk of metastatic disease include: large size of the tumor, ciliary body location, predominance of epithelioid cells over spindle cells and the presence of vascular networks. *(2, 3)* A recent report on 2,320 patients followed prospectively in Collaborative Ocular Melanoma Study Group trials for large and medium sized uveal melanomas found overall 5- and 10-year cumulative metastatic melanoma rates of 24% and 32%, respectively. The most common sites of metastasis included liver (91%), lung (28%) and bone (18%). Median survival for patients with metastatic disease was less than six months after the diagnosis in this series. *(4)*

In a retrospective review of 91 Finnish patients with metastatic uveal melanoma by Eskelin et al, 80% had only hepatic metastasis at diagnosis and median survival was 8.4 months. *(5)* In this series, three independent predictive markers of survival were obtained by multivariate analysis: the Karnofsky performance status, the largest dimension of the largest metastasis and the alkaline phosphatase level. LDH levels were only associated with survival by univariate analysis, but it was only known for half of the patients analyzed in this cohort. In the MD Anderson experience on 201 patients reported in 1995, only the metastasis-free interval and the serum alkaline phosphatase level appeared to have significant independent influence on survival, out of nine clinical factors analyzed. In most series however, it is the LDH level that maintains a strong prognostic value on multivariate analysis. *(6 - 9)*

Current Therapy of Metastatic Uveal Melanoma

There is no standard of care for patients with metastatic uveal melanomas.

Systemic Therapy

It is often stated that this disease is less responsive to the chemotherapy used for cutaneous melanomas. In the MD Anderson experience reported by Bedikian et al, in 1995, of the 143 patients treated with various combinations of systemic chemotherapy and immunotherapy agents, only one was reported to have a partial response, seven had a minor response and 27 had stable disease. *(10)* However, an analysis of patients with uveal melanomas enrolled in seven Phase II clinical trials conducted by the Southwest Oncology Group suggests that, when adjustment is made for prognostic factors, there is no significant difference in survival or response rates between patients with ocular and non-ocular melanomas. In this series, one complete response and five partial responses were observed in 64 patients with ocular melanoma (9% objective response rate) treated with various systemic agents which was not significantly different from the 11 complete responses and 34 partial responses that were seen among 420 patients with non-ocular melanoma (11% objective response rate) in these trials. *(11)*

Various systemic chemotherapies have been evaluated in Phase II clinical trials designed for patients with metastatic uveal melanomas. The combination of bleomycin, vincristine, lomustine and dacarbazine (BOLD) plus intercycle alpha-interferon-2b was first evaluated in a group of 23 patients between 1992 and 1995, and resulted in four objective responses. *(12)* In another study using a similar regimen (with a higher dose of interferon) 3 of 20 patients evaluated for response had a partial response and 11 patients had stable disease for a median of 7 months. *(7)* In the first trial, significant lung toxicity was noted in several patients while in the second trial two treatment-associated deaths occurred as well as hematological and non-hematological Grade 3-4 toxicity in a substantial proportion of patients.

More recent studies have evaluated the use of novel agents in patients with metastatic uveal melanoma. The combination of gemcitabine and treosulfan (an alkylating agent) has been tested in three clinical trials. In the first Phase I/II trial reported by Pfohler et al, 14 patients were treated, with one achieving a complete response, three a partial response and eight stable disease. *(13)* A second Phase I/II clinical trial by Keilholz et al, administered gemcitabine and treosulfan to 33 patients with uveal melanoma. *(14)* One demonstrated a long-lasting partial response and 44% demonstrated stabilization of disease exceeding 3 months. In the third trial of this combination, gemcitabine was given at a dose of 1 g/m^2 and treosulfan at a dose of 3.5 g/m^2 to previously untreated patients on Days 1 and 8 of 28-day cycles. Pretreated patients (n=8) were given 75% of the dose of both drugs. Twenty patients were evaluated for clinical response. There were no objective responses, but disease was stabilized in five patients (interestingly, all pretreated) and this disease stabilization was associated with a significantly prolonged median survival (512 days versus 181 days for patients with progressive disease). There were no Grade 2-4 non-hematological adverse events and only one instance of Grade 3 anemia, leukopenia and thrombocytopenia each. Finally, a small trial was done with the nucleoside analogue, bendamustine, which has alkylating activity. It was given intravenously to 11 patients as a second-line therapy, but all patients progressed. *(15)*

Locoregional Therapy

Given the fact that up to 80% of patients have disease confined to the liver when first diagnosed with metastatic disease multiple locoregional therapies have been evaluated. *(5, 16)*

Surgery

There have been several anecdotal reports and a 12-patient series of resection of hepatic metastasis resulting in long-lasting disease control. *(17 - 20)* However, the latter 12 patients were selected out of 130 patients with metastatic uveal melanoma registered at the Thomas

Jefferson University Hospital over a 22-year period, speaking for the fact that this approach is only feasible in a very select minority of patients.

Hepatic Artery Chemo-embolization and Immunoembolization

Hepatic artery embolization using chemotherapy or immunotherapy agents has been another approach. In 1988 Mavligit et al, reported their experience using cisplatin and polyvinyl sponge in 30 patients, in which they observed a 46% overall response rate. *(21)* In a more recent Phase I trial using cisplatin, thiotepa and lipiodol, two of three patients with metastatic uveal melanoma achieved partial responses lasting up to 16 months. *(22)* However, toxicity was significant with two patients dying of gram-negative sepsis, one of respiratory failure and one of an acute myocardial infarction shortly after therapy. More exciting is a Phase I/II trial reported in abstract form in 2003, in which 24 patients were treated with a hepatic artery infusion of GM-CSF emulsified in ethiodized oil followed by embolization with gelatin sponge pledgets. Of 21 patient that were evaluable for tumor response at the time of the report, 4 had a partial response and 12 had stable disease, with toxicity limited to mild liver pain and transient elevation of transaminases. Interestingly, 3 of 6 patients with extrahepatic metastasis developed histological evidence of inflammation in the non-liver lesions. *(23)* The efficacy of this approach has yet to be confirmed, however, in a larger series of patients.

Hepatic Intra-arterial Chemotherapy

Out of 38 patients receiving intra-arterial therapy with various agents (including cisplatin, DTIC, IL-2 and others in various combinations) in the MD Anderson experience reported by Bedikian et al, in 1995, two patients achieved a partial response, four patients achieved a minor response and 10 patients had stable disease. *(10)* Eight patients were treated with bi-weekly intra-arterial carboplatin by Cantore et al, with a 38% overall response rate and a median survival of 15 months reported. *(24)*

In a Phase II trial conducted in Switzerland and France, 30 patients underwent laparotomy with resection of major metastasis when feasible and insertion of a catheter into the hepatic artery connected to a subcutaneous access chamber, through which fotemustine was delivered at a dose of 100 mg/m^2 over 4 hours, once a week for four cycles, followed by a maintenance phase with administration every 3 weeks. In this trial, 4 patients achieved a complete response and 8 a partial response, with a median duration of 11 months. A minor response was observed in 2 patients and stable disease in 13. The median survival time was 14 months. Aside from the morbidity associated with the surgical procedure, there were 13 cases of Grade 3-4 hematologic toxicity and two of Grade 3 nausea and vomiting. Transient asymptomatic rises of liver enzymes occurred in 13 patients. Although the results were encouraging, these patients were clearly highly selected. *(6)*

Systemic spillage of fotemustine was evident in the Leyvraz trial based on the occurrence of Grade 3 - 4 myelosuppression. In addition, the low incidence of subsequent extrahepatic disease despite the long survivals achieved by some patients, suggested that the drug might be active systemically. Based on these data Becker et al, conducted a trial using fotemustine intra-arterially if patients had disease limited to the liver, and intravenously in the presence of extrahepatic metastasis. *(8)* This was followed by subcutaneous interleukin-2 and interferon-α in all patients. Forty-eight patients were enrolled of which only 23 had hepatic metastasis and thus received intra-arterial fotemustine. In contrast to the Leyvraz trial, in this study intra-arterial fotemustine was delivered through an angiography catheter in the hepatic artery placed by repeated puncture of the femoral artery over a one-hour infusion on Days 1 and 8 of the first cycle and Day 1 of subsequent cycles. In the 25 patients that received intravenous fotemustine, one achieved a CR and another achieved a PR, while in the cohort of patients receiving intra-arterial fotemustine, 5 PRs were observed. Eighteen patients had stable disease, and this again increased their overall survival when compared to patients with progressive disease. The lower response rate observed in the intra-arterial group compared to the Leyvraz trial can be explained

by the fact that the majority of patients in this trial were diagnosed with disseminated disease due to symptoms, that none of the patients underwent debulking surgery prior to treatment and possibly by the differences in fotemustine dose and administration schedule.
Isolated Hepatic Perfusion

Phase I and II trials using isolated hepatic perfusion have been conducted in the surgery branch of the National Institutes of Health (NIH). In an initial report, twenty-two patients were treated with a 1-hour hyperthermic isolated hepatic perfusion of melphalan with or without Tumor Necrosis Factor (TNF) resulting in an overall response rate of 62% and a median survival of 11 months, with two patients surviving for more than 3 years. TNF did not increase the response rate, but did appear to prolong the duration of response. However, there was significant associated toxicity and one treatment-related death. *(25)* A follow-up study describes 29 patients treated with 1.5 mg/kg of melphalan alone in this manner and confirms an overall response rate of 62% (3 CRs and 15 PRs). The median overall survival in these patients was 12 months. The mean hospitalization duration was 10 days and 65% of the patients had reversible Grade 3 or greater hepatic toxicity. *(9)* In a recent report from the Netherlands, eight patients with uveal melanoma confined to the liver underwent isolated hepatic perfusion with 200 mg of melphalan given over 1 hour. Four patients responded, but three experienced Grade 3-4 transient hepatotoxicity. *(26)*

None of the available treatment options for metastatic uveal melanoma are satisfactory and more have emerged as standard of care for this disease. New approaches are urgently needed, and therapeutic strategies for this disease that also have activity in metastatic melanoma of non-ocular origin would be ideal.

Molecular Biology and Role of Raf-kinase in Uveal Melanoma

Both cutaneous and uveal melanomas exhibit complex patterns of chromosomal and molecular alterations, but despite their common embryological background, they display only partially overlapping abnormalities.

Alterations in chromosomes 1 and 6 are common to both cutaneous and uveal tumors, but alterations in chromosomes 3 and 8 are more characteristic of the ocular disease. *(27, 28)* Monosomy 3, trisomy 8, partial duplication of 8q or isochromosome 8q are detected in about 50% of uveal melanomas and are associated with ciliary body tumors. Chromosome 6 alterations are present in approximately 40% of cases and are more frequently associated with choroidal tumors. Abnormalities in chromosomes 3 and 8 often coexist, but changes in chromosome 3 and 6 alterations appear to be almost mutually exclusive. In addition, it appears that tumors with abnormalities of both chromosomes 3 and 8 are associated with a poor prognosis, while abnormalities of chromosome 6 are associated with a better prognosis. A rearrangement of chromosome 1 is identified in 25-30% of cases, most often in association with ciliary body melanomas, but its relationship to prognosis is less clear. *(3)* In any case, it remains unclear whether these gross chromosomal changes are associated with deregulation of specific genes.

Specific genes involved in the pathogenesis of uveal melanoma have not yet been identified. The expression of numerous proteins involved in cell-cycle regulation, apoptosis and the process of invasion and metastasis (including cyclin D1, p53, HDM2, c-myc, the matrix metalloproteinase [MMP] family, proteins involved in the TGF-β pathway, BCL-2, BRCA-2, p16, NF1 and telomerase) have been studied in uveal melanoma, but none of the studies offer conclusive results. *(3)* An interesting recent report of gene-expression profiling in 50 primary uveal melanomas showed that the tumors clustered into two classes that correlated strongly with risk of metastasis and that as few as three genes were required for accurate class prediction, but this was just a descriptive study that did not offer a pathogenic explanation to the findings. *(29)*

The RAS-MAPK signaling pathway is activated by a variety of receptor tyrosine kinases, such as the epidermal (EGFR), platelet-derived (PDGFR) or vascular-endothelial (VEGFR) growth factor receptors, and plays a central role in regulating the growth and survival of a broad spectrum of

human tumors. The RAF family of serine/threonine kinases (ARAF, B-RAF and RAF1) is at the center of this pathway and has been extensively studied as a promising target for anticancer therapy. *(30)*

B-RAF is the most frequently mutated oncogene in cutaneous melanoma. The most common mutation is a T to A transition at position 1796 in exon 15 of the B-RAF gene, resulting in a valine to leucine substitution at amino acid 599 in the B-RAF protein. When the V599E mutant was transfected into NIH3T3 cells, it had a 138-fold greater transforming efficiency than the wild-type B-RAF. *(31)* Studies in melanoma cell lines demonstrated that oncogenic B-RAF activates ERK signaling, induces proliferation and protects cells from apoptosis, lending strength to the fact that this oncogene has a central biologic role in the pathogenesis of cutaneous melanoma and is therefore an important therapeutic target. *(32)*

Although activation of the RAS-MAPK pathway has been noted in uveal melanoma several studies to date have failed to detect activating mutations in the B-RAF gene in this disease, in contrast to the findings in cutaneous melanoma. *(33 - 38)*

BAY 43-9006 (Sorafenib)

BAY 43-9006 (sorafenib) is a novel orally available bi-aryl urea initially purported to be a specific Raf1 kinase inhibitor, but more recently shown to target a number of receptor tyrosine kinases involved in angiogenesis and tumor progression, including wild type and V599E B-RAF, VEGF receptor (VEGFR)-2, VEGFR-3 and platelet-derived growth factor receptor beta (PDGFR-β). In addition, in vitro studies have shown that BAY 43-9006(sorafenib) inhibits ERK phosphorylation independent of which mutation caused aberrant activation of the RAS/MAPK pathway. *(39)*

In vitro incubation with BAY 43-9006 (sorafenib) results in growth inhibition of melanoma cell lines with an IC_{50} of less than 5μM. *(40)* In mice with transplanted human melanoma tumors, single-agent therapy with BAY 43-9006 (sorafenib) results in significant tumor growth delay compared to vehicle control. *(41)*

BAY 43-9006 (sorafenib) has been evaluated in combination with cytotoxic chemotherapy in a variety of solid tumor animal models. *(42)* Across many tumor types and with chemotherapies of several different classes, BAY 43-9006 (sorafenib) enhances the antitumor activity of chemotherapy. The most potent interaction was observed between BAY 43-9006 (sorafenib) and paclitaxel. The combination of BAY 43-9006 (sorafenib) and cisplatin was also significantly greater than cisplatin or BAY 43-9006 (sorafenib) alone. *(43)*

Experience with BAY 43-9006 (Sorafenib) in Non-uveal Melanoma

To date BAY 43-9006 (sorafenib) has not been evaluated, alone or in combination, in metastatic uveal melanoma, although, more than 1,000 cancer patients have received BAY 43-9006(sorafenib). Chronic administration is well-tolerated. The most commonly observed toxicities are macular rash and hand-foot syndrome, which generally do not require discontinuation of the drug. Fatigue, anorexia and diarrhea have also been observed in a minority of patients.

A Phase II trial of BAY 43-9006 (sorafenib) has been conducted among 37 patients with metastatic melanoma. *(44)* Patients were required to have progressive disease at the time of study entry as well as measurable disease. Using a randomized discontinuation design, patients were treated with BAY 43-9006 (sorafenib) in an open-label fashion for three months prior to response assessment. Patients with a 25% reduction in the sum of the cross-products of measurable lesions were eligible to continue open-label BAY 43-9006(sorafenib). Patients with a 25% increase in this same parameter or the interval development of new metastatic sites were discontinued. Patients with less than 25% reduction or increase and no evidence of new metastases were randomized to continue BAY 43-9006 (sorafenib) or begin placebo. Patients were followed at six-week intervals and were permitted to resume open-label BAY 43-9006 (sorafenib) if they progressed while on placebo.

Among 37 evaluable melanoma patients, one patient experienced a response, using this modified criteria. Six patients experienced stable disease at three-months, with most patients remaining stable beyond six months. Four of these six stable-disease patients were treated at the University of Pennsylvania. Three of these patients developed well demarcated areas of central tumor necrosis during the course of treatment. PET scans performed after four weeks of therapy demonstrated an absence of F-18 fluorodeoxyglucose (FDG) uptake in the core of these same tumors, supporting the observation that the central portion of these tumors was no longer viable. Two of these three patients had been randomized to placebo, progressed and maintained stable disease after resuming BAY 43-9006(sorafenib). Two of these patients remain on-study with stable disease for more than 12 months.

A Phase I/II trial combining BAY 43-9006(sorafenib), carboplatin and paclitaxel has been conducted among 47 patients with metastatic cutaneous melanoma. *(45)* Patients were required to have an Eastern Cooperative Oncology Group (ECOG) performance status of 0 or 1, progressive measurable disease, no more than three prior chemotherapy-containing regimens for metastatic disease, adequate organ function and no evidence of brain metastases. More than three quarters of the patients treated on the BAY 43-9006(sorafenib), carboplatin and paclitaxel trial had failed prior therapies. Carboplatin and paclitaxel were administered at an AUC of 6 and 225 mg/m^2, respectively, every three weeks. BAY 43-9006 (sorafenib) was given at 400 mg PO BID from Day 2 to Day 19 of each 21-day cycle. Patients who maintained a partial response or stable disease for six months were eligible to discontinue carboplatin and paclitaxel and remain on BAY 43-9006 (sorafenib) alone. Patients were evaluated for response every two cycles.

Myelosuppression was the most frequently observed severe toxicity. Nearly all patients required a reduction in the dose of carboplatin and paclitaxel by the end of four cycles due to prolonged Grade 4 neutropenia or prolonged Grade 3 thrombocytopenia. Thus, patients who remained on study for more than six months had many more cycles of chemotherapy administered at reduced doses than at the starting dose. The doses of both drugs were reduced by 25% when a dose reduction was first required. Those who continued to experience severe myelosuppression had their doses reduced to 50% of the starting doses. Two patients died of neutropenic sepsis during the second cycle. One patient withdrew during the first cycle. In a recent randomized Phase III clinical trial among patients with advanced non-small cell lung cancer (NSCLC), the toxic death rate was 4% among 200 patients treated with carboplatin and paclitaxel at the same doses studied in this trial. *(46)* Neutropenic fever was not observed in any of the patients who received chemotherapy at the 25% reduced dose. Cumulative neuropathy was limited, with three patients having Grade 2 neuropathy. In the previously cited NSCLC trial, the rate of Grade 3 neuropathy was 10%. Thus, the severe toxicities observed in this trial were in line with the reported experience with this chemotherapy regimen.

Sixteen patients had partial responses using RECIST criteria, of which fifteen are ongoing. Nine patients had evidence of minor regression and continue to receive treatment. Ten patients progressed after one to five months and two progressed after two cycles. The median time to progression has not been reached with a median follow-up of six months. Among the 37 patients with at least 3 months of follow-up, 27 (73%) remain on study with response or stable disease. The responses have been durable thus far, with only one responding patient having progression ten months after study entry and three months after discontinuing chemotherapy. The response rate and time to progression for this cohort exceeds the experience with even the most promising agents evaluated as first-line therapies for metastatic melanoma in large clinical trials. The size of this cohort is sufficient to demonstrate that the true response rate is greater than 25% (P < 0.05). This trial remains open to accrual. To date, over 60 patients with metastatic melanoma have been accrued.

Based on these results, ECOG is conducting a randomized, placebo-controlled Phase III trial of carboplatin, paclitaxel and BAY 43-9006 (sorafenib) in patients with unresectable locally advanced or metastatic cutaneous, mucosal and unknown primary melanomas. However, patients with metastatic uveal melanoma are excluded from this trial. Therefore, we believe it is

appropriate to evaluate this promising regimen in patients with metastatic uveal melanoma who are chemotherapy naïve or have received no more than one prior systemic chemotherapy regimen.

Correlative Studies

As described above, BAY 43-9006 (sorafenib) has been shown to target the RAF/MEK/ERK pathway, as well as receptor tyrosine kinases involved in angiogenesis and tumor progression, including VEGFR-2, VEGFR-3, PDGFβ, Flt-3 and c-KIT. *(39)* We propose to study a number of molecular markers in baseline tumor biopsies as well as in pre- and post-treatment blood and urine samples, to explore whether there is a correlation between these markers and response to treatment with this regimen in patients with metastatic uveal melanoma. We will collect a urine sample and 15 mL of peripheral blood in EDTA-containing tubes prior to treatment and on Day 17 (± 2 days; i.e. may be obtained between Days 15 and 19) between 11:00 am and 1:00 pm. Due to circadian variability of MAPK activation in peripheral blood lymphocytes, the time point of blood sampling is critical. MAPK activity will be measured as described by Chow et al. *(47)* Refer to Section 15.0 for details.

Microvessel density in tumor biopsies

One of the mechanisms by which BAY 43-9006 (sorafenib) exerts its antitumor effect is through an antiangiogenic effect. Microvessel density is a commonly used technique to quantify intra-tumoral angiogenesis and has been associated with patient outcome in several cancers. *(48)* We propose to measure microvessel density (MVD) in tumor biopsies in patients enrolled in this study. We hypothesize that treatment with BAY 43-9006 (sorafenib), paclitaxel and carboplatin will be more effective in tumors with a high baseline MVD.

Microvessel density will be determined after staining the sections with an antibody against CD34, a marker of blood vessels. The number of vessels will be counted in five representative 1 mm^2 sections. The AngioSys software from TCS Cellworks (Buckingham UK) will be used to automate analysis of MVD.

ERK 1/2 phosphorylation in PMA-stimulated lymphocytes

In a Phase I trial of BAY 43-9006 (sorafenib), a decrease in ERK 1/2 phosphorylation measured by flow cytometry in patients' PMA (phorbol myristate acetate)-stimulated lymphocytes was used as a pharmacodynamic marker of activity of BAY 43-9006 (sorafenib). *(49, 50)* We propose to extend these studies in the context of a Phase II study and study the effect on ERK 1/2 phosphorylation in tumor samples. We hypothesize that ERK1/2 phosphorylation will be consistently decreased in patients' PMA stimulated lymphocytes.

VEGF levels in plasma and urine

The key factor involved in signaling for angiogenesis in nearly all human tumors is vascular endothelial growth factor (VEGF). *(51, 52)* VEGF is a critical growth factor necessary for blood vessel formation. Elevated levels of plasma VEGF have been shown to correlate with decreased survival in many types of malignancy. *(53)* VEGF is known to function as a paracrine and as an autocrine factor in some tumors. *(54)* Since BAY 43-9006 (sorafenib) has antiangiogenic

properties, as described above, we hypothesize that the tumors growing in high VEGF conditions will be more sensitive to this drug and that therefore, patients with elevated levels of VEGF in plasma or urine will be more likely to respond to the combination that is being tested.

We will use an ELISA assay to monitor plasma and urine levels of VEGF in patients before and after therapy with carboplatin, paclitaxel and BAY 43-9006 (sorafenib). A series of standards of known VEGF concentration dissolved in VEGF-depleted plasma will be included in the analysis to allow the actual pg/ml concentration of VEGF in patient samples to be determined.

Hypothesis

There is no standard therapy for unresectable or Stage IV uveal melanoma. Although there are some differences in the clinical behavior and molecular biology between uveal and cutaneous melanomas, they do retain some commonality. Given the promising results obtained with the combination of carboplatin, paclitaxel and BAY 43-9006 (sorafenib) in patients with cutaneous melanomas, this combination should be tested in the setting of a Phase II clinical trial in patients with metastatic uveal melanoma.

Inclusion of Women and Minorities:

This study was designed to include women and minorities, but was not designed to measure differences of intervention effects.

3.0 DRUG INFORMATION

3.1 BAY 43-9006 (sorafenib; NSC-724772) (IND-69896)

a. DESCRIPTION

Chemical Name: 4-{4-[3-(4-chloro-3-trifluoromethyl-phenyl)ureido]-phenoxy}-pyridine-2 carboxylic acid methylamide-4-methylbenzensulfonate

Other Names: BAY 54-9085 is the tosylate salt of BAY 43-9006; sorafenib

Classification: Kinase inhibitor (Raf, VEGF-R and PDGF-R)

Mechanism of Action: The ras/raf signaling pathway is an important mediator of responses to growth signals and angiogenic factors. This pathway is often aberrantly activated in human tumors due to presence of activated ras, mutant b-raf, or over expression of growth factor receptors.

BAY 43-9006 (sorafenib) is a potent inhibitor of c-raf, and wild-type and mutant b-raf in vitro. Additionally, further characterization of BAY 43-9006 (sorafenib) tosylate revealed that this agent inhibits several receptor tyrosine kinases (RTKs) that are involved in tumor progression (VEGF-R, PDGF-R, Flt3, and c-KIT) and p38α, a member of the MAPK family.

Molecular Formula: $C_{12}H_{16}ClF_3N_4O_3$ X $C_7H_8O_3S$

Molecular Weight: BAY 43-9006 tosylate (sorafenib): 637 Daltons; BAY 43-9006 (sorafenib) (free base): 465 Daltons

Approximate Solubility: 0.19 mg/100 mL in 0.1 N HCl, 453 mg/100 mL in ethanol, and 2971 mg/100 mL in PEG 400.

b. TOXICOLOGY

Comprehensive Adverse Events and Potential Risks List (CAEPR) for Sorafenib (BAY 43-9006, NSC 724772)

The Comprehensive Adverse Event and Potential Risks list (CAEPR) provides a single list of reported and/or potential adverse events (AE) associated with an agent using a uniform presentation of events by body system. In addition to the comprehensive list, a subset, the Agent Specific Adverse Event List (ASAEL), appears in a separate column and is identified with ***bold*** and ***italicized*** text. This subset of AEs (ASAEL) contains events that are considered 'expected' for expedited reporting purposes only. Refer to the 'CTEP, NCI Guidelines: Adverse Event Reporting Requirements' http://ctep.cancer.gov/reporting/adeers.html for further clarification. *Frequency is provided based on 1376 patients*. Below is the CAEPR for sorafenib.

Version 2.1, June 23, 2006[1]

**Adverse Events with Possible Relationship to Sorafenib (BAY 43-9006) (CTCAE v3.0 Term) [n=1376 patients]**			**'Agent Specific Adverse Event List' (ASAEL)**
**Likely (>20%)**	**Less Likely (<=20%)**	**Rare but Serious (<3%)**	
ALLERGY/IMMUNOLOGY			
	Allergic reaction/hypersensitivity (including drug fever)		
BLOOD/BONE MARROW			
	Hemoglobin		
	Leukocytes (total WBC)		
	Lymphopenia		
	Neutrophils/granulocytes (ANC/AGC)		
	Platelets		

CARDIAC GENERAL			
	Hypertension		***Hypertension***
CONSTITUTIONAL SYMPTOMS			
Fatigue (asthenia, lethargy, malaise)			***Fatigue (asthenia, lethargy, malaise)***
	Fever (in the absence of neutropenia, where neutropenia is defined as ANC <1.0 x 10e9/L)		
	Rigors/chills		
	Weight loss		
DERMATOLOGY/SKIN			
	Dermatology/Skin - Other (non-life threatening squamous cell carcinoma of skin: keratoacanthoma type)		
	Dermatology/Skin - Other (other)		
	Dry skin		
	Flushing		
	Hair loss/alopecia (scalp or body)		
	Hypopigmentation		
	Nail changes		
	Pruritus/itching		
Rash/desquamation			***Rash/desquamation***
Rash: hand-foot skin reaction			***Rash: hand-foot skin reaction***
GASTROINTESTINAL			
	Anorexia		
	Ascites (non-malignant)		
	Constipation		
	Dehydration		
Diarrhea			***Diarrhea***
	Dysphagia (difficulty swallowing)		
	Flatulence		
	Heartburn/dyspepsia		
	Mucositis/stomatitis (functional/symptomatic): pharynx		
	Nausea		
		Perforation, GI - NOS	
	Vomiting		
HEMORRHAGE/BLEEDING			
	Hemorrhage GI - Select		
	Hemorrhage GU - Select		
HEPATOBILIARY/PANCREAS			
	Pancreatitis		

INFECTION			
	Febrile neutropenia (fever of unknown origin without clinically or microbiologically documented infection)(ANC <1.0 x 10e9/L, fever >=38.5 degrees C)		
	Infection with unknown ANC - Select		
METABOLIC/LABORATORY			
	Albumin, serum-low (hypoalbuminemia)		
	Alkaline phosphatase		
	ALT, SGPT (serum glutamic pyruvic transaminase)		
	Amylase		
	AST, SGOT (serum glutamic oxaloacetic transaminase)		
	Bilirubin (hyperbilirubinemia)		
	GGT (gamma-glutamyl transpeptidase)		
	Glucose, serum-high (hyperglycemia)		
	Lipase		
	Metabolic/Laboratory - Other (blood elastase)		
	Phosphate, serum-low (hypophosphatemia)		
NEUROLOGY			
	Neuropathy: sensory		
PAIN			
	Pain - abdomen NOS		
	Pain - joint		
	Pain - muscle		
	Pain NOS		
PULMONARY/UPPER RESPIRATORY			
	Hypoxia		
	Pleural effusion (non-malignant)		
	Pneumonitis/pulmonary infiltrates		
	Pneumothorax		
RENAL/GENITOURINARY			
	Renal failure		
SYNDROMES			
	Flu-like syndrome		

[1] This table will be updated as the toxicity profile of the agent is revised. Updates will be distributed to all Principal Investigators at the time of revision. The current version can be obtained by contacting ADEERSMD@tech-res.com. Your name, the name of the investigator, the protocol and the agent should be included in the e-mail.

Also reported on BAY 43-9006 trials but with the relationship to BAY 43-9006 still undetermined:
ALLERGY/IMMUNOLOGY - linear IgA disease
CARDIAC ARRHYTHMIA - atrial flutter; supraventricular arrhythmia
CARDIAC GENERAL - cardiac ischemia/infarction; left ventricular systolic dysfunction
COAGULATION - INR; PTT; thrombotic microangiopathy
DERMATOLOGY/SKIN - erythema multiforme
GASTROINTESTINAL - ileus
HEMORRHAGE/BLEEDING - CNS hemorrhage; petechiae; pleural hemorrhage; splenic infarction
LYMPHATICS - limb edema
METABOLIC/LABORATORY - creatinine; hyperuricemia; hyponatremia
MUSCULOSKELETAL/SOFT TISSUE - arthritis
NEUROLOGY - anxiety; CNS ischemia; dizziness; encephalopathy; memory impairment; psychosis; syncope
OCULAR/VISUAL - diplopia; uveitis
PAIN - back pain; bone pain; chest/thorax pain; headache; limb pain
PULMONARY/UPPER RESPIRATORY - ARDS; dyspnea; voice changes
SEXUAL/REPRODUCTIVE FUNCTION - erectile dysfunction
VASCULAR - thrombosis/thrombus/embolism

Note: BAY 43-9006 in combination with other agents could cause an exacerbation of any adverse event currently known to be caused by the other agent, or the combination may result in events never previously associated with either agent.

Potential Drug Interactions: BAY 43-9006 tosylate (sorafenib) is neither a clinically meaningful inhibitor nor a clinically meaningful inducer of CYP2C19, CYP2D6 and CYP3A4 isoenzymes and is not expected to significantly increase or decrease the exposure of co-administered compounds metabolized by these pathways.

However, concomitant administration of BAY 43-9006 tosylate (sorafenib) and CYP3A4 inducers, such as phenytoin, carbamazepine, Phenobarbital, rifampin or St. John's Wort should be avoided. Additionally, BAY 43-9006 tosylate (sorafenib) is 97% to 99% protein bound; no drug interactions have been reported in studies, thus far.

c. PHARMACOLOGY

How Supplied: BAY 43-9006 tosylate (sorafenib) is supplied as an immediate-release film-coated, round, and salmon color tablet containing 200 mg of the free base, BAY 43-9006, and the excipients croscarmellose sodium, microcrystalline cellulose, hydroxypropylmethylcellulose, sodium lauryl sulfate, and magnesium stearate. The film-coat consists of hydroxypropylmethyl cellulose, polyethylene glycol, tiranium dioxide and red iron oxide. The film coating has no effect on active BAY 43-9006 tosylate (sorafenib) release rate.

BAY 43-9006 tosylate (sorafenib) 200 mg tablets are supplied in bottles of 140 tablets.

Storage: Store at controlled room temperature (15ºC – 25ºC). Storage conditions should not exceed 25ºC.

Stability: Stability studies with the 200 mg dosage form are ongoing. The current shelf life is 24 months when stored at controlled room temperature.

Route of Administration: Oral.

Method of Administration: Food does not clearly affect BAY 43-9006 tosylate (sorafenib) pharmacokinetics. It may be taken without regard to meals and with at least 250 mL of water. However, if taken with meals, instruct patients to take BAY 43-9006 tosylate (sorafenib) with a moderate to low-fat meal. A high fat meal caused a 29% decrease in BAY 43-9006 (sorafenib) AUC and a 38% decrease in BAY 43-9006 (sorafenib) C_{max} value.

d. SUPPLIER

BAY 43-9006 (sorafenib) is an investigational agent supplied to investigators by the Division of Cancer Treatment and Diagnosis (DCTD), NCI. BAY 43-9006 (sorafenib) is provided to the NCI under a Clinical Trials Agreement between Bayer Pharmaceuticals, Onyx Pharmaceuticals and the NCI Division of Cancer Treatment and Diagnostic (DCTD).

Drug Ordering: BAY 43-9006 (sorafenib) may be requested by the Principal Investigator (or their authorized designee) at each participating institution. Pharmaceutical Management Branch (PMB) policy requires that drug be shipped directly to the institution where the patient is to be treated. PMB does not permit the transfer of agents between institutions (unless prior approval from the PMB is obtained). The CTEP assigned protocol number (**S0512**) must be used for ordering all CTEP supplied investigational agents. The responsible investigator at each participating institution must be registered with CTEP, DCTD, through an annual submission of FDA form 1572, Supplemental Investigator Data Form (IDF), Financial Disclosure Form (FDF) and a Curriculum Vitae (CV). If there are several participating investigators at one institution, CTEP supplied investigational agents for the study should be ordered under the name of one lead investigator at that institution.

Drug may be requested by completing a Clinical Drug Request (NIH-986) form and mailing it to the Pharmaceutical Management Branch, CTEP, DCTD, NCI, 9900 Rockville Pike, EPN Room 7149, Bethesda, MD 20892 or faxing it to 301/480-4612. For questions call 301/496-5725.

Drug Returns: All unused drug supplies should be returned to the PMB. When it is necessary to return study drug (e.g., sealed vials remaining when expired vials are recalled by the PMB), investigators should return the study drug to the PMB using the NCI Return Agent Form available on the NCI home page (http://ctep.cancer.gov) or by calling the PMB at 301/496-5725.

Drug Accountability: The investigator, or a responsible party designated by the investigator, must maintain a careful record of the receipt, disposition, and return of all drugs received from the PMB using the Drug Accountability Record Form available on the NCI home page (http://ctep.cancer.gov) or by calling the PMB at 301/496-5725.

Questions about drug orders, transfers, returns or accountability should be addressed to the PMB by calling 301/496-5725 Monday through Friday between 8:30 am and 4:30 pm Eastern Time.

3.2 Carboplatin (CBDCA) (NSC-241240)

a. DESCRIPTION

Carboplatin (CBDCA) is a hydrophilic platinum coordination compound and is an analog of cisplatin, producing intrastrand DNA cross-links.

b. TOXICOLOGY

Human Toxicology: Side effects of carboplatin (CBDCA) include myelosuppression, nausea, vomiting, abdominal pain, diarrhea and constipation. Other toxicities include allergic reaction (including hypersensitivity, i.e., rash, urticaria, erythema, pruritus, bronchospasm and hypotension), peripheral neuropathy, paresthesia, loss of hair, hearing loss, visual disturbances and change in taste. Serum creatinine elevations and blood urea elevations have occurred as well as abnormal liver function tests and decreased serum electrolyte values. Although rare, pain, asthenia, cardiovascular, respiratory, genitourinary and mucosal side effects have occurred in some patients. Cancer-associated hemolytic uremic syndrome has been reported rarely. The renal effects of nephrotoxic compounds may be potentiated by carboplatin. Carboplatin is contraindicated in patients with a history of severe allergic reactions to cisplatin or other platinum-containing compounds or mannitol. This drug should not be used in patients with severe bone marrow depression or significant bleeding. The occurrence of acute leukemia has been reported rarely in patients treated with anthracycline/alkylator combination chemotherapy.

Pregnancy and Lactation: Carboplatin may cause fetal harm, therefore women of childbearing potential should be advised to avoid becoming pregnant.

Refer to the FDA-approved package insert for comprehensive toxicity information and preparation instructions.

c. PHARMACOLOGY

Kinetics: The differences in potencies of carboplatin and cisplatin are due to differences in aquation rates. The initial half-life is 1.1 - 2.0 hours and the post-distributional half-life is 2.6 - 5.9 hours. Sixty-five percent of the dose is excreted in the urine within twelve hours. Carboplatin is not bound to plasma proteins.

Formulation: Carboplatin is supplied as a sterile lyophilized powder available in single-dose vials containing 50 mg, 150 mg and 450 mg of carboplatin for administration by intravenous injection. Each vial contains equal parts by weight of carboplatin and mannitol. Immediately before use, the content of each vial must be reconstituted with either Sterile Water for Injection, USP, 5% Dextrose in Water, or 0.9% Sodium Chloride Injection, USP, according to the following schedule:

Vial Strength	Diluent Volume
50 mg	5 ml
150 mg	15 ml
450 mg	45 ml

These dilutions all produce a carboplatin concentration of 10 mg/mL. Carboplatin can be further diluted to concentrations as low as 0.5 mg/mL with 5% Dextrose in Water or 0.9% Sodium Chloride Injection, USP (NS).

Storage and Stability: Unopened vials of carboplatin for injection are stable for the life indicated on the package when stored at controlled room temperature 15° - 30°C, and protected from light. When reconstituted as directed, the solution of carboplatin exhibits no decomposition for 8 hours at room temperature (25°C). Like cisplatin, this drug should not be given through aluminum needles. **CAUTION:** The single-use lyophilized dosage form contains no antibacterial preservatives. Therefore, it is advised that the reconstituted product be discarded eight hours after dilution.

Administration: Intravenous.

d. SUPPLIER

Supplier: Carboplatin is commercially available for purchase by the third party. This drug will not be supplied by the NCI.

3.3 Paclitaxel, Taxol® (NSC-673089)

a. DESCRIPTION

Chemistry: Paclitaxel is a diterpene plant product found in the needles and bark of the western yew, Taxus brevifolia. The marketed formulation is prepared in a semi-synthetic process.

Molecular Weight: 853.9

Empirical Formula: $C_{47}H_{51}NO_{14}$

Description: Clear viscous fluid

b. TOXICOLOGY

Human Toxicity:

Dose-limiting toxicity is myelosuppression with reversible granulocytopenia, anemia, and thrombocytopenia. Allergic reactions occur in up to 8% of patients receiving paclitaxel as an intravenous infusion over 6 to 24 hours. These can be acute anaphylactoid reactions to include flushing, hypotension, and bronchospasm; dermatitis and pruritus are also observed. Hypertension has also been seen, and may be related to concomitant medication with dexamethasone. Premedication with diphenhydramine, cimetidine, and dexamethasone appears to diminish the incidence of these reactions. Neurotoxicity can include distal painful paresthesias. Rarely, this toxicity has required discontinuation of drug due to pain, impairment of fine motor skills, or difficulty ambulating. Experience to date suggests that this neuropathy is reversible. Rarely, associated forms of neurotoxicity have included taste perversion, seizures, and mood changes. Some patients have reported vision abnormalities such as blurred vision, "flashing lights" and scintillating scotomata. Ischemic or infarcted colon, sometimes with involvement of other parts of the gastrointestinal tract, has also been seen. Patients reporting abdominal discomfort should be monitored closely. These events generally occurred while the patients were severely neutropenic. They may be most consistent with neutropenic enterocolitis (typhlitis). Although increased SGOT, SGPT, bilirubin and alkaline phosphatase,

as well as hepatic failure and hepatic necrosis have been seen, one patient receiving this drug has also experienced hepatic encephalopathy, and two incidences of pancreatitis have been noted. Neuroencephalopathy has also been reported. Pulmonary toxicities that have occurred are pneumonitis and radiation pneumonitis (following concomitant paclitaxel and radiation).

Other non-hematologic reactions include: diarrhea, alopecia, myalgias and arthralgias, nausea or vomiting, mucositis (stomatitis and pharyngitis), light-headedness, myopathy and fatigue. Less commonly, cardiotoxicity has been associated with paclitaxel administration, to include arrhythmias (sinus bradycardia, ventricular tachycardia, atrial arrhythmia, and heart block), and myocardial infarction. Skin reactions including erythema, induration, tenderness, ulceration, radiation recall, rash and nail changes have occurred including discoloration of fingernails and separation from nail bed.

Pregnancy and Lactation: Paclitaxel may cause fetal harm when administered to a pregnant woman. Paclitaxel has been shown to be embryo- and fetotoxic in rats and rabbits and to decrease fertility in rats. In these studies, paclitaxel was shown to result in abortions, decreased corpora lutea, a decrease in implanatations and live fetuses, and increased resorption and embryo-fetal deaths. No information is available on the excretion of this drug in human milk. Because many drugs are excreted in human milk and because of the potential for serious adverse reactions in nursing infants, it is recommended that nursing be discontinued.

Refer to FDA-approved package insert for comprehensive toxicity information and preparation instructions.

c. PHARMACOLOGY

<u>Formulation</u>: Sterile solution containing 6 mg/ml in a 5 ml vial (30 mg per vial) in polyoxyethylated castor oil (Cremaphor EL) 50% and dehydrated alcohol, USP, 50%. There are also vial sizes of 100 mg and 300 mg.

<u>Solution Preparation</u>: Paclitaxel is reconstituted by diluting the total dose in 0.9% Sodium Chloride Injection, USP, or 5% Dextrose Injection, USP (D5W) to maintain a paclitaxel concentration between 0.3 and 1.2 mg/ml. Paclitaxel must be prepared in glass or polyolefin containers due to leaching of diethylhexlphthalate (DEHP) plasticizer from polyvinyl chloride (PVC) bags and intravenous tubing by the Cremaphor vehicle in which paclitaxel is solubilized. Each bag/bottle should be prepared immediately before administration.

NOTE: Formation of a small number of fibers in solution (within acceptable limits established by the USP Particulate Matter Test for LVPs) has been observed after preparation of paclitaxel. Therefore, in-line filtration is necessary for administration of paclitaxel solutions. In-line filtrations should be accomplished by incorporating a hydrophilic, microporous filter of pore size not greater than 0.22 microns (e.g., IVEX-II or IVEX-HP or equivalent) into the IV fluid pathway distal to the infusion pump. Although particulate formation does not indicate loss of drug potency, solutions exhibiting excessive particulate matter formation should not be used.

<u>Administration of Paclitaxel</u>: Paclitaxel, at the appropriate dose, will be given as an intravenous infusion as specified in the protocol, diluted in 0.9% Sodium Chloride Injection, USP, or 5% Dextrose Injection. Paclitaxel will be administered

via an infusion control device (pump) using non-PVC tubing and connectors, such as the IV administration sets (polyethylene or polyolefin) which are used to infuse parenteral nitroglycerin. Nothing else is to be infused through the line where paclitaxel is being administered.

Storage and stability: The intact vials of paclitaxel should be stored between 2 - 25°C. Based on stability data for Taxol® made from either natural or semi-synthetic paclitaxel, stored for up to 12 months at 40°C, potency losses were within the range of 2.0 to 2.4 percent per year. Samples stored for up to 3 months at 60°C lost potency at rates corresponding to 20 to 40% per year. Accordingly, vials left out in a warm place for a few days should still be satisfactory for use. All solutions of paclitaxel exhibit a slight haziness directly proportional to the concentration of drug and the time elapsed after preparation, although when prepared as described above, solutions of paclitaxel (0.3 - 1.2 mg/ml) are physically and chemically stable for 27 hours. Vials will be labeled with a firm expiration date.

d. SUPPLIER

Supplier: Paclitaxel is commercially available for purchase by the third party. This drug will not be supplied by the NCI.

4.0 STAGING CRITERIA

Patients will be eligible if they have advanced (Stage IV) uveal melanoma. All patients will be staged using the current version of the AJCC TNM staging criteria (Version 6.0).

**Stage IV**	Any T	Any N	M1

Regional Lymph Nodes (N)

NX	Regional lymph nodes cannot be assessed
N0	No regional lymph node metastasis
N1	Regional lymph node metastasis

Distant Metastasis (M)

MX	Distant metastasis cannot be assessed
M0	No distant metastasis
M1	Distant metastasis

5.0 ELIGIBILITY CRITERIA

Each of the criteria in the following section must be met in order for a patient to be considered eligible for registration. Use the spaces provided to confirm a patient's eligibility. For each criterion requiring test results and dates, please record this information on the Prestudy Form and submit to the Data Operations Center in Seattle (see Section 14.0). Any potential eligibility issues should be addressed to the Data Operations Center in Seattle at 206/652-2267 prior to registration.

SWOG Patient No. ______________________

Patient's Initials (L, F, M) ______________________________

____ 5.1 Patients must have histologically proven Stage IV metastatic uveal melanoma (see Section 4.0).

____ 5.2 Patients will be allowed up to one prior systemic regimen for Stage IV melanoma. Prior treatment must have been completed at least 28 days prior to registration. Prior systemic therapy is defined as a course of treatment during or after which a patient has demonstrated documented progression of disease. This includes patients who have received either of the following: a single chemotherapy agent (or regimen), a single immunotherapy agent (or regimen) or a single investigational treatment agent (or regimen).

____ 5.3 Patients must have measurable disease by RECIST criteria as outlined in Section 10.1. All measurable lesions must be assessed within 28 days prior to registration. Non-measurable disease must be assessed within 42 days prior to registration. Measurable disease within a previous radiation therapy port must demonstrate clearly progressive disease. All disease must be assessed and documented on the Baseline Tumor Assessment Form (Form #848).

____ 5.4 For patients with bone metastases, a baseline bone scan must be performed within 28 days prior to registration.

____ 5.5 Patients must have a Zubrod Performance Status of 0 or 1 (see Section 10.4).

____ 5.6 Patients must have adequate hematologic values documented by ANC > $1,500/mm^3$ and a platelet count > $100,000/mm^3$ obtained within 28 days prior to registration.

____ 5.7 Patients must have adequate renal function as documented by a serum creatinine ≤ 2 x the institutional upper limit of normal within 28 days prior to registration.

____ 5.8 Patients must have adequate hepatic function as documented by a serum bilirubin ≤ 2 x the institutional upper limit of normal and SGOT or SGPT ≤ 2 x the institutional upper limit of normal (or ≤ 5 x the institutional upper limit of normal, if hepatic metastasis is present) obtained within 28 days prior to registration.

____ 5.9 Patients on full-dose anticoagulants (e.g., warfarin) are eligible provided that both of the following criteria are met:

a) The patient has an in-range INR (usually between 2 and 3) obtained within 28 days prior to registration, and is on a stable dose of oral anticoagulant.

b) The patient has no active bleeding or pathological condition that carries a high risk of bleeding (e.g., tumor involving major vessels or known varices).

____ 5.10 Patients must not have any evidence of bleeding diathesis or active coagulopathy that carries a high risk of bleeding.

SWOG Patient No. ______________________

Patient's Initials (L, F, M) ______________________________

____ 5.11 Patients must not have hypertension with systolic blood pressure of > 140 mmHg or diastolic pressure > 90 mmHg at the time of registration. However, patients with well-controlled hypertension are eligible.

____ 5.12 Patients undergoing major surgery or a sustaining a significant traumatic injury within 21 days prior to treatment are ineligible.

____ 5.13 Patients must not have previously received BAY 43-9006 (sorafenib) or any other agents targeting raf or VEGF/VEGFR.

____ 5.14 Patients with any condition (e.g., gastrointestinal tract disease resulting in an inability to take oral medication or a requirement for IV alimentation, prior surgical procedures affecting absorption, or active peptic ulcer disease) that impairs their ability to swallow pills are not eligible.

____ 5.15 Patients must be 18 years of age or older.

____ 5.16 Patients must not be planning to receive systemic corticosteroid therapy. Topical and/or inhaled steroids are allowed.

____ 5.17 Patients are strongly encouraged to have their specimens submitted as outlined in Section 15.0.

____ 5.18 Patients must not be taking cytochrome P450 enzyme-inducing antiepileptic drugs (phenytoin, carbamazepine and Phenobarbital), rifampin or St. John's Wort.

____ 5.19 The effects of BAY 43-9006 (sorafenib) on the developing human fetus at the recommended therapeutic dose are unknown. For this reason and because raf kinase inhibitor agents are known to be teratogenic, patients must not be pregnant or nursing. Women/men of reproductive potential must have agreed to use an effective contraceptive method (hormonal or barrier method of birth control; abstinence) prior to study entry and for the duration of study participation. Should a woman become pregnant or suspect she is pregnant while participating in this study, she should inform her treating physician immediately.

____ 5.20 No prior malignancy is allowed except for the following: adequately treated basal cell or squamous cell skin cancer, in situ cervical cancer, adequately treated Stage I or II cancer from which the patient is currently in complete remission, or any other cancer from which the patient has been disease-free for five years.

____ 5.21 Patients must be informed of the investigational nature of this study and must sign and give written informed consent in accordance with institutional and federal guidelines.

5.22 If Day 21, 28 or 42 falls on a weekend or holiday, the limit may be extended to the next working day.

In calculating days of tests and measurements, the day a test or measurement is done is considered Day 0. Therefore, if a test is done on a Monday, the Monday four weeks later would be considered Day 28. This allows for efficient patient scheduling without exceeding the guidelines.

SWOG Patient No. ______________________

Patient's Initials (L, F, M) ______________________________

____ 5.23 At the time of patient registration, the treating institution's name and ID number must be provided to the Data Operations Center in Seattle in order to ensure that the current (within 365 days) date of institutional review board approval for this study has been entered into the data base.

6.0 STRATIFICATION FACTORS

This section is not applicable to this study.

7.0 TREATMENT PLAN

For treatment or dose modification related questions, please contact Dr. Aparicio at 713/563-6969 or Dr. Weber at 323/865-3962. For dosing principles or questions, please consult the Southwest Oncology Group Policy #38 "Dosing Principles for Patients on Clinical Trials" at http://swog.org (then click on "Policies and Manuals" under the "Visitors" menu and choose Policy 38).

7.1 Good Medical Practice

The following pre-study tests should be obtained within 28 days prior to registration in accordance with good medical practice. Results of these tests do not determine eligibility and minor deviations would be acceptable if they do not impact on patient safety in the clinical judgment of the treating physician. **The Study Coordinator must be contacted if there are significant deviations in the values of these tests.**

a. Electrolytes: Potassium, sodium, chloride, CO_2, calcium and glucose

b. LDH. If LDH is elevated, no need to contact Study Coordinator(s).

c. BAY 43-9006 (sorafenib) has the ability to inhibit a variety of liver metabolic enzymes in vitro. The clinical impact of this inhibition in humans taking drugs metabolized by these enzymes is unknown. Patients who are stable on any of the agents listed in Appendix 19.3 should also be monitored closely for the possibility of drug interactions that may enhance the toxicities of the study agent.

d. Patients taking narrow therapeutic index medications, including warfarin (Coumadin), quinidine, or digoxin should be monitored proactively (this includes prophylactic anticoagulation of venous or arterial access devices). PT/INR or drug levels should be monitored prior to initiation of BAY 43-9006 (sorafenib).

e. Except for cancer related abnormalities, patients should not have unstable or pre-existing major medical conditions that may affect the tolerance of cancer therapy.

f. Patients with known brain metastases should not participate on this clinical trial because of their poor prognosis and because they often develop progressive neurologic dysfunction that would confound the evaluation of neurologic and other adverse events.

g. Patients should not have psychological, familial, sociological or geographical conditions that prevent medical follow-up and compliance with the protocol treatment.

h. Patients should not have a significant history of cardiac disease, e.g., uncontrolled hypertension, unstable angina, congestive-heart failure, and myocardial infarction within the last six months, or cardiac ventricular arrhythmias requiring medication.

7.2 Treatment

AGENT	DOSE	ROUTE	DAYS	INTERVAL***
Carboplatin	AUC 6	IV	1	21 days
Paclitaxel	225 mg/m^2	IV	1	21 days
BAY 43-9006 (sorafenib)	400 mg BID* (two 200 mg tablets BID, total daily dose 800 mg)	Oral	2-19**	21 days

NOTE: A cycle is defined as 21 days.

* Patients are to swallow two 200 mg tablets whole with approximately 250 ml (8 oz.) of water, each morning and evening (i.e., q 12 hours). Tablets may be taken without food or with a low or moderate fat meal. Total maximum dose is 800 mg per day (2 doses of 400 mg each).

** BAY 43-9006 (sorafenib) will begin on Day 2 of each cycle and will be discontinued after the evening dose 3 days prior to the subsequent chemotherapy administration (i.e., Day 19 provided that chemotherapy is administered on the intended 21-day schedule). If chemotherapy is delayed beyond Day 21, BAY 43-9006 (sorafenib) should be continued until 3 days prior to the planned date of chemotherapy. If BAY 43-9006 is discontinued prior to Cycle 6, carboplatin and paclitaxel may be continued up to 6 cycles of therapy or more, if there is evidence of continuing response (please refer to Section 7.4c for details regarding the duration of chemotherapy.

*** If carboplatin and paclitaxel are discontinued, BAY 43-9006 (sorafenib) will be given daily on Days 1 - 21 of each cycle, without interruption, until one of the criteria in Section 7.7 is met.

With initiation of BAY 43-9006 (sorafenib) therapy, patients must have their blood pressure monitored (at home or by a physician or nurse) at least weekly until stable or at least weekly for the first 4 weeks of protocol treatment (see Section 8.3b). Results of the blood pressure readings must be reported back to the treating physician at the subsequent visit. However, the treating physician should be notified if the patient has a value of > 150/100 mmHg.

7.3 a. Carboplatin and Paclitaxel

Prophylactic antiemetic therapy will be given as carboplatin and paclitaxel have moderate emetogenic potential. Patients should receive intravenous ondansetron, granisetron, dolasetron, or aprepitant. In order to reduce the risk of hypersensitivity reactions, patients will take dexamethasone 12 hours and 6 hours prior to the scheduled administration of chemotherapy. An additional dose of dexamethasone, along with a single dose of diphenhydramine, will be administered prior to the administration of paclitaxel. The following regimen is recommended:

AGENT	ROUTE OF ADMINISTRATION	SCHEDULE
Ondansetron, granisetron, dolasetron or aprepitant	IV	Prior to infusion of paclitaxel and carboplatin on Day 1
Dexamethasone	PO	12 hours and 6 hours prior to paclitaxel and carboplatin on Day 1
Dexamethasone	IV or PO	Prior to infusion of paclitaxel and carboplatin on Day 1
Diphenhydramine	IV or PO	Prior to infusion of paclitaxel and carboplatin on Day 1

b. The dose of carboplatin will be calculated according to the following formula:

Carboplatin dose (mg) = AUC x [creatinine clearance (cc/min) + 25]

Creatinine clearance will be calculated using the Cockroft-Gault formula *(36):*

$$\text{Creatinine clearance (ml/min)} = \frac{(140 - \text{age}) \times \text{WT (kg)} \times 0.85 \text{ (if female)}}{72 \times \text{creatinine (mg/dl)}}$$

The serum creatinine used to calculate the carboplatin dose must be performed within three days prior to each cycle administration.

See Appendix 19.4 for Carboplatin Dosing Worksheet.

7.4 Duration of Therapy

a. Treatment cycles will be repeated every 21 days.

b. Patients will be evaluated after every 2 cycles (6 weeks) of treatment, while they are treated with chemotherapy and for the first two cycles of therapy in which they receive single agent BAY 43-9006. Thereafter, while on single agent BAY 43-9006 (sorafenib), disease evaluation will take place every 3 cycles (9 weeks) of treatment.

c. Carboplatin and paclitaxel will be continued for 6 cycles. Thereafter, they can be continued if, in the opinion of the treating physician there is evidence of continued decrease in tumor burden that is attributable to the chemotherapy. Once chemotherapy is discontinued, patients will continue to take single agent BAY 43-9006 until one of the criteria in Section 7.7 is met.

7.5 Intake Calendar

BAY 43-9006 (sorafenib) drug compliance will be recorded by patients on the Intake Calendar (see Appendix 19.2). Institutional CRAs will review and ascertain patient adherence with protocol therapy.

7.6 **Because this study contains an investigational drug for which CTEP holds the IND, it falls under CTEP requirements for full reporting. This involves required submission of cycle-specific toxicity and dose information (see the S0512 Treatment Form [Form #656] and the S0512 Adverse Event Form [Form #14672]. For this study, a cycle is defined as 21 days.**

7.7 **Criteria for removal from protocol treatment:**

a. Progression of disease or symptomatic deterioration (as defined in Section 10.2).

b. Unacceptable toxicity due to BAY 43-9006 (sorafenib).

c. BAY 43-9006 (sorafenib) treatment delay ≥ 4 weeks due to any reason or > 2 weeks for hypertension.

d. The patient may withdraw from the study at any time for any reason.

7.8 All reasons for discontinuation of treatment must be documented in the Off Treatment Notice (Form #8756).

7.9 All patients will be followed for a maximum of 3 years from the date of registration.

8.0 TOXICITIES TO BE MONITORED AND DOSAGE MODIFICATIONS

8.1 This study will utilize the CTCAE (NCI Common Terminology Criteria for Adverse Events) Version 3.0 for toxicity and Serious Adverse Event reporting. A copy of the CTCAE Version 3.0 can be downloaded from the CTEP home page (http://ctep.cancer.gov). **All appropriate treatment areas should have access to a copy of the CTCAE Version 3.0.**

8.2 There will be no dose escalations during this study.

8.3 Dose Modifications for BAY 43-9006 (sorafenib)-Associated Toxicities

AGENT	DOSE LEVEL	DOSE
BAY 43-9006 (sorafenib)	Full Dose	400 mg bid (800 mg total daily dose)
	-1 Level	200 mg bid (400 mg total daily dose)
	-2 Level	200 mg once daily

a. Up to two dose reductions due to unacceptable toxicity per patient is allowed per the table above. If multiple toxicities are experienced, dose modifications will be based on the toxicity requiring the largest dose reduction. If toxicity occurs, the appropriate supportive care will be used to ameliorate signs and symptoms.

b. Management of Hypertension

Grade (CTCAE v3.0)	Antihypertensive Therapy	Blood Pressure Monitoring	BAY 43-9006 (sorafenib) Dose
Grade 2 (asymptomatic)	Initiate monotherapy (suggest dihydropyridine calcium-channel blocker)	Increase frequency and monitor (by health professional) every 2 days until stabilized.	No change
Grade 2 (symptomatic/ persistent)<br><br>**OR**<br><br>diastolic BP >110 mm Hg<br><br>**OR**<br><br>Grade 3	Add agent(s): Ca++ channel blocker (if not already used), K+ channel opener, beta-blocker, thiazide diuretic)	Increase frequency and monitor (by health professional) every 2 days until stabilized; continue q2d monitoring to stabilization after dosing restarted.	Hold BAY 43-9006 (sorafenib) until symptoms resolve to ≤ Grade 1 and diastolic BP ≤ 100 mm/Hg.*<br><br>Resume treatment at 1 dose level lower**
Grade 4			Remove from BAY 43-9006 (sorafenib) treatment
* Patients requiring a delay of > 2 weeks should be removed from BAY 43-9006 (sorafenib) treatment unless, in the treating physician's opinion, the patient may benefit from continued treatment.<br>** Patients requiring > 2 dose reductions should be removed from BAY 43-9006 (sorafenib) treatment.			

c. Hand-Foot Skin Reaction

Interrupt treatment for ≥ Grade 2 until resolution ≤ Grade 1. Once resolved, resume at previous dose if this was the first occurrence of Grade 2 toxicity. If the occurrence is ≥ Grade 3, or if this is not the first occurrence of Grade 2, reduce BAY 43-9006 (sorafenib) by one dose level. Therapeutic adjuncts may be utilized. These may include topical emollients (such as Aquaphor), topical corticosteroids, topical or systemic antihistamines, and pyridoxine at 50 to 150 mg PO daily. A short course of systemic steroids for severe symptoms, not to exceed 5 days of therapy, is allowed at the treating physicians discretion.

d. Rash

In the event of CTC Grade 3 or 4 pustular rash, secondarily infected rash, or a rash/skin condition intolerable to the patient due to pruritus, aesthetics, etc., BAY 43-9006 (sorafenib) may be discontinued until the rash resolves, improves to ≤ Grade 1 or is within patient tolerability. BAY 43-9006 (sorafenib) may then be resumed at the same dose or reduced as needed to maintain skin toxicity at or below Grade 1 or 2 and within patient tolerability.

e. Nausea and Vomiting

In patients who have emesis and are unable to retain BAY 43-9006 (sorafenib) for longer than 30 minutes, every attempt should be made to obtain control of nausea and vomiting. The dose of BAY 43-9006 (sorafenib) may be repeated once per dose if emesis occurs within 30 minutes of taking the tablet(s). Antiemetics should be used to control nausea and vomiting.

f. Diarrhea

Antidiarrheal medications, potentially including loperamide as described below, along with supplemental fluids and other supportive measures are recommended at the discretion of the investigator.

In the event of Grade 3 diarrhea, BAY 43-9006 (sorafenib) should be discontinued until the diarrhea resolves to ≤ Grade 1. For CTC Grade 3 diarrhea, patients will be instructed to begin taking loperamide. Loperamide should be taken in the following manner: 4 mg at the first onset of diarrhea, then 2 mg every 2 hours around the clock until diarrhea-free for at least 12 hours. Patients may take loperamide 4 mg every four hours during the night. Additional antidiarrheal measures may be used at the discretion of the treating investigator. Patients should be instructed to increase fluid intake to help maintain fluid and electrolyte balance during episodes of diarrhea.

BAY 43-9006 (sorafenib) may be restarted with a dose reduction if the investigator feels the diarrhea is clearly drug related and no other cause is identified. If, despite the dose reduction, Grade 3 diarrhea recurs, BAY 43-9006 (sorafenib) must be discontinued and the patient removed from protocol treatment.

g. SGOT/SGPT

In the event of Grade 3 toxicity, BAY 43-9006 (sorafenib) should be held for two weeks and then restarted at a dose reduction if transaminases have returned to baseline. In the event of any Grade 4 hepatic toxicity, lack of resolution of elevated transaminases after BAY 43-9006 (sorafenib) is held for two weeks, or recurrence of ≥ Grade 3 toxicity after restarting treatment, BAY 43-9006 (sorafenib) must be discontinued and the patient must be removed from protocol treatment.

h. Allergic Reaction/Hypersensitivity

In the event of Grade 1 or 2 toxicity, treatment with diphenhydramine (Benadryl) 25 - 50 mg PO QD or hydroxazine (Atarax) 25 - 50 mg PO QD is recommended. If the toxicity is Grade 3 or 4, BAY 43-9006 (sorafenib) must be discontinued.

i. Neurotoxicity

BAY 43-9006 (sorafenib) must be withheld until resolution to ≤ Grade 1 (up to 4 weeks per Section 7.7c) for neurologic toxicity Grade > 2 unless condition(s) are related to patient's cancer or some other pre-existing condition that, in the opinion of the treating investigator, was not brought on or exacerbated by treatment with BAY 43-9006 (sorafenib). Such conditions might include headache, anxiety, or tinnitus. Following resolution to ≤ Grade 1, BAY 43-9006 (sorafenib) should be resumed at a one dose level reduction.

j. Mucositis

BAY 43-9006 (sorafenib) will be withheld when the sum of regions of ulcerative mucositis (gingivitis and glossitis) exceeds 1.0 cm^2 or for > Grade 2 stomatitis. Following resolution of Grade 2 toxicity to Grade 0, BAY 43-9006 (sorafenib) should be resumed at full dose. Following resolution of Grade 3 or 4 mucositis, therapy will resume at one dose level reduction. Angular stomatitis (cheilitis) as an isolated event is not reason for treatment modification.

k. Hematologic and Non-Hematologic Toxicity (not addressed above)

The administration of BAY 43-9006 (sorafenib) must be interrupted for ≥ Grade 3 toxicity to allow the toxicity to resolve or decrease to ≤ Grade 2. Subsequent treatment should be resumed with a one level dose reduction.

When the drug is being held, re-assessment of toxicity should be performed twice weekly and more frequently if clinically indicated.

If a dose or doses are missed, the reason(s) and the number of doses not taken should be noted and recorded on the **S0512** Treatment Form (Form #656). Every effort should be made to manage possible toxicities so that a patient remains on protocol treatment.

l. GI Perforation (NOS)

In the event of a GI perforation, patients must be removed from protocol treatment.

8.4 Carboplatin and Paclitaxel

NOTE: If carboplatin and paclitaxel are delayed or discontinued, patients should continue to receive BAY 43-9006 (sorafenib).

AGENTS	DOSE LEVEL	DOSE
Carboplatin	Full Dose	AUC = 6
	-1 Level	AUC = 5
	-2 Level	AUC = 4
Paclitaxel	Full Dose	225 mg/m^2
	-1 Level	175 mg/m^2
	-2 Level	150 mg/m^2

a. The dose of carboplatin and paclitaxel will be reduced at the start of the next cycle in the event of:

- Grade 4 neutropenia lasting more than 7 days
- Grade 4 neutropenia with fever (100.5° Fahrenheit or higher)
- Grade 4 thrombocytopenia
- Lack of recovery of ANC or platelet count to re-treatment levels by Day 21 of each cycle
- Grade ≥ 2 neurotoxicity which has recovered to ≤ Grade 1 by Day 1 of the next cycle
- Any Grade 3 non-hematologic toxicity attributed to carboplatin or paclitaxel which has recovered to ≤ Grade 1 by Day 1 of the next cycle

b. All dose reductions of carboplatin and paclitaxel will be permanent.

c. Any Grade 4 non-hematologic toxicity attributable to either paclitaxel or carboplatin will result in discontinuation of either agent. Patients may continue either agent in combination with BAY 43-9006 (sorafenib), in the absence of the discontinued agent.

8.5 Supportive Care

Erythropoietin may be administered for anemia/fatigue at the investigators' discretion. However, prophylactic use of granulocyte/platelet colony stimulating factors is not permitted during the first cycle of treatment. For subsequent cycles, see Section 8.6 for G-CSF guidelines.

For patients receiving BAY 43-9006 who develop hand-foot reaction, treatment with topical emollients (such as Aquaphor) for symptom relief is permitted (see Section 8.3c). Topical and/or oral steroids or antihistamine agents may be used. Vitamin B-6 (pyridoxine; 50 - 150 mg orally each day) may also be used.

All supportive measures consistent with optimal patient care will be given throughout the study.

8.6 G-CSF (Amgen) has been licensed by the Food and Drug Administration for the prevention of chemotherapy induced neutropenia. In this study, G-CSF will not be administered to all patients to prevent neutropenia. However, it may be used for patients who develop Grade 3 - 4 neutropenia. For patients who experience Grade 3 or 4 neutropenia or develop neutropenic fever between cycles of chemotherapy, G-CSF may be added to all subsequent cycles of chemotherapy. G-CSF is commercially available and should be purchased through third party mechanisms. The NCI will not provide G-CSF for this study.

If G-CSF is used, it is recommended that it be used in the following manner:

If a patient develops neutropenia following chemotherapy, all dose modifications outlined in the protocol will be followed according to the original protocol. However, G-CSF will be added to all subsequent cycles of chemotherapy, unless there is clinical suspicion that the neutropenia was due to an unrelated medical condition and not due to the chemotherapy.

If the patient maintains an AGC of $\geq$ 1,000/mm^3 throughout the initial cycle of G-CSF supported chemotherapy, then the next cycle of chemotherapy may be increased back to the original dose level with the continued support of the G-CSF. **No dose escalations above the original dose level should be performed on patients taking G-CSF.**

G-CSF will be given at a dose of 5 µg/kg/d subcutaneously beginning 24 hours after completion of chemotherapy and continuing until the ANC exceeds 1,000/mm^3 on two successive determinations. Doses may be rounded off to the nearest vial size (300 mcg and 480 mcg vials). G-CSF should be discontinued at least 24 hours before the next chemotherapy dose. While the patient is receiving G-CSF, the CBC should be monitored at least twice a week (more frequently if clinically indicated).

The use of G-CSF must be documented in the "Notes" section of the S0512 Cycle-Specific Treatment Form (Form #656) or the S0512 Cycle-Specific Adverse Event Form (Form #14672). Any toxicities associated with G-CSF must also be documented on these forms.

8.7 For treatment or dose modification related questions, please contact Dr. Aparicio at 713/563-6969 or Dr. Weber at 323/865-3962.

8.8 Unexpected or fatal toxicities (including suspected reactions) must be reported to the Operations Office, to the Study Coordinator, to the IRB and the NCI. The procedure for reporting adverse reactions is outlined in Section 16.0.

9.0 STUDY CALENDAR

S0512, "Phase II Trial of BAY 43 9006 (Sorafenib; NSC-724772) in Combination with Carboplatin and Paclitaxel in Patients with Metastatic Uveal Melanoma"

		Cycle 1			Cycle 2			Cycle 3			Ω	ƒ	√
**REQUIRED STUDIES**	PRE STUDY	WK 1	WK 2	WK 3	WK 4	WK 5	WK 6	WK 7	WK 8	WK 9	WK 10	Follow-Up Prior to Progression	Follow-Up After Progression
**PHYSICAL**													
History and Physical Exam	X				X			X			X	X	X
Blood Pressure ∞	X	X	X	X	X			X					
Weight and Performance Status	X				X			X			X	X	X
Toxicity Notation ¥		X	X	X	X			X			X		
Baseline Abnormalities Form	X												
Review Intake Calendar					X			X			X		
**LABORATORY**													
CBC/Differential/Platelets	X	X	X	X	X			X			X		
Bilirubin	X				X			X			X		
SGOT or SGPT	X				X			X			X		
Serum Creatinine €	X				X			X			X		
**Suggested Laboratory §**													
LDH	X				X			X			X		
PT/INR ≠	X	X	X	X	X			X					X
Electrolytes (see Section 7.1)	X				X			X			X		
**Specimen Submission per Section 15.0 ∑**													
Archived tumor specimen	X												
Peripheral blood	X			X									
Urine	X			X									
**SCANS**													
X-rays/scans for disease assessment	X							X				X	X
Bone scan µ	X												
**TREATMENT**													
Carboplatin		X			X			X			X		
Paclitaxel		X			X			X			X		
BAY 43-9006 (sorafenib) ∆		X	X	X	X	X	X	X	X	X	X		

NOTE: Forms are found in Section 18.0. Forms submission guidelines may be found in Section 14.0.

∞ Blood pressure must be monitored (at home or by a physician or nurse) at least weekly for the first 4 weeks of protocol treatment. Results of the blood pressure readings must be reported back to the treating physician at the subsequent visit. However, the treating physician should be notified if the patient has a value of > 150/100 mmHg (see Sections 7.2 and 8.3b).

¥ Patients should be monitored for toxicity weekly during first cycle of therapy, then prior to each cycle or at more frequent intervals appropriate for the patient as judged by the treating physician.

§ Results of these tests do not determine eligibility but are suggested at prestudy for Good Medical Practice (see Section 7.1). These tests are required during treatment as scheduled to assess toxicity.

≠ Patients on therapeutic anticoagulation should have PT/INR monitored closely during therapy (e.g., weekly for the first month and weekly for a minimum of 2 weeks following discontinuation of the agent). Therapy should be held if the coagulation parameters are higher than the intended therapeutic range.

∆ Continuous oral daily dosing to begin on Day 2 of each cycle and discontinued after the second dose 3 days prior to the subsequent chemotherapy administration (i.e., Day 19 provided that chemotherapy is administered on the intended 21-day schedule). If chemotherapy is discontinued, BAY 43-9006 (sorafenib) will be taken continuously on Days 1 - 21 of each cycle (see Sections 7.2 and 8.4 for more details).

∑ With the patient's consent, 15 mL of peripheral blood in EDTA containing tubes and a sample of urine will be collected prior to treatment and on Day 17 of treatment (± 2 days; i.e., acceptable between Days 15 and 19) between 11:00 am and 1:00 pm as well as materials from the patient's initial diagnostic procedure (see Section 15.0).

Ω BAY 43-9006 (sorafenib) should continue until one of the criteria in Section 7.7 is met. Upon completion of 6 cycles of chemotherapy, carboplatin and paclitaxel will be discontinued.

ƒ Reassessment of all disease involvement must be performed by the same method as baseline until documented progression or symptomatic deterioration.
Disease assessments will continue every 6 weeks for the first 8 cycles of therapy (24 weeks). Thereafter while on single agent BAY 43-9006 (sorafenib) disease evaluation will take place every 3 cycles (9 weeks). Response should be confirmed by a second determination at least 4 weeks after a complete or partial response is noted.

√ After disease progression, follow-up will occur (with lab tests and scans performed at the discretion of the treating investigator) every 6 months for the first two years and then annually thereafter for up to 3 years after registration or until death.

µ Per Section 5.4, if the patient has bone metastases.

€ Serum creatinine used for carboplatin dosing calculation must be performed within 3 days prior to dose.

10.0 CRITERIA FOR EVALUATION AND ENDPOINT DEFINITIONS

10.1 Measurability of lesions

a. **Measurable disease:** Lesions that can be accurately measured in at least one dimension by 1) medical photograph (skin or oral lesion), palpation, plain x-ray, CT, MRI or other conventional technique with longest diameter 2 cm or greater in the axial plane (bone lesions not included), or 2) spiral CT with longest diameter 1 cm or greater. Ultrasound is suitable only for superficial disease (superficial palpable nodes, subcutaneous lesions, thyroid nodules).

Conventional CT and MRI should be performed with cuts of 10 mm or less in slice thickness contiguously. Spiral CT should be performed using a 5 mm contiguous reconstruction algorithm.

b. **Non-measurable disease:** All other lesions including lesions too small to be considered measurable, pleural or pericardial effusions, ascites, bone disease, inflammatory breast disease, leptomeningeal disease, lymphangitis, pulmonitis, abdominal masses not confirmed and followed by imaging techniques, cystic lesions or disease documented by indirect evidence only (e.g., by lab values), previously radiated lesions that have not progressed.

10.2 **Objective status at each evaluation:** Objective Status is to be recorded at each evaluation. All measurable lesions up to a maximum of 5 lesions per organ and 10 lesions in total, representative of all involved organs, should be identified as target lesions at baseline. All measurable lesions not identified as target lesions are non-target lesions and are included as non-measurable disease. Measurements must be provided for target measurable lesions, while presence or absence must be noted for non-target measurable and non-measurable disease.

a. **Complete Response (CR):** Complete disappearance of all measurable and non-measurable disease. No new lesions. No disease related symptoms. Normalization of markers and other abnormal lab values. All disease must be assessed using the same technique as baseline.

b. **Partial Response (PR):** Applies only to patients with at least one measurable lesion. Greater than or equal to 30% decrease under baseline of the sum of longest diameters of all target measurable lesions. No unequivocal progression of non-measurable disease. No new lesions. All target measurable lesions must be assessed using the same techniques as baseline.

c. **Stable:** Does not qualify for CR, PR, Progression or Symptomatic Deterioration. All target measurable lesions must be assessed using the same techniques as baseline.

d. **Progression:** One or more of the following must occur: 20% increase in the sum of longest diameters of target measurable lesions over smallest sum observed (over baseline if no decrease during therapy) using the same techniques as baseline. Unequivocal progression of non-measurable disease in the opinion of the treating physician (an explanation must be provided). Appearance of any new lesion/site. Death due to disease without prior documentation of progression and without symptomatic deterioration (see Section 10.2e).

e. **Symptomatic deterioration:** Global deterioration of health status requiring discontinuation of treatment without objective evidence of progression. Efforts should be made to obtain objective evidence of progression after discontinuation.

f. **Assessment inadequate, objective status unknown:** Progression or symptomatic deterioration has not been documented, and one or more target measurable lesions have not been assessed or inconsistent assessment methods were used.

g. Objective status notes:

1. Non-measurable and non-target measurable disease do not affect objective status except in determination of CR (must be absent--a patient who otherwise has a CR, but who has non-measurable or non-target measurable disease present or not assessed, will be classified as having a PR), and in determination of progression (if new sites of disease develop or if unequivocal progression occurs in the opinion of the treating physician).

2. An objective status of PR or stable cannot follow one of CR. Stable can follow PR only in the rare case that tumor increases too little to qualify as progression, but enough that a previously documented 30% decrease no longer holds.

3. In cases for which initial flare reaction is possible (hypercalcemia, increased bone pain, erythema of skin lesions), objective status is not progression unless either symptoms persist beyond 4 weeks or there is additional evidence of progression.

4. Lesions that appear to increase in size due to presence of necrotic tissue will not be considered to have progressed.

5. For bone disease documented on bone scan only, increased uptake does not constitute unequivocal progression.

6. Appearance or worsening of pleural effusions does not constitute unequivocal progression unless cytologically proven of neoplastic origin.

7. If CR determination depends on a lesion for which the status is unclear by the required tests, it is recommended the residual lesion be investigated with biopsy or fine needle aspirate.

10.3 **Best Response:** This is calculated from the sequence of objective statuses.

a. CR: Two or more objective statuses of CR a minimum of four weeks apart documented before progression or symptomatic deterioration.

b. PR: Two or more objective statuses of PR or better a minimum of four weeks apart documented before progression or symptomatic deterioration, but not qualifying as CR.

c. Unconfirmed CR: One objective status of CR documented before progression or symptomatic deterioration but not qualifying as CR or PR.

d. Unconfirmed PR: One objective status of PR documented before progression or symptomatic deterioration but not qualifying as CR, PR or unconfirmed CR.

e. Stable/no response: At least one objective status of stable/no response documented at least 6 weeks after registration and before progression or symptomatic deterioration, but not qualifying as anything else above.

f. Increasing disease: Objective status of progression or symptomatic deterioration within 12 weeks of registration, not qualifying as anything else above.

g. Inadequate assessment, response unknown: Progression or symptomatic deterioration greater than 12 weeks after registration and no other response category applies.

10.4 **Performance Status**: Patients will be graded according to the Zubrod Performance Status Scale.

**POINT**	**DESCRIPTION**
0	Fully active, able to carry on all pre-disease performance without restriction.
1	Restricted in physically strenuous activity but ambulatory and able to carry out work of a light or sedentary nature, e.g., light housework, office work.
2	Ambulatory and capable of self-care but unable to carry out any work activities; up and about more than 50% of waking hours.
3	Capable of limited self-care, confined to bed or chair more than 50% of waking hours.
4	Completely disabled; cannot carry on any self-care; totally confined to bed or chair.

10.5 **Progression-Free Survival:** From date of registration to date of first documentation of progression or symptomatic deterioration (as defined in Sections 10.2d and 10.2e) or death due to any cause. Patients last known to be alive and progression-free are censored at date of last contact.

10.6 **Time to Death:** From date of registration to date of death due to any cause. Patients last known to be alive are censored at date of last contact.

11.0 STATISTICAL CONSIDERATIONS

11.1 The expected accrual rate is 2 - 3 patients per month.

11.2 A two-stage design will be used for patient accrual. It is assumed that this combination will not be of further interest if the true response rate (confirmed, complete and partial responses) is less than 5%, and that a true response probability of 20% or more would be of considerable interest. Twenty patients will be entered initially. If zero responses are observed, the study will be permanently closed and the agent concluded to be inactive. If one or more responses are observed in the first 20 patients, an additional 20 patients will be accrued. Five or more responses out of 40 will be considered evidence that this agent warrants further study, provided other factors, such as adverse events and survival, are also favorable. This design has a significance level of 5% and a power of 92%.

11.3 Forty patients will be sufficient to estimate progression-free survival and overall survival at a specific time point (e.g., 4 months) to within ± 15% (95% confidence interval).

11.4 Forty patients will also be sufficient to estimate adverse event rates to within ± 15% (95% confidence interval). Any adverse event with at least 5% probability of occurring is likely to be seen at least once with 87% probability.

11.5 The analysis of the relationship between clinical outcomes and various tumor markers (baseline MVD and changes in VEGF in plasma and urine, as well as ERK 1/2 phosphorylation in peripheral blood mononuclear cells) will be exploratory and will not generate definitive results. However, they will provide the basis for the generation of hypotheses to be tested in larger patient cohorts.

11.6 There is no formal data and safety monitoring committee for Phase II studies. Toxicity and accrual monitoring are done routinely by the Study Coordinator, Study Statistician and the Disease Committee Chair. Response monitoring is done by the Study Statistician and Study Coordinator. Accrual reports are generated weekly and formal toxicity reports are generated every 6 months. In addition, the Statistical Center, Adverse Event Coordinator at the Operations Office, SAE Physician Reviewer and Study Coordinator monitor toxicities on an ongoing basis.

12.0 DISCIPLINE REVIEW

There will be no discipline review for this study.

13.0 REGISTRATION GUIDELINES

13.1 Patients must be registered prior to initiation of treatment (no more than three working days prior to planned start of treatment).

13.2 For either phone or web registration, the individual registering the patient must have completed the appropriate Southwest Oncology Group Registration Form. The completed form must be referred to during the registration but should not be submitted as part of the patient data.

The individual registering the patient must also be prepared to provide the treating institution's name and ID number in order to ensure that the current (within 365 days) date of institutional review board approval for this study has been entered into the data base. Patients will not be registered if the IRB approval date has not been provided or is > 365 days prior to the date of registration.

13.3 Registration procedures

a. You may register patients from Member, CCOP and approved Affiliate institutions to a therapeutics study using the SWOG Registration program. To access the Registration program go to the SWOG Web site (http://swog.org) and click on the *Logon* link to go to the SWOG Members Area logon page (https://swog.org/visitors/logon.asp). This Web program is available at any time except for periods listed **under *Down Times*.** Log on as an Individual User using your SWOG Roster ID Number and individual web user password. Help for the logon process may be found at https://swog.org/visitors/logonhelp.asp. After you have logged on, click on the *Clinical Trials* link and then the *Patient Reg* link to go to the Entry Page for the Patient Registration program. If you are a Registrar at an institution with Internet access you are encouraged to register this way. For new users, the link to a "Starter Kit" of help files may be found by clicking on **Starter Kit link at the logon page.**

To register a patient the following must be done (in order):

1. You are entered into the Southwest Oncology Group Roster and issued a SWOG Roster ID Number,

2. You are associated as an investigator or CRA/RN to the institution where a registration is occurring, and

3. You are granted permission to use the Patient Registration program at that institution.

For assistance with points 1 and 2 call the SWOG Operations Office at 210/677-8808. For point 3 you must contact your Web User Administrator. Each SWOG institution has one or more Web User Administrators who may set up Web Users at their institution and assign permissions and passwords to these users. For other password problems or problems with the Patient Registration program, please e-mail webreghelp@crab.org. Include your name, Roster ID Number, and telephone number, when the problem occurred, and exactly what you were doing.

b. If the Web Reg program is not used, the registration must be done by phone.

Member, Affiliate and CCOP Institutions

Registration by phone of patients from member, affiliate and CCOP institutions must be done through the Southwest Oncology Group Data Operations Center in Seattle by telephoning 206/652-2267, 6:30 a.m. to 4:00 p.m. Pacific Time, Monday through Friday, excluding holidays.

13.4 For either method of registration, exceptions to Southwest Oncology Group registration policies will not be permitted.

a. Patients must meet all eligibility requirements.

b. Institutions must be identified as approved for registration.

c. Registrations may not be cancelled.

d. Late registrations (after initiation of treatment) will not be accepted.

14.0 DATA SUBMISSION SCHEDULE

14.1 Data must be submitted according to the protocol requirements for **ALL** patients registered, whether or not assigned treatment is administered, including patients deemed to be ineligible. Patients for whom documentation is inadequate to determine eligibility will generally be deemed ineligible.

14.2 Master forms are included in Section 18.0 and (with the exception of the sample consent form and the Registration Form) must be photocopied for data submission to the Data Operations Center in Seattle. Alternatively, data from approved SWOG institutions may be submitted on-line via the Web; see Section 14.3a for details.

14.3 Data Submission Procedures. Please select **one** option for submitting specific data. Data submitted electronically or via facsimile should **not** be followed up with a mailed version.

a. Southwest Oncology Group Member Institutions, CCOPs and approved Affiliate institutions may submit data electronically via the Web by using the SWOG CRA Workbench. To access the CRA Workbench, go to the SWOG Web site (http://swog.org) and logon to the Members Area. After you have logged on, click on the *CRA Workbench* link to access the home page for CRA Workbench website. Next, click on the *Data Submission* link and follow the instructions. If

you are a CRA at an institution with Internet access, you are encouraged to submit data this way. For new users, the link to a "Starter Kit" of help files may be found by clicking on the **Starter Kit** link at the Members' logon page.

To submit data via the web the following must be done (in order):

1. You are entered into the Southwest Oncology Group Roster and issued a SWOG Roster ID Number,
2. You are associated as an investigator or CRA/RN at the institution where the patient is being treated or followed, and
3. Your Web User Administrator has added you as a web user and has given you the appropriate system permissions to submit data for that institution.

For assistance with points 1 and 2 call the Operations Office at 210/677-8808. For point 3, contact your local Web User Administrator (refer to the "Who is my Web User Administrator?" function on the swog.org Members logon page). For other difficulties with the CRA Workbench, please email *technicalquestion@crab.org*.

b. Alternatives to the CRA Workbench/web based data submission option are: submission via facsimile, surface, or express mail.

For facsimile submission: Member, CCOP and approved Affiliate institutions may submit data via facsimile to 800/892-4007 or 206/342-1680 locally. Please do not use cover sheet for faxed data. Facsimile submission is the 2nd preferred option for data submission.

For surface or express mail: Group Member Institutions, CCOPs and approved Affiliate institutions must submit one copy of all data forms directly to the Southwest Oncology Group Data Operations Center in Seattle at the address below. Affiliates must submit (number of copies to be determined by the Group member) copies of all forms to their Group member institution for forwarding to the Southwest Oncology Group Data Operations Center in Seattle at the following address:

Southwest Oncology Group Data Operations Center
Cancer Research And Biostatistics
1730 Minor Ave, STE 1900
Seattle, WA 98101-1468

14.4 WITHIN 14 DAYS OF REGISTRATION:

Submit a copy of the following:

a. **S0512** Advanced Ocular Melanoma Prestudy Form (Form #34876).

b. Baseline Tumor Assessment Form (Form #848).

c. **S0512** Baseline Abnormalities Form (Form #44796).

d. Pathology report documenting histologic confirmation of malignant uveal melanoma.

14.5 SPECIMEN SUBMISSION:

Submit materials at the intervals outlined in Section 15.0.

14.6 AFTER EVERY CYCLE WHILE ON PROTOCOL TREATMENT (1 CYCLE = 21 DAYS):

Submit a copy of the following:

a. **S0512** Cycle-Specific Adverse Event Form (Form #14672)

b. **S0512** Cycle-Specific Treatment Form (Form #656).

14.7 EVERY 6 WEEKS WHILE ON PROTOCOL TREATMENT AND AT CONFIRMATION OF RESPONSE IF CONFIRMATION OCCURS AFTER OFF TREATMENT:

Submit a copy of the Follow-Up Tumor Assessment Form (Form #38305).

14.8 ONCE OFF TREATMENT, EVERY 6 WEEKS UNTIL DISEASE PROGRESSION, THEN EVERY 6 MONTHS FOR THE FIRST 2 YEARS, THEN ANNUALLY FOR UP 3 YEARS FROM THE DATE OF REGISTRATION:

Submit a copy of the following:

a. Follow-Up Tumor Assessment Form (Form #38305) until documented progression of disease.

b. Follow Up Form (Form #64587).

14.9 WITHIN 14 DAYS OF PROGRESSION OR RELAPSE:

Submit a copy of the following:

a. Follow-Up Tumor Assessment Form (Form #38305).

b. Follow Up Form (Form #64587).

If the patient was on treatment at the time of progression, see Section 14.10.

14.10 WITHIN 14 DAYS OF DISCONTINUATION OF ALL PROTOCOL TREATMENT:

Submit a copy of the following:

a. Off Treatment Notice (Form #8756) documenting reasons for off treatment.

b. **S0512** Cycle-Specific Treatment Form (Form #656).

c. **S0512** Cycle-Specific Adverse Event Form (Form #14672).

d. Follow-Up Tumor Assessment Form (Form #38305)

14.13 WITHIN 30 DAYS OF KNOWLEDGE OF DEATH:

Submit a copy of the following:

a. Notice of Death (Form #49467).

b. If the patient was on protocol treatment, see Section 14.10. Otherwise, submit the Follow Up Form (Form #64587).

15.0 SPECIAL INSTRUCTIONS

15.1 General specimen submission instructions

a. **All submitted specimens must be labeled with the protocol number (S0512), SWOG patient number, patient's initials, and date and time of specimen collection.**

b. The Federal guidelines for shipment are as follows:

1. The specimen must be wrapped in an absorbable material.

2. The specimen must be placed in an AIRTIGHT container (like a resealable bag).

3. Pack the resealable bag and specimen in a Styrofoam shipping container.

4. Pack the Styrofoam shipping container in a cardboard box.

5. The cardboard box must be marked as "BIOHAZARD."

c. Specimen Tracking

1. All specimen submissions for patients registered on this study by Southwest Oncology Group institutions and affiliates must be entered and tracked using the SWOG online Specimen Tracking system. SWOG members may log on to the Specimen Tracking system via the CRA Workbench. To access the CRA Workbench, go to the SWOG Web site (http://swog.org) and logon to the Members Area. After you have logged on using your SWOG roster ID numbers and passwords, click on the *CRA Workbench* link to access the home page for CRA Workbench website.

   In the online Specimen Tracking system, laboratory ID numbers are used to identify the laboratories to which specimens are shipped. The laboratory ID number for this study may be found listed next to the laboratory names in Section 15.2c below.

   ALL SPECIMENS MUST BE LOGGED VIA THIS SYSTEM; THERE ARE NO EXCEPTIONS. For any questions or problems regarding the Specimen Tracking program, please send an email to technicalquestion@crab.org.

2. A copy of the Shipment Packing List produced by the Specimen Tracking system should be printed and placed in the pocket of the specimen bag if it has one, or in a separate resealable bag.

3. The bags should then be shipped in a standard Styrofoam shipping container which the sites can supply. PLEASE PACK TUBES CAREFULLY. Cardboard express mail envelopes are NOT ADEQUATE – please additionally pack the tubes in Styrofoam or with extra padding. If freezing conditions or extreme heat conditions are anticipated, insulated containers are recommended.

4. Specimens will not be accepted on weekends or holidays.

NOTE: Every effort should be made to collect the specimens outlined below between 11:00 a.m. and 1:00 p.m. The time of collection is critical because MAPK levels are known to have circadian variation.

15.2 Peripheral Blood Specimens – **Strongly Encouraged**

15 mL of peripheral blood will be collected in EDTA-containing (purple-top) tubes **at prestudy** (at any time prior to the administration of therapy) and **on Day 17** (± 2 days; i.e. collection between Days 15-19 is acceptable) from **11:00 am to 1:00 pm**. Samples should be kept on ice and processed as outlined below within one hour of collection.

Blood tubes should be centrifuged at 1,000 g for 10 min at room temperature. The plasma should be transferred to a new sterile, appropriately labeled 14 mL round-bottom polypropylene tube and frozen at -20ºC. The cell pellet should be re-suspended at room temperature PBS and separated over ficoll-hypaque to yield peripheral blood mononuclear cells (PBMC) at the interface. The PBMC and the red cell pellet should be transferred to two separate new, appropriately labeled, 14 mL round-bottom polypropylene tubes and frozen at -20ºC. Tubes should be submitted on the **day of collection** on **dry ice** to the address in Section 15.5.

15.3 Urine Specimens – **Strongly Encouraged**

One urine sample will be collected in a standard urine container **at prestudy** (at any time prior to the administration of therapy) and **on Day 17** (± 2 days; i.e. collection between Days 15-19 is acceptable) from **11:00 am to 1:00 pm**.

The urine should be transferred to a 14 mL round-bottom polypropylene tube and frozen at -20ºC until shipment. Tubes should be submitted on the **day of collection** on **dry ice** to the address in Section 15.5.

15.4 Tumor Biopsies – **Strongly Encouraged**

a. Paraffin-embedded tissue sections or cytologic material from the patient's initial diagnostic procedure are to be submitted to the address in Section 15.5 **within 30 days after registration** and on **wet ice** to prevent heat damage. Submit 10 double-cut paraffin-embedded unstained slides for immunohistochemistry and either the paraffin block or two 25-micron sections from the paraffin block from the prestudy biopsy specimen.

b. Dr. Fenoglio-Preiser at the University of Cincinnati will forward the specimens to Dr. Ana Aparicio, who will perform the analyses. Microvessel density studies have been funded. VEGF and ERK 1/2 phosphorylation studies will be done only after funding is obtained.

c. With additional patient consent, any specimens not consumed by testing specified in this protocol will be kept in the Southwest Oncology Group Solid Tumor Tissue Bank for future unspecified testing.

15.5 Specimens should be shipped by overnight courier on Monday through Thursday to the following laboratory:

Lab #107: U of Cincinnati/Path and Lab Correlates

Contact: Chris Hackett/Gayle Gatto
Phone: 513/558-4675 or 513/558-1848

15.6 Analysis Methods

Immunohistochemistry

Tumor sample sections will be deparaffinized and stained with hematoxylin and eosin (H&E) to visualize tumor architecture. Serial sections will be deparaffinized and incubated with 10% goat serum at 37° C for 10 minutes and incubated with appropriate dilutions of primary antibodies (1:50 to 1:500) at 4°C overnight. Antibodies for VEGFR-2 will be obtained from R&D Systems (Minneapolis, MN); phospho-VEGFR-2 from Chemicon (Temecula CA); ERK1/2 and phospho-ERK1/2 from Upstate Biotechnology (Lake Placid, NY); antibodies for CD31 will be obtained from Santa Cruz Biotechnology (Santa Cruze, CA). Preimmune or non-immune serum will be used as staining controls. The immunoreactivity for these receptors will be revealed using an avidin-biotin kit from Vector Laboratories (Burlingame, CA). Peroxidase activity will be revealed by the diaminobenzidine (DAB) cytochemical reaction. The slides will then be counterstained with 0.12% methylene blue or Hand E.

Microvascular density (MVD) will be determined after staining the sections with the endothelial specific antibody CD31 by counting the number of vessels in five representative 1 mm^2 sections. The AngioSys software from TCS Cellworks (Buckingham UK) will be used to automate analysis of MVD.

VEGF ELISA

VEGF levels in patient samples will be determined using a solid phase double-ligand enzyme-linked immunosorbent assay (ELISA) obtained from R&D Systems (Minneapolis, MN) according to the manufacturer's instructions. Microtiter plates are provided coated with monoclonal antibody specific for VEGF. Standards, samples, and VEGF antibody conjugated to horseradish peroxidase will be pipetted into the wells and any VEGF present will be sandwiched by the immobilized antibody and the enzyme-linked polyclonal antibody specific for VEGF. After removal of excess of any unbound substances and/or antibody-enzyme reagent, a substrate solution (hydrogen peroxide

and chromogen tetramethylbenzidine) will be added to the wells and color developed in proportion to the amount of VEGF bound. The color development will be stopped after 30 min at room temperature and the intensity of the color measured at 450 nm in a microplate reader (Molecular Devices, Sunnyvale, CA).

16.0 ETHICAL AND REGULATORY CONSIDERATIONS

The following must be observed to comply with Food and Drug Administration regulations for the conduct and monitoring of clinical investigations; they also represent sound research practice:

Informed Consent

The principles of informed consent are described by Federal Regulatory Guidelines (Federal Register Vol. 46, No. 17, January 27, 1981, part 50) and the Office for Protection from Research Risks Reports: Protection of Human Subjects (Code of Federal Regulations 45 CFR 46). They must be followed to comply with FDA regulations for the conduct and monitoring of clinical investigations.

Institutional Review

This study must be approved by an appropriate institutional review committee as defined by Federal Regulatory Guidelines (Ref. Federal Register Vol. 46, No. 17, January 27, 1981, part 56) and the Office for Protection from Research Risks Reports: Protection of Human Subjects (Code of Federal Regulations 45 CFR 46).

Drug Accountability

For each investigational drug supplied for a study, drug disposition (drug receipt, dispensing, transfer or return) shall be maintained on the NCI Investigational Drug Accountability Record. Drug supplies must be kept in a secure, limited access storage area under the recommended storage conditions. During the course of the study, the following information must be noted on the Drug Accountability Record; the SWOG ID # and initials of the subject to whom drug is dispensed, the dose, the date(s) and quantity of drug dispensed to the subject, the date(s) and quantity of drug returned to the NCI or transferred to another NCI-approved protocol, the balance forward, lot number and recorder's initials. These Drug Accountability Records must be readily available for inspection and are open to FDA or NCI inspection at any time.

Publication and Industry Contact

The agent supplied by CTEP, DCTD, NCI used in this protocol is provided to the NCI under a Collaborative Agreement (CRADA, CTA) between the Pharmaceutical Company(ies) (hereinafter referred to as Collaborator and the NCI Division of Cancer Treatment and Diagnosis. Therefore, the following obligations/guidelines, in addition to the provisions in the Intellectual Property Option to Collaborator (http:// ctep.cancer.gov/industry) contained within the terms of award, apply to the use of the Agent in this study:

1. Agent may not be used for any purpose outside the scope of this protocol, nor can Agent be transferred or licensed to any party not participating in the clinical study. Collaborator(s) data for Agent are confidential and proprietary to Collaborator and shall be maintained as such by the investigators. The protocol documents for studies utilizing investigational Agents contain confidential information and should not be shared or distributed without the permission of the NCI. If a copy of this protocol is requested by a patient or patient’s family member participating on the study, the individual should sign a confidentiality agreement. A suitable model agreement can be downloaded from: http://ctep.cancer.gov.

2. For a clinical protocol where there is an investigational Agent used in combination with (an)other investigational Agent, each the subject of different collaborative agreements , the access to and use of data by each Collaborator shall be as follows (data pertaining to such combination use shall hereinafter be referred to as "Multi-Party Data"):

   a. NCI will provide all Collaborators with prior written notice regarding the existence and nature of any agreements governing their collaboration with NIH, the design of the proposed combination protocol, and the existence of any obligations that would tend to restrict NCI's participation in the proposed combination protocol.

   b. Each Collaborator shall agree to permit use of the Multi-Party Data from the clinical trial by any other Collaborator solely to the extent necessary to allow said other Collaborator to develop, obtain regulatory approval or commercialize its own investigational Agent.

   c. Any Collaborator having the right to use the Multi-Party Data from these trials must agree in writing prior to the commencement of the trials that it will use the Multi-Party Data solely for development, regulatory approval, and commercialization of its own investigational Agent.

3. Clinical Trial Data and Results and Raw Data developed under a Collaborative Agreement will be made available exclusively to Collaborator, the NCI, and the FDA, as appropriate and unless additional disclosure is required by law or court order. Additionally, all Clinical Data and Results and Raw Data will be collected , used and disclosed consistent with all applicable federal statutes and regulations for the protection of human subjects, including, if applicable, the *Standards for Privacy of Individually Identifiable Health Information* set forth in 45 C.F.R. Part 164.

4. When a Collaborator wishes to initiate a data request, the request should first be sent to the NCI, who will then notify the appropriate investigators (Group Chair for Cooperative Group studies, or PI for other studies) of Collaborator's wish to contact them.

5. Any data provided to Collaborator(s) for Phase 3 studies must be in accordance with the guidelines and policies of the responsible Data Monitoring Committee (DMC), if there is a DMC for this clinical trial.

6. Any manuscripts reporting the results of this clinical trial must be provided to CTEP for immediate delivery to Collaborator for advisory review and comment prior to submission for publication. Collaborator will have 30 days from the date of receipt for review. Collaborator shall have the right to request that publication be delayed for up to an additional 30 days in order to ensure that Collaborator’s confidential and proprietary data, in addition to Collaborator’s intellectual property rights, are protected. Copies of abstracts must be provided to CTEP for forwarding to Collaborator for courtesy review as soon as possible and preferably at least three (3) days prior to submission, but in any case, prior to presentation at the meeting or publication in the proceedings. Press releases and other media presentations must also be forwarded to CTEP prior to release. Copies of any manuscript, abstract and/or press release/media presentation should be sent to:

   Regulatory Affairs Branch, CTEP, DCTD, NCI
   Executive Plaza North, Suite 7111
   Bethesda, Maryland 20892
   FAX: 301/402-1584
   Email: anshers@ctep.nci.nih.gov

The Regulatory Affairs Branch will then distribute them to Collaborator. No publication, manuscript or other form of public disclosure shall contain any of Collaborators confidential/proprietary information.

Monitoring

This study will be monitored by the Clinical Data Update System (CDUS) Version 3.0. Cumulative CDUS data will be submitted quarterly to CTEP by electronic means. Reports are due January 31, April 30, July 31 and October 31.

16.1 Adverse Event Reporting Requirements

a. Purpose

Adverse event data collection and reporting, which are required as part of every clinical trial, are done to ensure the safety of patients enrolled in the studies as well as those who will enroll in future studies using similar agents. Adverse events are reported in a routine manner at scheduled times during a trial. (Directions for routine reporting are provided in Section 14.0.) Additionally, certain adverse events must be reported in an expedited manner to allow for more timely monitoring of patient safety and care. The following guidelines prescribe expedited adverse event reporting for this protocol. See also Appendix 19.1 for general and background information about expedited reporting.

b. Reporting methods

This study requires that expedited adverse event reporting use the NCI's Adverse Event Expedited Reporting System (AdEERS). The NCI's guidelines for AdEERS can be found at http://ctep.cancer.gov. An AdEERS report must be submitted to the Southwest Oncology Group Operations Office by one of the following methods:

- Electronically submit the report via the AdEERS Web-based application located at http://ctep.cancer.gov, or

- **Only if submitting electronically is not possible**, fax the completed NCI Adverse Event Expedited Report – Single Agent or Multiple Agents – paper template, located at http://ctep.cancer.gov, to 301/230-0159 within the appropriate time frame per Table 16.1. Once Internet connectivity is restored, an AE report submitted on a paper template must be entered electronically into AdEERS by the original submitter at the site.

c. When to report an event in an expedited manner

Some adverse events require 24-hour notification (refer to Table 16.1) via AdEERS. When Internet connectivity is disrupted, a 24-hour notification is to be made to CTEP by telephone at 301/897-7497. Once Internet connectivity is restored, a 24-hour notification phoned in, must be entered electronically into AdEERS by the original submitter at the site.

When the adverse event requires expedited reporting, submit the report within the number of calendar days of learning of the event specified in Table 16.1 or 16.2, as applicable.

d. Other recipients of adverse event reports

The Operations Office will forward reports and documentation to the appropriate regulatory agencies and drug companies as required.

Adverse events determined to be reportable must also be reported according to local policy and procedures to the Institutional Review Board responsible for oversight of the patient.

e. Expedited reporting for investigational agents

Expedited reporting is required if the patient has received at least one dose of the investigational agent as part of the trial. Reporting requirements are provided in Table 16.1. The investigational agent used in this study is BAY 43-9006 (sorafenib). If there is any question about the reportability of an adverse event or if on-line AdEERS cannot be used, please telephone or email the SAE Specialist at the Operations Office, 210/677-8808 or adr@swog.org, before preparing the report.

Table 16.1. Phase 2 and 3 Trials Utilizing an Agent under a CTEP IND or Non-CTEP IND: AdEERS Expedited Reporting Requirements for Adverse Events that Occur within 30 Days[1] of the Last Dose of the Investigational Agent BAY 43-9006 (sorafenib) in this Study

	Grade 1	Grade 2	Grade 2	Grade 3		Grade 3		Grades 4 & 5[2]	Grades 4 & 5[2]
	Unexpected and Expected	Unexpected	Expected	Unexpected		Expected		Unex-pected	Expected
				with Hospitali-zation	without Hospitali-zation	with Hospitali-zation	without Hospitali-zation		
**Unrelated Unlikely**	Not Required	Not Required	Not Required	10 Calendar Days	Not Required	10 Calendar Days	Not Required	10 Calendar Days	10 Calendar Days
**Possible Probable Definite**	Not Required	10 Calendar Days	Not Required	10 Calendar Days	10 Calendar Days	10 Calendar Days	Not Required	24-Hour; 5 Calendar Days	10 Calendar Days

[1] **Adverse events with attribution of possible, probable, or definite that occur greater than 30 days after the last dose of treatment with an agent under a CTEP IND or Non-CTEP IND require reporting as follows:**

AdEERS 24-hour notification (**via AdEERS for CTEP IND agents; via email to *adr@swog.org* for agents in non-CTEP IND studies**) followed by complete report within 5 calendar days for:

- Grade 4 and Grade 5 unexpected events

AdEERS 10 calendar day report:

- Grade 3 unexpected events with hospitalization or prolongation of hospitalization
- Grade 5 expected events

[2] Although an AdEERS 24-hour notification is not required for death clearly related to progressive disease, a full report is required as outlined in the table.

March 2005

NOTE: All deaths on study require both routine and expedited reporting regardless of causality. Attribution to treatment or other cause must be provided.

- Expedited AE reporting timelines defined:
  - "24 hours; 5 calendar days" – The investigator must initially report the AE via AdEERS within 24 hours of learning of the event followed by a complete AdEERS report within 5 calendar days of the initial 24-hour report.

- "10 calendar days" - A complete AdEERS report on the AE must be submitted within 10 calendar days of the investigator learning of the event.

- Any medical event equivalent to CTCAE Grade 3, 4 or 5 that precipitates hospitalization (or prolongation of existing hospitalization) must be reported regardless of attribution and designation as expected or unexpected with the exception of any events identified as protocol-specific expedited adverse event reporting exclusions.

- Any event that results in persistent or significant disabilities/incapacities, congenital anomalies, or birth defects must be reported via AdEERS if the event occurs following treatment with an agent under a CTEP IND.

- Use the NCI protocol number and the protocol-specific patient ID assigned during trial registration on all reports.

f. Expedited reporting for commercial agents

Commercial reporting requirements are provided in Table 16.2. The commercial agents used in this study are carboplatin and paclitaxel. If there is any question about the reportability of an adverse event, please telephone or email the SAE Program at the Operations Office, 210/677-8808 or adr@swog.org, before preparing the report.

Table 16.2. Expedited reporting requirements for adverse events experienced by patients on this study arm who have received the commercial drugs listed in 16.1f above.

	**Grade 4**		**Grade 5[a]**	
	Unexpected	Expected	Unexpected	Expected
Unrelated or Unlikely			**AdEERS**	**AdEERS**
Possible, Probable, Definite	**AdEERS**		**AdEERS**	**AdEERS**

AdEERS: Indicates an expedited report is to be submitted using the NCI AdEERS system Commercial Drug pathwaywithin 7 working days of learning of the event.

a This includes all deaths within 30 days of the last dose of treatment with a commercial agent(s), regardless of attribution. Any death that occurs more than 30 days after the last dose of treatment with a commercial agent(s) and is attributed (possibly, probably, or definitely) to the agent(s) and is not due to cancer recurrence must be reported according to the instructions above.

g. Reporting secondary AML/MDS

All cases of acute myeloid leukemia (AML), myelodysplastic syndrome (MDS), and acute lymphocytic leukemia (ALL) that occur in patients on NCI-sponsored trials following chemotherapy for cancer must be reported using the NCI/CTEP Secondary AML/MDS Report Form in lieu of AdEERS. The following supporting documentation must also be submitted within 30 days:

- a copy of the pathology report confirming the AML/MDS/ALL diagnosis; and
- (if available) a copy of the cytogenetics report.

Submit the Report and documentation to:

Investigational Drug Branch<br>by fax to 301-230-0159<br>or mail to P.O. Box 30012<br>Bethesda, MD 20824-0012	**<u>and</u>**	Southwest Oncology Group<br>ATTN: SAE Program<br>14980 Omicron Drive<br>San Antonio, Texas 78245-3217

NOTE: If a patient has been enrolled in more than one NCI-sponsored study, the AML/MDS Report must be submitted for the most recent trial.

17.0 BIBLIOGRAPHY

1. Sahel JA, Polans A, Mehta MP, et al. Intraocular melanoma. In: DeVita VT Jr., Hellman S, Rosenberg SA, eds. Cancer Principles and Practices of Oncology, pp. 2070-90. 6th ed: Lippincot Williams & Wilkins; 2001.

2. Robertson DM. Changing concepts in the management of choroidal melanoma. Am J Ophthalmol 136(1):161-70, 2003.

3. Mudhar HS, Parsons MA, Sisley K, et al. A critical appraisal of the prognostic and predictive factors for uveal malignant melanoma. Histopathology 45(1):1-12, 2004.

4. Diener-West M, Reynolds SM, Agugliaro DJ, et al. Screening for metastasis from choroidal melanoma: the Collaborative Ocular Melanoma Study Group Report 23. J Clin Oncol 22(12):2438-44, 2004.

5. Eskelin S, Pyrhonen S, Hahka-Kemppinen M, et al. A prognostic model and staging for metastatic uveal melanoma. Cancer 97(2):465-75; 2003.

6. Leyvraz S, Spataro V, Bauer J, et al. Treatment of ocular melanoma metastatic to the liver by hepatic arterial chemotherapy. J Clin Oncol 15(7):2589-95, 1997.

7. Pyrhonen S, Hahka-Kemppinen M, Muhonen T, et al. Chemoimmunotherapy with bleomycin, vincristine, lomustine, dacarbazine (BOLD), and human leukocyte interferon for metastatic uveal melanoma. Cancer 95(11):2366-72, 2002.

8. Becker JC, Terheyden P, Kampgen E, et al. Treatment of disseminated ocular melanoma with sequential fotemustine, interferon alpha, and interleukin 2. Br J Cancer 87(8):840-5, 2002.

9. Alexander HR, Jr., Libutti SK, Pingpank JF, et al. Hyperthermic isolated hepatic perfusion using melphalan for patients with ocular melanoma metastatic to liver. Clin Cancer Res 9(17):6343-9, 2003.

10. Bedikian AY, Legha SS, Mavligit G, et al. Treatment of uveal melanoma metastatic to the liver: a review of the M. D. Anderson Cancer Center experience and prognostic factors. Cancer 76(9):1665-70, 1995.

11. Flaherty LE, Unger JM, Liu PY, el al. Metastatic melanoma from intraocular primary tumors: the Southwest Oncology Group experience in phase II advanced melanoma clinical trials. Am J Clin Oncol 21(6):568-72, 1998.

12. Nathan FE, Berd D, Sato T, et al. BOLD+interferon in the treatment of metastatic uveal melanoma: first report of active systemic therapy. J Exp Clin Cancer Res 16(2):201-8, 1997.

13. Pfohler C, Cree IA, Ugurel S, et al. Treosulfan and gemcitabine in metastatic uveal melanoma patients: results of a multicenter feasibility study. Anticancer Drugs 14(5):337-40, 2003.

14. Keilholz U, Schuster R, Schmittel A, et al. A clinical phase I trial of gemcitabine and treosulfan in uveal melanoma and other solid tumours. Eur J Cancer 40(14):2047-52, 2004.

15. Schmidt-Hieber M, Schmittel A, Thiel E, et al. A phase II study of bendamustine chemotherapy as second-line treatment in metastatic uveal melanoma. Melanoma Res 14(6):439-42, 2004.

16. Feldman ED, Pingpank JF, Alexander HR, Jr. Regional treatment options for patients with ocular melanoma metastatic to the liver. Ann Surg Oncol 11(3):290-7, 2004.

17. Papachristou DN, Fortner JJ. Surgical treatment of metastatic melanoma confined to the liver. Int Surg 68(2):145-8, 1983.

18. Fournier GA, Albert DM, Arrigg CA, et al. Resection of solitary metastasis. Approach to palliative treatment of hepatic involvement with choroidal melanoma. Arch Ophthalmol 102(1):80-2, 1984.

19. Gunduz K, Shields JA, Shields CL, et al. Surgical removal of solitary hepatic metastasis from choroidal melanoma. Am J Ophthalmol 125(3):407-9, 1998.

20. Aoyama T, Mastrangelo MJ, Berd D, et al. Protracted survival after resection of metastatic uveal melanoma. Cancer 89(7):1561-8, 2000.

21. Mavligit GM, Charnsangavej C, Carrasco CH, et al. Regression of ocular melanoma metastatic to the liver after hepatic arterial chemoembolization with cisplatin and polyvinyl sponge. Jama 260(7):974-6, 1988.

22. Feun LG, Reddy KR, Scagnelli T, et al. A phase I study of chemoembolization with cisplatin, thiotepa, and lipiodol for primary and metastatic liver cancer. Am J Clin Oncol 22(4):375-80, 1999.

23. Kotak D, Sotti M, Mastrangelo MJ, et al. Survival benefit of immunoembolization with granulocyte-macrophage colony stimulating factor (GM-CSF) in patients with metastatic uveal melanoma with liver metastases. In: Oncol PASC, editor. ASCO p. 723; a#2909, 2003.

24. Cantore M, Fiorentini G, Aitini E, et al. Intra-arterial hepatic carboplatin-based chemotherapy for ocular melanoma metastatic to the liver. Report of a phase II study. Tumori 80(1):37-9, 1994.

25. Alexander HR, Libutti SK, Bartlett DL, et al. A phase I-II study of isolated hepatic perfusion using melphalan with or without tumor necrosis factor for patients with ocular melanoma metastatic to liver. Clin Cancer Res 6(8):3062-70, 2000.

26. Noter SL, Rothbarth J, Pijl ME, et al. Isolated hepatic perfusion with high-dose melphalan for the treatment of uveal melanoma metastases confined to the liver. Melanoma Res 14(1):67-72, 2004.

27. Singh AD, Wang MX, Donoso LA, et al. Genetic aspects of uveal melanoma: a brief review. Semin Oncol 23(6):768-72, 1996.

28. Pollock PM, Trent JM. The genetics of cutaneous melanoma. Clin Lab Med 20(4):667-90, 2000.

29. Onken MD, Worley LA, Ehlers JP, et al. Gene expression profiling in uveal melanoma reveals two molecular classes and predicts metastatic death. Cancer Res 64(20):7205-9, 2004.

30. Sebolt-Leopold JS, Herrera R. Targeting the mitogen-activated protein kinase cascade to treat cancer. Nat Rev Cancer 4(12):937-47, 2004.

31. Davies H, Bignell GR, Cox C, et al. Mutations of the BRAF gene in human cancer. Nature 417(6892):949-54, 2002.

32. Karasarides M, Chiloeches A, Hayward R, et al. B-RAF is a therapeutic target in melanoma. Oncogene 23(37):6292-8, 2004.

33. Mooy CM, Van der Helm MJ, Van der Kwast TH, et al. No N-ras mutations in human uveal melanoma: the role of ultraviolet light revisited. Br J Cancer 64(2):411-3, 1991.

34. Soparker CN, O'Brien JM, Albert DM. Investigation of the role of the ras protooncogene point mutation in human uveal melanomas. Invest Ophthalmol Vis Sci 34(7):2203-9, 1993.

35. Cruz F, 3rd, Rubin BP, Wilson D, et al. Absence of BRAF and NRAS mutations in uveal melanoma. Cancer Res 63(18):5761-6, 2003.

36. Edmunds SC, Cree IA, Di Nicolantonio F, et al. Absence of BRAF gene mutations in uveal melanomas in contrast to cutaneous melanomas. Br J Cancer 88(9):1403-5, 2003.

37. Cohen Y, Goldenberg-Cohen N, Parrella P, et al. Lack of BRAF mutation in primary uveal melanoma. Invest Ophthalmol Vis Sci 44(7):2876-8, 2003.

38. Rimoldi D, Salvi S, Lienard D, et al. Lack of BRAF mutations in uveal melanoma. Cancer Res 63(18):5712-5, 2003.

39. Wilhelm SM, Carter C, Tang L,et al. BAY 43-9006 exhibits broad spectrum oral antitumor activity and targets the RAF/MEK/ERK pathway and receptor tyrosine kinases involved in tumor progression and angiogenesis. Cancer Res Oct 1;64(19):7099-109, 2004.

40. Choi YJ, Fanidi A, White S, et al. The novel Raf inhibitor BAY 43-9006 blocks signaling and proliferation in BRAF mutant and wildtype melanoma and colorectal tumor cell lines. In: AACR Po, editor. AACR a#720, 2004.

41. Marais R. BRAF is an important therapeutic target in human cancer. In: AACR-NCI-EORTC P, editor. Molecular Targets and Cancer Therapeutics p. 249, 2003.

42. Heim M, Sharifi M, Hilger RA, et al. Antitumor effect and potentiation or reduction in cytotoxic drug activity in human colon carcinoma cells by the Raf kinase inhibitor (RKI) BAY 43-9006. Int J Clin Pharmacol Ther Dec;41(12):616-7, 2003.

43. BAY 43-9006 Investigator Brochure.

44. Ratain MJ, Stadler W, Smith M, et al. A phase II study of BAY 43-9006 using the randomized discontinuation design in patients with advanced refractory cancer. In: AACR-NCI-EORTC P, editor. Molecular Targets and Cancer Therapeutics a#C254, 2003.

45. Flaherty KT, Lee RJ, Humphries R, et al. Phase I trial of BAY 43-9006 in combination with carboplatin (C) and paclitaxel (P). In: Oncol PASC, editor. ASCO a#2854, 2003.

46. Schiller JH, Harrington D, Belani CP, et al. Comparison of four chemotherapy regimens for advanced non-small-cell lung cancer. NEJM 346(2):92-8, 2002.

47. Uzzan B, Nicolas P, Cucherat M, et al. Microvessel density as a prognostic factor in women with breast cancer: a systematic review of the literature and meta-analysis. Cancer Res 64(9):2941-55, 2004.

48. Chow S, Patel H, Hedley DW. Measurement of MAP kinase activation by flow cytometry using phospho-specific antibodies to MEK and ERK: potential for pharmacodynamic monitoring of signal transduction inhibitors. Cytometry 15;46(2):72-8, 2001.

49. Strumberg D, Richly H, Hilger RA, et al. Phase I clinical and pharmacokinetic study of the Novel Raf kinase and vascular endothelial growth factor receptor inhibitor BAY 43-9006 in patients with advanced refractory solid tumors. J Clin Oncol 10;23(5):965-72. Epub 2004 Dec 21, 2005.

50. Hilger RA, Scheulen ME, Strumberg D. The Ras-Raf-MEK-ERK pathway in the treatment of cancer. Onkologie 25(6):511-8, 2002.

51. Dvorak HF, Brown LF, Detmar M, et al. Vascular permeability factor/vascular endothelial growth factor, microvascular hyperpermeability, and angiogenesis. Am J Pathol 146(5):1029-39, 1995.

52. Senger DR, Van de Water L, Brown LF, et al. Vascular permeability factor (VPF, VEGF) in tumor biology. Cancer Metastasis Rev 12(3-4):303-24, 1993.

53. Maeda K, Chung YS, Ogawa Y, et al. Prognostic value of vascular endothelial growth factor expression in gastric carcinoma. Cancer 1;77(5):858-63, 1996.

54. Masood R, Cai J, Zheng T, et al. Vascular endothelial growth factor/vascular permeability factor is an autocrine growth factor for AIDS-Kaposi sarcoma. Proc Natl Acad Sci U S A Feb 4;94(3):979-84, 1997.

55. deVries C, Escobedo JA, Veno H, et al. The fms-like tyrosine kinase, a receptor for vascular endothelial growth factor. Science 255 (5047):989-91, 1992.

56. Terman BI, Dougher-Vermazen M, Carrion ME, et al. Identification of the KDR tyrosine kinase as a receptor for vascular endothelial cell growth factor. Biochem Biophys Res Commun 30;187(3):1579-86, 1992.

57. Soker S, Takashima S, Miao HQ, et al. Neuropilin-1 is expressed by endothelial and tumor cells as an isoform-specific receptor for vascular endothelial growth factor. Cell 20;92(6):735-45, 1998.

58. Waltenberger J, Claesson-Welsh L, Siegbahn A, et al. Different signal transduction properties of KDR and Flt1, two receptors for vascular endothelial growth factor. J Biol Chem 28;269(43):26988-95, 1994.

59. Siemeister G, Schnurr B, Mohrs K, et al. Expression of biologically active isoforms of the tumor angiogenesis factor VEGF in Escherichia coli. Biochem Biophys Res Commun 5;222(2):249-55, 1996.

60. Clauss M, Weich H, Breier G, et al. The vascular endothelial growth factor receptor Flt-1 mediates biological activities. Implications for a functional role of placenta growth factor in monocyte activation and chemotaxis. J Biol Chem Jul 26;271(30):17629-34, 1996.

61. Tamura K, Nakajima H, Rakue H, et al. Elevated circulating levels of basic fibroblast growth factor and vascular endothelial growth factor in patients with acute myocardial infarction. Jpn Circ J 63(5):357-61, 1999.

18.0 MASTER FORMS SET

18.1 The Model Informed Consent Form is included in this section, preceded by "Notes for Local Institution Consent Form Authors" and "Notes for Local Investigators." The study - as well as the local consent form meeting the guidelines noted in these documents - must be reviewed and approved by the Institutional Review Board prior to registration and treatment of patients on this study.

18.2 Registration Form (Form #63366)

18.3 **S0512** Advanced Ocular Melanoma Prestudy Form (Form #34876).

18.4 Baseline Tumor Assessment Form (Form #848) (9/1/03)

18.5 **S0512** Baseline Abnormalities Form (Form #44796).

18.6 **S0512** Cycle-Specific Treatment Form (Form #656).

18.7 **S0512** Cycle-Specific Adverse Event Form (Form #14672).

18.8 Follow Up Form (Form #64587) (9/15/03)

18.9 Follow-Up Tumor Assessment (Form #38305) (9/1/03)

18.10 Off Treatment Notice (Form #8756) (9/1/03)

18.11 Notice of Death (Form #49467) (9/1/03)

Informed Consent Model for S0512

*NOTES FOR LOCAL INSTITUTION INFORMED CONSENT AUTHORS:

This model informed consent form has been reviewed by the DCT/NCI and is the official consent document for this study. Local IRB changes to this document are allowed. (Institutions should attempt to use sections of this document that are in bold type in their entirety.) Editorial changes to these sections may be made as long as they do not change information or intent. If the institutional IRB insists on making deletions or more substantive modifications to the risks or alternatives sections, they may be justified in writing by the investigator and approved by the IRB. Under these circumstances, the revised language, justification and a copy of the IRB minutes must be forwarded to the Southwest Oncology Group Operations Office for approval before a patient may be registered to this study.

Please particularly note that the questions related to banking of specimens for future study are in bolded type and may not be changed in any way without prior approval from the Southwest Oncology Group Operations Office.

Readability Statistics:	
Flesch Reading Ease	57.1 (targeted above 55)
Flesch-Kincaid Grade Level	9.1 (targeted below 8.5)

- Instructions and examples for informed consent authors are in *[italics]*.
- A blank line, __________, indicates that the local investigator should provide the appropriate information before the document is reviewed with the prospective research participant.
- The term "study doctor" has been used throughout the model because the local investigator for a cancer treatment trial is a physician. If this model is used for a trial in which the local investigator is not a physician, another appropriate term should be used instead of "study doctor".
- The dates of protocol updates in the header and in the text of the consent is for reference to this model only and should not be included in the informed consent form given to the prospective research participant.
- The local informed consent must state which parties may inspect the research records. This includes the NCI, the drug manufacturer for investigational studies, any companies or grantors that are providing study support (these will be listed in the protocol's model informed consent form) and the Southwest Oncology Group.

  The "Southwest Oncology Group" must be listed as one of the parties that may inspect the research records in all protocol consent forms for which patient registration is being credited to the Southwest Oncology Group. This includes consent forms for studies where all patients are registered directly through the Southwest Oncology Group Data Operations Office, all intergroup studies for which the registration is being credited to the Southwest Oncology Group (whether the registration is through the SWOG Data Operations Office or directly through

the other group), as well as consent forms for studies where patients are registered via CTSU and the registration is credited to the Southwest Oncology Group.

- **When changes to the protocol require revision of the informed consent document, the IRB should have a system that identifies the revised consent document, in order to preclude continued use of the older version and to identify file copies. An appropriate method to identify the current version of the consent is for the IRB to stamp the final copy of the consent document with the approval date. The stamped consent document is then photocopied for use. Other systems of identifying the current version of the consent such as adding a version or approval date are allowed as long as it is possible to determine during an audit that the patient signed the most current version of the consent form.**

*NOTES FOR LOCAL INVESTIGATORS:

- The goal of the informed consent process is to provide people with sufficient information for making informed choices. The informed consent form provides a summary of the clinical study and the individual's rights as a research participant. It serves as a starting point for the necessary exchange of information between the investigator and potential research participant. This model for the informed consent form is only one part of the larger process of informed consent. For more information about informed consent, review the "Recommendations for the Development of Informed Consent Documents for Cancer Clinical Trials" prepared by the Comprehensive Working Group on Informed Consent in Cancer Clinical Trials for the National Cancer Institute. The Web site address for this document is http://cancer.gov/clinicaltrials/understanding/simplification-of-informed-consent-docs/
- A blank line, __________, indicates that the local investigator should provide the appropriate information before the document is reviewed with the prospective research participant.
- Suggestion for Local Investigators: An NCI pamphlet explaining clinical trials is available for your patients. The pamphlet is entitled: "If You Have Cancer…What You Should Know about Clinical Trials". This pamphlet may be ordered on the NCI Web site at *http://cissecure.nci.nih.gov/ncipubs/details.asp?pid=1035* or call 1-800-4- CANCER (1-800-422-6237) to request a free copy.
- Optional feature for Local Investigators: Reference and attach drug sheets, pharmaceutical information for the public, or other material on risks. Check with your local IRB regarding review of additional materials.

*These notes for authors and investigators are instructional and should not be included in the informed consent form given to the prospective research participant.

S0512, "Phase II Trial of BAY 43-9006 (Sorafenib; NSC-724772) in Combination with Carboplatin and Paclitaxel in Patients with Metastatic Uveal Melanoma"

This is a clinical trial, a type of research study. Your study doctor will explain the clinical trial to you. Clinical trials include only people who choose to take part. Please take your time to make your decision about taking part. You may discuss your decision with your friends and family. You can also discuss it with your health care team. If you have any questions, you can ask your study doctor for more explanation.

You are being asked to take part in this study because you have uveal melanoma that has returned after treatment or has spread to other parts of your body. Uveal melanoma is melanoma that started in the eye and is also called ocular melanoma or choroidal melanoma.

Why is this study being done?

The purpose of this study is to….

Find out what effects, good and bad, a new investigational agent, BAY 43-9006 (sorafenib), in combination with carboplatin and paclitaxel has on you and your uveal melanoma.

BAY 43-9006 (sorafenib) is an investigational drug with promising anti-cancer activity against a number of tumor types, including other types of melanoma. BAY 43-9006 (sorafenib) is a drug that inhibits a protein in cells called RAF-kinase. Patients who have received this drug in early studies have tolerated the treatment well. This research is being done because currently there is no curative treatment for this type of cancer.

BAY 43-9006 (sorafenib) has been combined with carboplatin and paclitaxel (two well known chemotherapeutic agents that are approved by the FDA for the treatment of other types of cancer) in an effort to improve the efficacy of the treatment. This is the first time this combination is being tested in a clinical trial in which only patients with metastatic uveal melanoma will participate.

How many people will take part in the study?

About 20 - 40 people will take part in this study.

What will happen if I take part in this research study?

Before you begin the study …

You will need to have the following exams, tests or procedures to find out if you can be in the study. These exams, tests or procedures are part of regular cancer care and may be done even if

you do not join the study. If you have had some of them recently, they may not need to be repeated. This will be up to your study doctor. These exams, tests or procedures can be done on an outpatient basis.

- Medical history and physical examination
- Blood tests for blood counts and to test your kidney and liver function
- X-ray, CT scan or MRI to assess disease
- Blood pressure
- Bone scan (if your cancer has spread to your bones)

During the study …

If the exams, tests and procedures show that you can be in the study, and you choose to take part, then you will need the following tests and procedures. They are part of regular cancer care. These exams, tests or procedures can be done on an outpatient basis.

- History and physical exam – This will be performed every 3 weeks.
- Blood pressure – This will be monitored at least weekly for the first 4 weeks of treatment or until stable (if done at home, results should be reported back to your doctor weekly).
- Blood tests for blood counts – These will be performed every week during treatment for the first four weeks and then every 3 weeks.
- Blood tests for kidney and liver function – These will be performed every 3 weeks.
- X-rays/scans – These will be performed every 6 weeks.

If you take part in this study, you will take the experimental drug, BAY 43-9006 (sorafenib) in combination with two known chemotherapy agents: carboplatin and paclitaxel. Carboplatin and paclitaxel will be given to you intravenously (through a needle into your vein) once every three weeks. You will receive the drug, BAY 43-9006 (sorafenib), to be taken as two pills, by mouth, twice a day, beginning on the day after you are given the intravenous chemotherapy. You will continue to take BAY 43-9006 (sorafenib) daily until three days before receiving the next dose of intravenous chemotherapy (approximately 18 days).

You will swallow BAY 43-9006 (sorafenib) whole with a full cup of water. BAY 43-9006 (sorafenib) may be taken with meals or by itself. However, if taken with meals, you should take BAY 43-9006 (sorafenib) with a moderate to low-fat meal. A high-fat meal may decrease the amount of BAY 43-9006 (sorafenib) that gets into your blood. Talk to your study doctor if you have questions about what constitutes a high-fat meal.

Each three-week period will be considered one cycle of treatment. You will continue repeating your cycles of treatment with carboplatin and paclitaxel followed by BAY 43-9006 (sorafenib). After the sixth cycle of treatment, the carboplatin and paclitaxel will be discontinued and BAY 43-9006 (sorafenib) will continue.

You will record the number of pills you take each day on a calendar. You should bring your calendar with you each time you have an appointment. During your visits, your calendar will be reviewed.

How long will I be in the study?

You will be asked to take carboplatin, paclitaxel and BAY 43-9006 (sorafenib) together for the first 6 cycles (18 weeks) of treatment, assuming your disease does not grow and you are tolerating the treatment well. At the end of 6 cycles (18 weeks) the chemotherapy will be stopped and you will continue to receive BAY 43-90006 (sorafenib) until your disease gets worse or your side effects become too great. After you are finished taking carboplatin, paclitaxel and BAY 43-9006 (sorafenib), the study doctor will ask you to visit the office for follow-up exams once every 6 months for up to 2 years and then annually thereafter until 3 years from the time you started the study.

Can I stop being in the study?

Yes. You can decide to stop at any time. Tell the study doctor if you are thinking about stopping or decide to stop. He or she will tell you how to stop safely.

It is important to tell the study doctor if you are thinking about stopping so any risks from carboplatin, paclitaxel and BAY 43-9006 (sorafenib) can be evaluated by your doctor. Another reason to tell your doctor that you are thinking about stopping is to discuss what follow-up care and testing could be most helpful for you.

The study doctor may stop you from taking part in this study at any time if he/she believes it is in your best interest, if you do not follow the study rules, or if the study is stopped.

What side effects or risks can I expect from being in the study?

You may have side effects while on the study. Everyone taking part in the study will be watched carefully for any side effects. However, doctors don't know all the side effects that may happen. Side effects may be mild or very serious. Your health care team may give you medicines to help lessen side effects. Many side effects go away soon after you stop taking the carboplatin, paclitaxel and BAY 43-9006 (sorafenib). In some cases, side effects can be serious, long lasting, or may never go away. There also is a risk of death.

You should talk to your study doctor about any side effects that you have while taking part in the study.

Risks and side effects related to the carboplatin and paclitaxel include those which are:

Likely

- **Decreased white blood cells, which can lead to infection**
- **Decreased red blood cells (anemia), which may make you weak and tired**

- **Decreased platelets, which may increase the risk of bleeding**
- **Nausea and/or vomiting**
- **Fatigue**
- **Painful prickliness or tingling sensation of the fingers and toes**
- **Moderate to severe temporary hair loss (not only from the scalp, but also from underarms, beard, chest, pubic area, arms and legs) is likely to occur but should grow back within a few weeks of discontinuing the carboplatin and paclitaxel**
- **Loss of appetite**
- **Difficulty sleeping**
- **Constipation**
- **Altered taste**

<u>Less Likely</u>

- **Mouth sores**
- **Metabolic changes (calcium, magnesium, sugar levels)**
- **Blurred vision**
- **Worsening of hand-eye coordination**
- **Mood changes**
- **Worsening of kidney or liver function**
- **Skin rash and itching**
- **Mild hearing loss or ringing in the ears**

<u>Rare but serious</u>

- **Allergic reaction (skin rash, flushing and difficulty breathing)**
- **Low blood pressure, high blood pressure**
- **Dizziness/lightheadedness/headache/fainting**
- **Irregular heart rhythm**
- **Heart attack/cardiac dysfunction**
- **Slowing of heart rate**
- **Inflammation of the colon and/or pancreas**
- **Seizures**

Risks and side effects related to BAY 43-9006 (sorafenib) include the following:

<u>Likely</u>

- **High blood pressure**
- **Fatigue**
- **Fever**
- **Chills**
- **Weight loss**
- **Sores in your mouth**

- **Skin condition which shows up as smooth white spots on various parts of your body (like acne)**
- **Nail changes**
- **Skin rash, itchy skin, dry skin, and skin peeling**
- **Redness, dryness and sometimes pain of the palms and soles**
- **Loss of appetite**
- **Nausea, vomiting and diarrhea**
- **Dehydration**
- **Infection**
- **Inflammation of the pancreas not causing symptoms but showing up as changes in blood enzyme levels (mild pancreatitis)**
- **High blood sugars**
- **Abdominal (stomach) pain/cramping**
- **Joint pain and muscle pain**
- **Flu-like syndrome**

Less Likely

- **Allergic reactions**
- **Hair loss**
- **Fluid build-up in the abdomen and lungs**
- **Constipation**
- **Pain with swallowing solids and liquids**
- **Gas**
- **Heartburn**
- **Low white blood cell count which may cause you to get an infection**
- **Nerve pain and numbness**
- **Low oxygen in your blood**
- **Inflammation of your lungs**
- **Non-life threatening skin tumors**
- **Decrease of phosphates in the blood**
- **Sudden reddening of the face and/or neck** *(3/31/06)*
- **Decrease in the protein in red blood cells, responsible for carrying oxygen throughout the body** *(3/31/06)*
- **Decrease in the total number of white blood cells** *(3/31/06)*
- **Decrease in a type of white blood cells** *(3/31/06)*
- **Decrease in the number of blood cells that help the blood to clot** *(3/31/06)*

Rare but serious

- **Liver damage**
- **Inflammation of the pancreas causing abdominal pain and possibly damage to internal organs (moderate to severe pancreatitis)**
- **Bleeding in your urinary tract or bowels**
- **Collapse of your lungs**
- **Kidney failure**
- **Tear in stomach/intestines that may be painful and possibly fatal** *(added 7/6/06)*

Reproductive risks: You should not become pregnant or father a baby while on this study because the drugs in this study can affect an unborn baby. Women should not breastfeed a baby while on this study. It is important you understand that you need to use birth control

while on this study. Check with your study doctor about what kind of birth control methods to use and how long to use them. Some methods might not be approved for use in this study.

For more information about risks and side effects, ask your study doctor.

Are there benefits to taking part in the study?

Taking part in this study may or may not make your health better. While doctors hope carboplatin, paclitaxel and BAY 43-9006 (sorafenib) will be more useful against cancer compared to the usual treatment, there is no proof of this yet. We do know that the information from this study will help doctors learn more about carboplatin, paclitaxel and BAY 43-9006 (sorafenib) as a treatment for cancer. This information could help future cancer patients.

What other choices do I have if I do not take part in this study?

Your other choices may include:

- **Getting treatment or care for your cancer without being in a study**
- **Taking part in another study**
- **Getting no treatment**
- **Getting comfort care, also called palliative care. This type of care helps reduce pain, tiredness, appetite problems and other problems caused by the cancer. It does not treat the cancer directly, but instead tries to improve how you feel and keep you as active and comfortable as possible.**

Talk to your doctor about your choices before you decide if you will take part in this study.

Will my medical information be kept private?

We will do our best to make sure that the personal information in your medical record will be kept private. However, we cannot guarantee total privacy. Your personal information may be given out if required by law. If information from this study is published or presented at scientific meetings, your name and other personal information will not be used.

Organizations that may look at and/or copy your medical records for research, quality assurance, and data analysis include:

- The Southwest Oncology Group
- The National Cancer Institute (NCI) and other government agencies, like the Food and Drug Administration (FDA), involved in keeping research safe for people
- Qualified representatives of the drug manufacturer/supplier (Bayer Pharmaceuticals and Onyx Pharmaceuticals)

[Note to Local Investigators: The NCI has recommended that HIPAA regulations be addressed by the local institution. The regulations may or may not be included in the informed consent form depending on local institutional policy.]

What are the costs of taking part in this study?

You and/or your health plan/ insurance company will need to pay for some or all of the costs of treating your cancer in this study. Some health plans will not pay these costs for people taking part in studies. Check with your health plan or insurance company to find out what they will pay for. Taking part in this study may or may not cost your insurance company more than the cost of getting regular cancer treatment. *(sentence deleted 6/01/06)*

Administration of the drug will be *(provided free of charge/charged in the usual way).* The parts of the research consisting of keeping research records will be paid by those organizing and conducting the research. The research requires that you receive certain standard medical tests and examinations. These standard tests and examinations will be *(charged in the usual way/provided at a reduced rate). (local institutions must choose the option that best fits the hospital's situation)*

The Division of Cancer Treatment and Diagnosis, NCI, will provide you with the NCI-sponsored/supplied agent, BAY 43-9006 (sorafenib), free of charge, for all participants. If the drug becomes commercially available for this indication, there is a remote possibility that you may be asked to purchase subsequent supplies. Your physician will discuss this with you should this situation arise.

Carboplatin and paclitaxel are commercially available.

You will not be paid for taking part in this study.

For more information on clinical trials and insurance coverage, you can visit the National Cancer Institute's Web site at http://cancer.gov/clinicaltrials/understanding/insurance-coverage . You can print a copy of the "Clinical Trials and Insurance Coverage" information from this Web site.

Another way to get the information is to call 1-800-4-CANCER (1-800-422-6237) and ask them to send you a free copy.

What happens if I am injured because I took part in this study?

It is important that you tell your study doctor, ________________ *[investigator's name(s)],* if you feel that you have been injured because of taking part in this study. You can tell the doctor in person or call him/her at ________________ *[telephone number].*

You will get medical treatment if you are injured as a result of taking part in this study. You and/or your health plan will be charged for this treatment. The study will not pay for medical treatment.

What are my rights if I take part in this study?

Taking part in this study is your choice. You may choose either to take part or not to take part in the study. If you decide to take part in this study, you may leave the study at any time. No matter what decision you make, there will be no penalty to you and you will not lose any of your regular benefits. Leaving the study will not affect your medical care. You can still get your medical care from our institution.

We will tell you about new information or changes in the study that may affect your health or your willingness to continue in the study.

In the case of injury resulting from this study, you do not lose any of your legal rights to seek payment by signing this form.

Who can answer my questions about the study?

You can talk to your study doctor about any questions or concerns you have about this study. Contact your study doctor __________________ *[name(s)]* at __________________ *[telephone number].*

For questions about your rights while taking part in this study, call the ________________________ *[name of center]* Institutional Review Board (a group of people who review the research to protect your rights) at __________________ *(telephone number). [Note to Local Investigator: Contact information for patient representatives or other individuals in a local institution who are not on the IRB or research team but take calls regarding clinical trial questions can be listed here.]*

Please note: This section of the informed consent form is about additional research studies that are being done with people who are taking part in the main study. You may take part in these additional studies if you want to. You can still be a part of the main study even if you say 'no' to taking part in any of these additional studies.

You can say "yes" or "no" to each of the following studies. Please mark your choice for each study.

Specimen Submission

Tissue that was taken during your diagnostic biopsy will be sent to a special laboratory to study tumor growth patterns. *(sentence deleted 6/01/06)*

Additionally, blood specimens (approximated 6 teaspoons total) will be collected before treatment and before Week 3, and urine specimen will be collected before treatment and before Week 3 of treatment to measure proteins important in the anticancer activity of BAY 43-9006 (sorafenib).

The results of the testing will not be given to your or your doctor. Although the results will not affect your treatment, the tests may help future patients. Reports about research done with your specimens will not be put in your health records. Results from these tests may be published, but you will not be identified in these publications.

My archived tumor tissue specimens may be used for the special testing described as part of the study.

Yes _______________ No ________________

My urine and blood specimens may be used for the special testing described as part of the study.

Yes _______________ No ________________

Future Contact

I agree to allow my study doctor, or someone approved by my study doctor, to contact me regarding future research involving my participation in this study.

Yes **No**

Consent Form for Storage and Use of Specimens for Future, Unspecified Research

About Using Specimens for Research

You have had a biopsy (or surgery) to see if you have cancer. Your doctor will remove some body tissue to do some tests. The results of these tests will be given to you by your doctor and will be used to plan your care. *(sentence deleted 6/01/06)* Additionally, blood and urine specimens may be taken.

If you agree to their submission, your specimens will be kept at the following location:

Cecilia M. Fenoglio-Preiser, M.D.
University of Cincinnati
231 Albert Sabin Way
PO Box 670529
Cincinnati, OH 45267-0529
Phone: 513/558-4500

We would like to keep some of the tissue, blood and urine that are left over for future research. If you agree, these specimens will be kept and may be used in research to learn more about cancer and other diseases. Please read the information sheet called "How are Specimens Used for Research" to learn more about tissue research.

The research that may be done with your specimens is not designed specifically to help you. It might help people who have cancer and other diseases in the future.

Reports about research done with your specimens will not be given to you or your doctor. These reports will not be put in your health record. The research will not have an effect on your care.

Things to Think About

The choice to let us keep the left over specimens for future research is up to you. No matter what you decide to do, it will not affect your care.

If you decide now that your specimens can be kept for research, you can change your mind at any time. Just contact us and let us know that you do not want us to use your specimens. Then any specimens that remain will no longer be used for research.

In the future, people who do research may need to know more about your health. While the Southwest Oncology Group may give them reports about your health, it will not give them your name, address, phone number, or any other information that will let the researchers know who you are.

Sometimes specimens are used for genetic research (about diseases that are passed on in families). Even if your tissue is used for this kind of research, the results will not be put in your health records.

Your specimens will be used only for research and will not be sold. The research done with your specimens may help to develop new products in the future.

Benefits

The benefits of research using specimens include learning more about what causes cancer and other diseases, how to prevent them, and how to treat them.

Risks

The greatest risk to you is the release of information from your health records. We will do our best to make sure that your personal information will be kept private. The chance that this information will be given to someone else is very small.

Making Your Choice

Please read each sentence below and think about your choice. After reading each sentence, circle "Yes" or "No." If you have any questions, please talk to your doctor or nurse, or call our research review board at IRB's phone number.

No matter what you decide to do, it will not affect your care.

1. **My specimens may be kept for use in research to learn about, prevent, treat or cure cancer.**

   Yes **No**

2. **My specimens may be kept for use in research about other health problems (for example: diabetes, Alzheimer's disease, or heart disease).**

   Yes **No**

3. **Someone may contact me in the future to ask me to allow other uses of my specimens.**

   Yes **No**

If you decide to withdraw your specimens from a Southwest Oncology Group Specimen Repository in the future, a written withdrawal of consent should be submitted through your treating physician to the Southwest Oncology Group Operations Office. Please designate in the written withdrawal whether you would prefer to have the specimens destroyed or returned to the treating physician.

Where can I get more information?

You may call the National Cancer Institute's Cancer Information Service at:

1-800-4-CANCER (1-800-422-6237) or TTY: 1-800-332-8615

You may also visit the NCI Web site at http://cancer.gov/

- For NCI's clinical trials information, go to: http://cancer.gov/clinicaltrials/
- For NCI's general information about cancer, go to http://cancer.gov/cancerinfo/

You will get a copy of this form. If you want more information about this study, ask your study doctor.

Signature

I have been given a copy of all _____ *[insert total of number of pages]* pages of this form. I have read it or it has been read to me. I understand the information and have had my questions answered. I agree to take part in this study.

Participant _________________________________

Date ______________________________________

Specimen Consent Supplemental Sheets

How are Specimens Used for Research?

Where do specimens come from?

A specimen may be from a blood sample or from bone marrow, skin, toenails or other body materials. People who are trained to handle specimens and protect donors' rights make sure that the highest standards of quality control are followed by the Southwest Oncology Group. Your doctor does not work for the Southwest Oncology Group, but has agreed to help collect specimens from many patients. Many doctors across the country are helping in the same way.

Why do people do research with specimens?

Research with specimens can help to find out more about what causes cancer, how to prevent it, how to treat it, and how to cure it. Research using specimens can also answer other health questions. Some of these include finding the causes of diabetes and heart disease, or finding genetic links to Alzheimer's.

What type of research will be done with my specimen?

Many different kinds of studies use specimens. Some researchers may develop new tests to find diseases. Others may develop new ways to treat or even cure diseases. In the future, some of the research may help to develop new products, such as tests and drugs. Some research looks at diseases that are passed on in families (called genetic research). Research done with your specimen may look for genetic causes and signs of disease.

How do researchers get the specimen?

Researchers from universities, hospitals, and other health organizations conduct research using specimens. They contact the Southwest Oncology Group and request samples for their studies. The Southwest Oncology Group reviews the way that these studies will be done, and decides if any of the samples can be used. The Southwest Oncology Group gets the specimen and information about you from your hospital, and sends the specimen samples and some information about you to the researcher. The Southwest Oncology Group will not send your name, address, phone number, social security number or any other identifying information to the researcher.

Will I find out the results of the research using my specimen?

You will not receive the results of research done with your specimen. This is because research can take a long time and must use specimen samples from many people before results are known. Results from research using your specimen may not be ready for many years and will not affect your care right now, but they may be helpful to people like you in the future.

Why do you need information from my health records?

In order to do research with your specimen, researchers may need to know some things about you. (For example: Are you male or female? What is your race or ethnic group? How old are you? Have you ever smoked?) This helps researchers answer questions about diseases. The information that will be given to the researcher may include your age, sex, race, diagnosis, treatments and family history. This information is collected by your hospital from your health record and sent to the Southwest Oncology Group. If more information is needed, the Southwest Oncology Group will send it to the researcher.

Will my name be attached to the records that are given to the researcher?

No. Your name, address, phone number and anything else that could identify you will be removed before they go the researcher. The researcher will not know who you are.

How could the records be used in ways that might be harmful to me?

Sometimes, health records have been used against patients and their families. For example, insurance companies may deny a patient insurance or employers may not hire someone with a certain illness (such as AIDS or cancer). The results of genetic research may not apply only to you, but to your family members too. For disease caused by gene changes, the information in one person's health record could be used against family members.

How am I protected?

The Southwest Oncology Group is in charge of making sure that information about you is kept private. The Southwest Oncology Group will take careful steps to prevent misuse of records. Your name, address, phone number and any other identifying information will be taken off anything associated with your specimen before it is given to the researcher. This would make it very difficult for any research results to be linked to you or your family. Also, people outside the research process will not have access to results about any one person which will help to protect your privacy.

What if I have more questions?

If you have any questions, please talk to your doctor or nurse, or call our research review board at (Insert IRB's Phone Number).

Southwest Oncology Group Data Operations Center
C/o Cancer Research And Biostatistics
1730 Minor Ave Suite 1900
Seattle, WA 98101-1468
Patient Registration:
via WebReg at: http://swog.org
(24 hours a day, 7 days a week, excluding downtimes for maintenance)
or call 206-652-2267 (Mon-Fri, 6:30am-4:00pm Pacific Time, excluding holidays)

Southwest Oncology Group Operations Office
14980 Omicron Drive
San Antonio, TX 78245-3217
Phone: (210) 677-8808
Fax: (210) 677-0006

Southwest Oncology Group Registration Form

SWOG Study No. S0512 | **Registration Step** 1 | **Assigned Treatment Arm** 1 | **Activation Date: March 15, 2006** | **Last Amended Date:**

Phase II Evaluation of BAY 43-9006 (NSC-724772) in Combination with Carboplatin and Paclitaxel in Patients with Metastatic Uveal Melanoma

Patient's Name ______________________

SWOG Patient ID ☐☐☐☐☐☐

Other Group Patient Number ☐☐☐☐☐☐☐☐☐☐

Participating Group/ Protocol Number ______________________

INSTRUCTIONS: All of the information on this Registration Form and the Protocol Eligibility Section must be answered appropriately for a patient to be considered eligible for registration. This Registration Form must be entirely filled out and referred to during the registration. **Do NOT submit this form as part of the patient data.**

Caller's SWOG Roster ID ☐☐☐☐☐ **IRB Approval Date** ☐☐ / ☐☐ / ☐☐☐☐ **Projected Start Date of Treatment** ☐☐ / ☐☐ / ☐☐☐☐ **Date Informed Consent Signed** ☐☐ / ☐☐ / ☐☐☐☐

SWOG Investigator Number ☐☐☐☐☐ **OR** **Other Group Investigator Name and Number** ______________ / ________

SWOG Treating Institution Number ☐☐☐☐☐ **OR** **Other Group Institution Name and Number** ______________ / ________

Date HIPAA Authorization signed: ☐☐ / ☐☐ / ☐☐☐☐ ***(Not required for non-American sites)***

Please indicate how the patient answered the following questions on the consent form:

1. **My specimens may be kept for use in research to learn about, prevent, treat or cure cancer.** ☐ Yes ☐ No
2. **My specimens may be kept for use in research about other health problems (for example: diabetes, Alzheimer's disease, or heart disease).** ☐ Yes ☐ No
3. **Someone may contact me in the future to ask me to allow other uses of my tissue.** ☐ Yes ☐ No
4. **I agree to allow my study doctor, or someone approved by my study doctor, to contact me regarding future research involving my participation in this study.** ☐ Yes ☐ No

Patient's Date of Birth: ☐☐ / ☐☐ / ☐☐☐☐

Patient Gender: ☐ Female ☐ Male **Method of Payment:** ☐☐ **Patient's Ethnicity:** ☐

Patient's Race (select all that apply):

☐ White ☐ Native Hawaiian or Other Pacific Islander ☐ American Indian or Alaska Native
☐ Black or African American ☐ Asian ☐ Unknown

If a U.S. resident:

Patient Social Security Number: ☐☐☐ - ☐☐ - ☐☐☐☐ **Patient's ZIP Code:** ☐☐☐☐☐

Country of Residence (if not USA): ______________________

If a resident of Canada:

Social Insurance Number: ☐☐☐ - ☐☐☐ - ☐☐☐ **Postal Code:** ☐☐☐ - ☐☐☐

63366

3/15/2006

Southwest Oncology Group Registration Form Code Sheet 12/8/2004

Race code definitions:

White or Caucasian: a person having origins in any of the original peoples of Europe, Middle East, or North Africa

Black or African American: a person having origins in any of the black racial groups of Africa.

Native Hawaiian or Other Pacific Islander: a person having origins in any of the original peoples of Hawaii, Guam, Samoa and other Pacific islands.

Asian: a person having origins in any of the original peoples of the Far East, Southeast Asia, or the Indian subcontinent. Including, for example, Cambodia, China, India, Japan, Korea, Malaysia, Pakistan, the Philippine Islands, Thailand and Vietnam.

American Indian or Alaska Native: a person having origins in any of the original peoples of North, Central or South America, and who maintains tribal affiliations or community attachment.

Ethnicity (Spanish/Hispanic Origin) codes:

0 - Unknown
1 - No (not Spanish)
2 - Yes, Mexican
3 - Yes, Puerto Rican
4 - Yes, Cuban
5 - Yes, Central American
6 - Yes, South American
7 - Yes, Other
8 - Yes, NOS

Method of Payment codes:

01 - Private
02 - Medicare
03 - Medicare and Private
04 - Medicaid
05 - Medicaid and Medicare
07 - No insurance (self-pay)
08 - No insurance (no means)
09 - Other, specify at registration
10 - Unknown
11 - Veterans Admin
12 - Military

Other Group codes for use in the Web Registration program:

9977 - ACOSOG
9982 - CALGB
9976 - CTSU
9995 - ECOG
9979 - EPP
9984 - GOG
9996 - NCCTG
9981 - NCIC
9983 - NSABP
9997 - RTOG

SOUTHWEST ONCOLOGY GROUP
S0512 PRESTUDY FORM

SWOG Patient ID ☐☐☐☐☐☐ **SWOG Study No.** S 0 5 1 2 **Registration Step** 1

Patient Initials __________ (L, F M)

Institution / Affiliate ______________________ Physician ______________________

Participating Group: Group Name/Study No./Patient ID ________ / ________ / ________

Instructions: Submit this form within 14 days of registration. All dates are **MONTH, DAY, YEAR**. Explain any blank fields or blank dates in the **Comments** section. Place an X in appropriate boxes. Circle **AMENDED** items in red and write **AMENDED** across top of form.

ELIGIBILITY VERIFICATION:

Each of the fields below corresponds to a criterion in Section 5 and must be completed for patient to be eligible.

PATIENT AND DISEASE DESCRIPTION

Performance status: ☐

Bone metastases? ☐ Yes ☐ No

If Yes, date of bone scan: ☐☐ / ☐☐ / ☐☐☐☐

Major surgery? ☐ Yes ☐ No

If Yes, date of last major surgery: ☐☐ / ☐☐ / ☐☐☐☐

Traumatic injury? ☐ Yes ☐ No

If Yes, date of last traumatic injury: ☐☐ / ☐☐ / ☐☐☐☐

LABORATORY VALUES

Document values in units listed

Hematologic: **Date collected:**

**ANC**	☐,☐☐☐ / mcL		☐☐ / ☐☐ / ☐☐☐☐
**Platelets**	☐☐☐,☐☐☐ / mcL		☐☐ / ☐☐ / ☐☐☐☐

Hepatic: **Liver metastases?** ☐ Yes ☐ No **Date collected:**

**Total bilirubin**	☐☐☐.☐ mg/dL	**IULN** ☐☐☐.☐ mg/dL	☐☐ / ☐☐ / ☐☐☐☐
**Transaminase (select one)** ☐ SGOT ☐ SGPT	☐☐☐ U/L	**IULN** ☐☐☐ U/L	☐☐ / ☐☐ / ☐☐☐☐

Renal: **Date collected:**

**Serum creatinine**	☐☐.☐☐ mg/dL	**IULN** ☐☐.☐☐ mg/dL	☐☐ / ☐☐ / ☐☐☐☐

Coagulation: **Is the patient on a full-dose anticoagulant (e.g. warfarin)?** ☐ Yes ☐ No

If Yes: **Date collected:**

**INR** ☐.☐	**ILLN** ☐.☐	**IULN** ☐.☐		☐☐ / ☐☐ / ☐☐☐☐

34876

 continued on next page

SWOG Patient ID ☐☐☐☐☐☐ **SWOG Study No.** S 0 5 1 2 **Registration Step** 1

Patient Initials__________ (L, F M)

ADDITIONAL PRESTUDY DATA:

PATIENT CHARACTERISTICS

Height: ☐☐☐ cm

DISEASE DESCRIPTION

Date of First Pathologic Diagnosis of Primary Uveal Melanoma: ☐☐ / ☐☐ / ☐☐☐☐

Is there local/regional disease at registration? ☐ Yes ☐ No

Date of First Diagnosis of Distant Metastatic Disease: ☐☐ / ☐☐ / ☐☐☐☐

(Distant metastatic involvement is defined as the presence of one or more metastases beyond the regional lymph node drainage.)

Distant Metastatic Involvement at Registration *(select all that apply)*:

☐ Lung ☐ Liver ☐ Brain/CNS ☐ Bone

☐ Lymph Node, Skin, or Soft Tissue (specify): ____________________

☐ Other visceral (specify): ____________________

☐ Other non-visceral (specify): ____________________

Elevated serum LDH at registration? ☐ Yes ☐ No

Serum LDH: ☐☐☐.☐ U/L **Serum LDH IULN:** ☐☐☐.☐ U/L

continued on next page

SWOG Patient ID ☐☐☐☐☐☐ **SWOG Study No.** S0512 **Registration Step** 1

Patient Initials__________ (L, F M)

PRIOR TREATMENT RELATED TO THIS CANCER:

PRIOR TREATMENT RELATED TO THIS CANCER

Prior Therapy Type(s) *(select ALL that apply)*:

Note: For prior combination regimens, e.g. biochemotherapy, mark boxes for both chemotherapy and biologic therapy below, and list all agents in the chemotherapy section.

☐ Bone marrow transplant

☐ Chemotherapy; if Yes:

Adjuvant:

☐ Multi-agent Specify: ______________________

☐ Single agent Specify: ______________________

For advanced disease:

☐ Multi-agent Specify: ______________________

☐ Single agent Specify: ______________________

Total number of prior chemotherapy regimens **(adjuvant and advanced)**: ☐☐

☐ Limb perfusion therapy

☐ Gene therapy

☐ Hormonal therapy; if Yes, select all that apply: ☐ Adjuvant ☐ For advanced disease

☐ Biologic/Immunotherapy; if Yes:

Adjuvant:

☐ Multi-agent Specify: ______________________

☐ Single agent Specify: ______________________

For advanced disease:

☐ Multi-agent Specify: ______________________

☐ Single agent Specify: ______________________

☐ Radiotherapy; if Yes, select one:

☐ Extensive Radiation ($\geq$50% of the body)

☐ Limited Radiation (< 50% of the body)

☐ Not otherwise specified

☐ Surgery; if Yes, select all that apply:

☐ Resection of primary tumor

☐ Resection of regional metastases (regional nodes, satellite/in-transit metastases)

☐ Resection of distant metastases

☐ Other type of prior treatment; if Yes, specify: ______________________

Comments:

SOUTHWEST ONCOLOGY GROUP
BASELINE TUMOR ASSESSMENT FORM

SWOG Patient ID ☐☐☐☐☐☐ **SWOG Study No.** S☐☐☐☐ **Registration Step** ☐

Patient Initials ____________ (L, F M)

Institution / Affiliate ______________________________ Physician ________________________________

Participating Group: Group Name/Study No./Patient ID _______________ / ______________ / ______________

Instructions: Record the requested information for all target lesions and all sites of other disease. Please refer to section 10.0 of the protocol for definitions. Choose all measurable lesions up to a maximum of 5 lesions per organ and 10 lesions in total to follow as target lesions. Record any remaining measurable lesions and all non-measurable disease as non-target disease.
All dates are **MONTH, DAY, YEAR**. Circle **AMENDED** items in red.
The same test procedures used for baseline disease assessment must be used for all required subsequent disease assessments.

TARGET LESIONS	Longest Diameter (cm)	Assessment Type*	Assessment Date
L1 ______	☐☐.☐	☐☐	☐☐ / ☐☐ / ☐☐☐☐
L2 ______	☐☐.☐	☐☐	☐☐ / ☐☐ / ☐☐☐☐
L3 ______	☐☐.☐	☐☐	☐☐ / ☐☐ / ☐☐☐☐
L4 ______	☐☐.☐	☐☐	☐☐ / ☐☐ / ☐☐☐☐
L5 ______	☐☐.☐	☐☐	☐☐ / ☐☐ / ☐☐☐☐
L6 ______	☐☐.☐	☐☐	☐☐ / ☐☐ / ☐☐☐☐
L7 ______	☐☐.☐	☐☐	☐☐ / ☐☐ / ☐☐☐☐
L8 ______	☐☐.☐	☐☐	☐☐ / ☐☐ / ☐☐☐☐
L9 ______	☐☐.☐	☐☐	☐☐ / ☐☐ / ☐☐☐☐
L10 ______	☐☐.☐	☐☐	☐☐ / ☐☐ / ☐☐☐☐

NON-TARGET DISEASE	Extent	Assessment Type*	Assessment Date
S1 ______	______	☐☐	☐☐ / ☐☐ / ☐☐☐☐
S2 ______	______	☐☐	☐☐ / ☐☐ / ☐☐☐☐
S3 ______	______	☐☐	☐☐ / ☐☐ / ☐☐☐☐
S4 ______	______	☐☐	☐☐ / ☐☐ / ☐☐☐☐
S5 ______	______	☐☐	☐☐ / ☐☐ / ☐☐☐☐

List all **negative** diagnostic tests/studies used to evaluate patient for malignancy.

Tests/studies	Assessment Date	Tests/studies	Assessment Date
______	☐☐ / ☐☐ / ☐☐☐☐	______	☐☐ / ☐☐ / ☐☐☐☐
______	☐☐ / ☐☐ / ☐☐☐☐	______	☐☐ / ☐☐ / ☐☐☐☐

*** Assessment Types:**

01-Palpation	12-CT scan	18-Cystoscopy
02-Visualization	13-MRI scan	20-Histologic confirmation
03-Colposcopy	14-Radioisotope scan	21-Cytologic confirmation
05-Endoscopy	15-Ultrasound	99-Other (specify in Comments and indicate lesion number)
10-Plain film/X-ray without contrast	16-PET scan	
11-Plain film/X-ray with contrast	17-Spiral CT scan	

Comments:

SOUTHWEST ONCOLOGY GROUP
S0512 BASELINE ABNORMALITIES FORM

SWOG Patient ID ☐☐☐☐☐☐ **SWOG Study No.** S 0 5 1 2 **Registration Step** 1

Patient Initials ____________ (L, F M)

Institution/Affiliate ______________________ Physician ______________________

Participating Group: Group Name/Study No./Patient ID ____________ / ____________ / ____________

Instructions: Using CTCAE 3.0 Grade definitions, please grade any abnormalities or conditions present **PRIOR** to protocol treatment. Circle **AMENDED** items in red and write **AMENDED** across top of form.

	CTC Adverse Event Term (Pre-treatment)	CTCAE (3.0) Grade (1 - 4)		CTC Adverse Event Term (Pre-treatment)	CTCAE (3.0) Grade (1 - 4)		CTC Adverse Event Term (Pre-treatment)	CTCAE (3.0) Grade (1 - 4)
IM00	Allergic reaction/ hypersensitivity	☐	GI01	Anorexia	☐	ME05	Bilirubin	☐
HE20	Hemoglobin	☐	GI91	Ascites	☐	ME07	GGT	☐
HE00	Leukocytes	☐	GI62	Colitis	☐	ME31	Glucose, serum-high	☐
HE30	Neutrophils/granulocytes	☐	GI30	Constipation	☐	ME82	Lipase	☐
HE10	Platelets	☐	GI23	Dehydration	☐	NR50	Neuropathy: motor	☐
CA01	Cardiac-ischemia/infarction	☐	GI20	Diarrhea	☐	NR60	Neuropathy: sensory	☐
CA02	Cardiopulmonary arrest, cause unknown	☐	GI66	Dysphagia	☐	EY42	Vision-blurred vision	☐
CA50	Hypertension	☐	GI92	Flatulence	☐	EY41	Vision-flashing lights/floaters	☐
CA51	Hypotension	☐	GI02	Heartburn/dyspepsia	☐		Pain	
FL40	Fatigue	☐		Mucositis/stomatitis (functional/symptomatic)		PAI01	Abdomen NOS	☐
FL01	Fever	☐	GIM52	Pharynx	☐	PAN37	Head/headache	☐
FL20	Insomnia	☐	GI00	Nausea	☐	PAM11	Joint	☐
FL10	Rigors/chills	☐	GI43	Taste alteration	☐	PAM14	Muscle	☐
FL51	Weight loss	☐	GI10	Vomiting	☐	LU00	Dyspnea	☐
SK80	Dry skin	☐	LI04	Pancreatitis	☐	LU10	Hypoxia	☐
SK01	Flushing	☐	IN30	Febrile neutropenia	☐	LU51	Pleural effusion	☐
SK90	Hair loss/alopecia	☐		Infection with Grade 3 or 4 neutrophils		LU50	Pneumonitis/pulmonary infiltrates	☐
SK41	Hypopigmentation	☐	ICL05	Lung	☐	LU70	Pneumothorax	☐
SK60	Nail changes	☐	ME03	ALT, SGPT	☐	GU53	Renal failure	☐
SK16	Pruritus/itching	☐	ME04	AST, SGOT	☐		**CTC Adverse Event Term, Other (pre-treatment)** *(specify using CTCAE 3.0 terminology):*	
SK11	Rash/desquamation	☐	ME09	Albumin, serum-low	☐		______________________	☐
SK22	Rash: acne/acneiform	☐	ME02	Alkaline phosphatase	☐		______________________	☐
SK13	Rash: hand-foot skin reaction	☐	ME40	Amylase	☐		______________________	☐

Comments: (Please explain any "other" baseline abnormalities reported above, e.g. Pain-other)

SOUTHWEST ONCOLOGY GROUP
S0512 CYCLE-SPECIFIC TREATMENT FORM

SWOG Patient ID ☐☐☐☐☐☐ **SWOG Study No.** S 0 5 1 2 **Registration Step** 1

Patient Initials ________ (L, F M) **Current Cycle Number** ☐☐ *(see instructions)*

Institution/Affiliate ______________________ Physician ______________________

Participating Group: Group Name/Study No./Patient ID ____________ / ____________ / ____________

Instructions: Please complete this form after each cycle (*1 cycle = 21 days*). All dates are **MONTH, DAY, YEAR**. Explain any blank dates or fields in the **Comments** section. Place an X in appropriate boxes. Circle **AMENDED** items in red and write **AMENDED** across top of form.

STATUS

Date of Last Contact or Death: ☐☐ / ☐☐ / ☐☐☐☐ **Vital Status:** ☐ Alive ☐ Dead *(submit Notice of Death)*

Has the patient progressed per the definition in Section 10.0 of the protocol? ☐ No ☐ Yes
(If Yes, submit Follow-Up Tumor Assessment Form and Off Treatment Notice)

TREATMENT FOR THIS CYCLE

Cycle start date: ☐☐ / ☐☐ / ☐☐☐☐ **Weight (first day this cycle):** ☐☐☐.☐ kg

Date of last treatment for this cycle: ☐☐ / ☐☐ / ☐☐☐☐ **BSA (first day this cycle):** ☐.☐☐ m^2

Were there any dose modifications or additions/omissions to protocol treatment?

- ☐ No
- ☐ Yes, planned (per protocol guidelines), specify in comments
- ☐ Yes, unplanned (not per protocol guidelines), specify in comments

Serum creatinine (for carboplatin dosing): ☐☐.☐

Calculated creatinine clearance (using Cockroft-Gault Formula - see sec 7.3 or appendix 19.4): ☐☐☐

**BAY 43-9006**	**Paclitaxel**	**Carboplatin**
☐☐☐☐☐ mg	☐☐☐.☐☐ mg	☐☐☐☐ mg
(1 pill = 200 mg)		

Comments:

SOUTHWEST ONCOLOGY GROUP
S0512 CYCLE-SPECIFIC ADVERSE EVENT FORM

SWOG Patient ID ☐☐☐☐☐☐ **SWOG Study No.** S 0 5 1 2 **Registration Step** 1

Patient Initials ________ (L, F M) **Current Cycle Number** ☐☐ *(see instructions)*

Institution/Affiliate ______________________ Physician ______________________

Participating Group: Group Name/Study No./Patient ID ____________ / ____________ / ____________

Instructions: Please complete this form after each cycle (*1 cycle = 21 days*). Report adverse events occurring up until the next cycle of treatment begins. Document the worst Grade seen during the reporting period. Do not code a condition existing prior to registration as an adverse event unless it worsens. Category lists may not include all adverse events from that category. Record any observed adverse events not listed on the blank lines at the end. All dates are MONTH, DAY, YEAR. Explain any blank dates or fields in the **Comments** section. Place an X in appropriate boxes. Circle **AMENDED** items in red and write **AMENDED** across top of form.

ADVERSE EVENTS

Reporting period start date: ☐☐ / ☐☐ / ☐☐☐☐ *(Day 1 of this Cycle)*

Reporting period end date: ☐☐ / ☐☐ / ☐☐☐☐ *(Day one of next cycle. If final cycle, date of first visit or contact after resolution of acute adverse events.)*

Were adverse events assessed during this time period?

☐ No ☐ Yes, but no reportable adverse events occurred

☐ Yes, and reportable adverse events occurred (report below)

	CTC Adverse Event Term	CTCAE (3.0) Grade (1 - 5)	CTC Adverse Event Attribution Code*	Adverse Event Status Code**
IM00	Allergic reaction/ hypersensitivity	☐	☐	☐
HE20	Hemoglobin	☐	☐	☐
HE00	Leukocytes	☐	☐	☐
HE30	Neutrophils/granulocytes	☐	☐	☐
HE10	Platelets	☐	☐	☐
CA01	Cardiac-ischemia/ infarction	☐	☐	☐
CA02	Cardiopulmonary arrest, cause unknown	☐	☐	☐
CA50	Hypertension	☐	☐	☐
CA51	Hypotension	☐	☐	☐
FL40	Fatigue	☐	☐	☐
FL01	Fever	☐	☐	☐
FL20	Insomnia	☐	☐	☐
FL10	Rigors/chills	☐	☐	☐
FL51	Weight loss	☐	☐	☐
SK80	Dry skin	☐	☐	☐
SK01	Flushing	☐	☐	☐
SK90	Hair loss/alopecia	☐	☐	☐
SK41	Hypopigmentation	☐	☐	☐
SK60	Nail changes	☐	☐	☐
SK16	Pruritus/itching	☐	☐	☐
SK11	Rash/desquamation	☐	☐	☐
SK22	Rash: acne/acneiform	☐	☐	☐
SK13	Rash: hand-foot skin reaction	☐	☐	☐
GI01	Anorexia	☐	☐	☐
GI91	Ascites	☐	☐	☐
GI62	Colitis	☐	☐	☐
GI30	Constipation	☐	☐	☐
GI23	Dehydration	☐	☐	☐
GI20	Diarrhea	☐	☐	☐
GI66	Dysphagia	☐	☐	☐
GI92	Flatulence	☐	☐	☐
GI02	Heartburn/dyspepsia	☐	☐	☐

*** Attribution codes:** 1-unrelated 2-unlikely 3-possible 4-probable 5-definite

****Status codes** (since baseline or last cycle): 1-new 2-continues at same or lower grade 3-increased grade OR improved then worsened

continued on next page

SOUTHWEST ONCOLOGY GROUP
S0512 CYCLE-SPECIFIC ADVERSE EVENT FORM

SWOG Patient ID [ ] **SWOG Study No.** S0512 **Registration Step** 1

Patient Initials ________ (L, F M) **Current Cycle Number** [ ]

ADVERSE EVENTS, *continued*

	CTC Adverse Event Term	CTCAE (3.0) Grade (1 - 5)	CTC Adverse Event Attribution Code*	Adverse Event Status Code**
	Mucositis/stomatitis (functional/symptomatic)			
GIM52	Pharynx			
GI00	Nausea			
GI43	Taste alteration			
GI10	Vomiting			
LI04	Pancreatitis			
IN30	Febrile neutropenia			
	Infection with Grade 3 or 4 neutrophils			
ICL05	Lung			
ME03	ALT, SGPT			
ME04	AST, SGOT			
ME09	Albumin, serum-low			
ME02	Alkaline phosphatase			
ME40	Amylase			
ME05	Bilirubin			
ME07	GGT			
ME31	Glucose, serum-high			
ME82	Lipase			
NR50	Neuropathy: motor			

	CTC Adverse Event Term	CTCAE (3.0) Grade (1 - 5)	CTC Adverse Event Attribution Code*	Adverse Event Status Code**
NR60	Neuropathy: sensory			
EY42	Vision-blurred vision			
EY41	Vision-flashing lights/ floaters			
	Pain			
PAI01	Abdomen NOS			
PAN37	Head/headache			
PAM11	Joint			
PAM14	Muscle			
LU00	Dyspnea			
LU10	Hypoxia			
LU51	Pleural effusion			
LU50	Pneumonitis/pulmonary infiltrates			
LU70	Pneumothorax			
GU53	Renal failure			

CTC Adverse Event Term, Other
(specify using CTCAE 3.0 terminology and provide details in comments section)

CTC Adverse Event Term, Other	CTCAE (3.0) Grade (1 - 5)	CTC Adverse Event Attribution Code*	Adverse Event Status Code**
______________			
______________			
______________			

*** Attribution codes:** 1-unrelated 2-unlikely 3-possible 4-probable 5-definite
****Status codes** (since baseline or last cycle)**:** 1-new 2-continues at same or lower grade 3-increased grade OR improved then worsened

Comments: (Please explain any "other" adverse events reported above, e.g. Pain-other)

SOUTHWEST ONCOLOGY GROUP
FOLLOW UP FORM

SWOG Patient ID ☐☐☐☐☐☐ **SWOG Study No.** S☐☐☐☐ **Registration Step** ☐

Patient Initials ___________ (L, F M)

Institution / Affiliate ______________________ Physician ______________________

Participating Group: Group Name/Study No./Patient ID ____________ / ____________ / ____________

Instructions: Please submit at each follow up after completion of treatment until relapse or progression, at time of relapse or progression, and at protocol-specified intervals after relapse or progression. Also submit at time of diagnosis of second primary. All dates are **MONTH, DAY, YEAR**. Answer all questions and explain any blank fields or blank dates in the **Comments** section. Place an X in appropriate boxes. Circle AMENDED items in red.

VITAL STATUS

Vital Status: ☐ Alive ☐ Dead Date of last contact or death: ☐☐ / ☐☐ / ☐☐☐☐

If vital status is Dead, complete and submit Notice of Death form.

DISEASE FOLLOW UP STATUS

Has the patient had a documented clinical assessment for this cancer (since submission of the previous follow-up form)?

☐ No ☐ Yes If Yes, Date of Last Clinical Assessment: ☐☐ / ☐☐ / ☐☐☐☐

NOTICE OF FIRST RELAPSE OR PROGRESSION

Has the patient developed a first relapse or progression that has not been previously reported?

☐ No ☐ Yes If Yes, Date of Relapse or Progression: ☐☐ / ☐☐ / ☐☐☐☐

Site(s) of Relapse or Progression: ______________________

NOTICE OF NEW PRIMARY

Has a new primary cancer or MDS (myelodysplastic syndrome) been diagnosed that has not been previously reported?

☐ No ☐ Yes If Yes, Date of Diagnosis: ☐☐ / ☐☐ / ☐☐☐☐

New Primary Site: ______________________

NON-PROTOCOL TREATMENT

Has the patient received any non-protocol cancer therapy (prior to progression/relapse) not previously reported?

☐ No ☐ Yes If Yes, Date of First Non-Protocol Therapy: ☐☐ / ☐☐ / ☐☐☐☐

Agent Name(s): ______________________

LONG TERM ADVERSE EVENT

Has the patient experienced (prior to treatment for progression or relapse or a second primary, and prior to non-protocol treatment) any severe (grade ≥ 3) long term toxicity that has not been previously reported?

☐ No ☐ Yes If Yes, Adverse Events and Grades: ______________________

Comments:

SOUTHWEST ONCOLOGY GROUP
FOLLOW-UP TUMOR ASSESSMENT FORM

SWOG Patient ID ☐☐☐☐☐☐ **SWOG Study No.** S☐☐☐☐ **Registration Step** ☐

Patient Initials ____________ (L, F M) **Date form completed:** ☐☐ / ☐☐ / ☐☐☐☐

Institution/Affiliate ______________________________ Physician ______________________________

Participating Group: Group Name/Study No./Patient ID ______________ / ______________ / ______________

Instructions: Record the requested information for all target lesions and all sites of other disease. Lesion and site numbers (L1, S1, etc.) should correspond to the same lesions and sites of disease as indicated on the baseline tumor assessment form. If a target lesion was assessed but measurements were not obtainable, complete assessment type and assessment date, and state lesion number and reason measurement was not obtained in **Comments** section on page 2. Record site of any new lesions (since baseline) in the **New Lesions** section. Place an ☒ in appropriate boxes. All dates are **MONTH, DAY, YEAR**.
Circle **AMENDED** items in red.
The same test procedures used for baseline disease assessment must be used for all required subsequent disease assessments.

TARGET LESIONS

		Longest Diameter (cm)	Assessment Type*	Assessment Date
L1	______________	☐☐.☐	☐☐	☐☐ / ☐☐ / ☐☐☐☐
L2	______________	☐☐.☐	☐☐	☐☐ / ☐☐ / ☐☐☐☐
L3	______________	☐☐.☐	☐☐	☐☐ / ☐☐ / ☐☐☐☐
L4	______________	☐☐.☐	☐☐	☐☐ / ☐☐ / ☐☐☐☐
L5	______________	☐☐.☐	☐☐	☐☐ / ☐☐ / ☐☐☐☐
L6	______________	☐☐.☐	☐☐	☐☐ / ☐☐ / ☐☐☐☐
L7	______________	☐☐.☐	☐☐	☐☐ / ☐☐ / ☐☐☐☐
L8	______________	☐☐.☐	☐☐	☐☐ / ☐☐ / ☐☐☐☐
L9	______________	☐☐.☐	☐☐	☐☐ / ☐☐ / ☐☐☐☐
L10	______________	☐☐.☐	☐☐	☐☐ / ☐☐ / ☐☐☐☐

NON-TARGET DISEASE

		Extent	Assessment Type*	Assessment Date
S1	______________	☐ Complete disappearance<br>☐ Present<br>☐ Clear increase (describe in Comments on page 2)<br>☐ Not assessed	☐☐	☐☐ / ☐☐ / ☐☐☐☐
S2	______________	☐ Complete disappearance<br>☐ Present<br>☐ Clear increase (describe in Comments on page 2)<br>☐ Not assessed	☐☐	☐☐ / ☐☐ / ☐☐☐☐

*** Assessment Types:**

01-Palpation	12-CT scan	18-Cystoscopy
02-Visualization	13-MRI scan	20-Histologic confirmation
03-Colposcopy	14-Radioisotope scan	21-Cytologic confirmation
05-Endoscopy	15-Ultrasound	
10-Plain film/X-ray without contrast	16-PET scan	99-Other (specify in Comments on page 2 and indicate lesion number)
11-Plain film/X-ray with contrast	17-Spiral CT scan	

SOUTHWEST ONCOLOGY GROUP
FOLLOW-UP TUMOR ASSESSMENT FORM

SWOG Patient ID ☐☐☐☐☐☐ **SWOG Study No.** S☐☐☐☐ **Registration Step** ☐

Patient Initials ____________ (L, F M) **Date form completed:** ☐☐ / ☐☐ / ☐☐☐☐

NON-TARGET DISEASE, continued

	Extent	Assessment Type*	Assessment Date
**S3** ______________	☐ Complete disappearance<br>☐ Present<br>☐ Clear increase (describe in Comments)<br>☐ Not assessed	☐☐	☐☐ / ☐☐ / ☐☐☐☐
**S4** ______________	☐ Complete disappearance<br>☐ Present<br>☐ Clear increase (describe in Comments)<br>☐ Not assessed	☐☐	☐☐ / ☐☐ / ☐☐☐☐
**S5** ______________	☐ Complete disappearance<br>☐ Present<br>☐ Clear increase (describe in Comments)<br>☐ Not assessed	☐☐	☐☐ / ☐☐ / ☐☐☐☐

NEW LESIONS (Specify Site)

	Assessment Type*	Assessment Date
**S1** ______________	☐☐	☐☐ / ☐☐ / ☐☐☐☐
**S2** ______________	☐☐	☐☐ / ☐☐ / ☐☐☐☐

Symptomatic deterioration: ☐ No ☐ Yes, describe in Comments

Assessment Date ☐☐ / ☐☐ / ☐☐☐☐

*** Assessment Types:**

01-Palpation	12-CT scan	18-Cystoscopy
02-Visualization	13-MRI scan	20-Histologic confirmation
03-Colposcopy	14-Radioisotope scan	21-Cytologic confirmation
05-Endoscopy	15-Ultrasound	
10-Plain film/X-ray without contrast	16-PET scan	99-Other (specify in Comments and indicate lesion number)
11-Plain film/X-ray with contrast	17-Spiral CT scan	

Comments:

SOUTHWEST ONCOLOGY GROUP
OFF TREATMENT NOTICE

SWOG Patient ID ☐☐☐☐☐☐ **SWOG Study No.** S☐☐☐☐ **Registration Step** ☐

Patient Initials ____________ (L, F M)

Institution / Affiliate ________________________ Physician ________________________

Participating Group: Group Name/Study No./Patient ID ____________ / ____________ / ____________

Instructions: For each registration step, submit this form within 2 weeks after completion (or discontinuation) of treatment.

Systemic Therapy: List regimens, start and end dates. For multidrug regimens, do not list individual drugs separately; end date would be the date all drugs in the regimen were discontinued.

Surgery: List type of surgery, and in the "end date" column, the date of surgery.

Radiation: List sites, start and end dates (inclusive of boosts and implants).

All dates are **MONTH, DAY, YEAR**. Explain any blank fields or blank dates in the **Comments** section.
Place an X in appropriate boxes. Circle **AMENDED** items in red.

Treatment Start Date	Treatment End Date	Regimen or Procedure or Site(s)
☐☐ / ☐☐ / ☐☐☐☐	☐☐ / ☐☐ / ☐☐☐☐	________________
☐☐ / ☐☐ / ☐☐☐☐	☐☐ / ☐☐ / ☐☐☐☐	________________
☐☐ / ☐☐ / ☐☐☐☐	☐☐ / ☐☐ / ☐☐☐☐	________________
☐☐ / ☐☐ / ☐☐☐☐	☐☐ / ☐☐ / ☐☐☐☐	________________
☐☐ / ☐☐ / ☐☐☐☐	☐☐ / ☐☐ / ☐☐☐☐	________________

(If more room is needed, please continue on a separate page)

Off Treatment Reason (select one):

- ☐ Treatment completed per protocol criteria
- ☐ Medically required, due to toxicity, specify: ________________
- ☐ Patient refused, due to toxicity, specify: ________________
- ☐ Patient refused, other than toxicity, specify: ________________
- ☐ Progression or relapse. Sites: ________________
- ☐ Death (submit Notice of Death form)
- ☐ Other, specify: ________________

Off Treatment Date

Date of completion, progression, death or decision to discontinue therapy: ☐☐ / ☐☐ / ☐☐☐☐

Will patient receive further treatment?

☐ No ☐ Yes, specify: ________________ ☐ Unknown

Date of Last Contact (or death): ☐☐ / ☐☐ / ☐☐☐☐

Vital Status: ☐ Alive ☐ Dead (submit Notice of Death form)

Comments:

SOUTHWEST ONCOLOGY GROUP
NOTICE OF DEATH

SWOG Patient ID ☐☐☐☐☐☐ **Most Recent SWOG Study No.** S☐☐☐☐

Patient Initials ____________ (L, F M)

Institution / Affiliate ________________________________ Physician ________________________________

Participating Group: Group Name/Study No./Patient ID ______________ / ______________ / ______________

Instructions: Answer all questions and explain any blank fields or blank dates in the **Comments** section.
Place an ☒ in appropriate boxes. Circle **AMENDED** items in red.

Date of Death: ☐☐ / ☐☐ / ☐☐☐☐ (month / day / year)

CAUSES OF DEATH

Any cancer (select one):

☐ No ☐ Primary Cause ☐ Contributory ☐ Possible ☐ Unknown

If cancer was the primary cause or if cancer possibly or definitely contributed to death, and the patient had had multiple tumor types, specify those which were causes of death:

☐ Cancer of most recent SWOG study, specify cancer: ______________________________

☐ Cancer of other SWOG study, specify cancer: ______________________________

☐ Other cancer, specify: ______________________________

Toxicity from disease related treatment (select one):

☐ No ☐ Primary Cause ☐ Contributory ☐ Possible ☐ Unknown

If Primary Cause, Contributory or Possible, specify treatment and toxicity:

Non-cancer and non-treatment related causes (select one):

☐ No ☐ Primary Cause ☐ Contributory ☐ Possible ☐ Unknown

If Primary Cause, Contributory or Possible, specify:

Autopsy? ☐ No ☐ Yes ☐ Unknown

Source(s) of death information:

☐ Autopsy report
☐ Medical record / Death certificate
☐ Physician
☐ Relative or friend
☐ Other, specify: ______________________________

Comments:

19.0 APPENDIX

19.1 Determination of Expedited Adverse Event Reporting Requirements

19.2 Intake Calendar

19.3 Drugs Known to be Metabolized by Selected CYP450 Isoenzymes

19.4 Carboplatin Dosing Worksheet

19.1 Determination of Expedited Adverse Event Reporting Requirements

Adverse event data collection and reporting, which are required as part of every clinical trial, are done to ensure the safety of patients enrolled in the studies as well as those who will enroll in future studies using similar agents. Adverse events are reported in a routine manner at scheduled times during a trial. (Directions for routine reporting are provided in Section 14.0.) Additionally, certain adverse events must be reported in an expedited manner to allow for more timely monitoring of patient safety and care. Expedited adverse event reporting principles and general guidelines follow; specific guidelines for expedited adverse event reporting on this protocol are found in Section 16.0.

Reporting requirements may include the following considerations: 1) whether the patient has received an investigational or commercial agent; 2) the characteristics of the adverse event including the *grade* (severity), the *relationship to the study therapy* (attribution), and the *prior experience* (expectedness) of the adverse event; 3) the Phase (1, 2 or 3) of the trial; and 4) whether or not hospitalization or prolongation of hospitalization was associated with the event.

An investigational agent is a protocol drug administered under an Investigational New Drug Submission (IND). In some instances, the investigational agent may be available commercially, but is actually being tested for indications not included in the approved package label.

Commercial agents are those agents not provided under an IND but obtained instead from a commercial source. The NCI, rather than a commercial distributor, may on some occasions distribute commercial agents for a trial.

When a study includes both investigational and commercial agents, the following rules apply.

- *Concurrent administration*: When an investigational agent(s) is used in combination with a commercial agent(s), the combination is considered to be investigational and expedited reporting of adverse events would follow the guidelines for investigational agents.

- *Sequential administration*: When a study includes an investigational agent(s) and a commercial agent(s) on the same study arm, but the commercial agent(s) is given for a period of time prior to starting the investigational agent(s), expedited reporting of adverse events that occur prior to starting the investigational agent(s) would follow the guidelines for commercial agents. Once therapy with the investigational agent(s) is initiated, all expedited reporting of adverse events should follow the investigational guidelines.

Steps to determine if an adverse event is to be reported in an expedited manner

Step 1: *Identify the type of event using the NCI Common Terminology Criteria for Adverse Events (CTCAE).* The CTCAE provides descriptive terminology and a grading scale for each adverse event listed. A copy of the CTCAE can be downloaded from the CTEP home page (http://ctep.cancer.gov)**.** Additionally, if assistance is needed, the NCI has an Index to the CTCAE that provides help for classifying and locating terms. All appropriate treatment locations should have access to a copy of the CTCAE.

Step 2: *Grade the event using the NCI CTCAE version specified.*

Step 3: *Determine whether the adverse event is related to the protocol therapy (investigational or commercial).* Attribution categories are as follows: Unrelated, Unlikely, Possible, Probable, and Definite.

Step 4: *Determine the prior experience of the adverse event. Expected* events are those that have been previously identified as resulting from administration of the agent. An adverse event is considered *unexpected,* for expedited reporting purposes only, when either the type of event or the severity of the event is not listed in

- the current NCI Agent-Specific Adverse Event List (for treatments using agents provided under an NCI-held IND);
- the drug package insert (for treatments with commercial agents only);
- Section 3.0 of this protocol.

Step 5: *Review Tables 16.1 and 16.2 in the protocol to determine if there are any protocol-specific requirements for expedited reporting of specific adverse events that require special monitoring.*

Step 6: *Determine if the protocol treatment given prior to the adverse event included an investigational agent(s), a commercial agent(s), or a combination of investigational and commercial agents.*

NOTE: If the patient received at least one dose of investigational agent, follow the guidelines in Table 16.1. If no investigational agent was administered, follow the guidelines in Table 16.2.

NOTE: This includes all events that occur within 30 days of the last dose of protocol treatment. Any event that occurs more than 30 days after the last dose of treatment and is attributed possibly, probably, or definitely to the agent(s) must be reported according to the instructions in Table 16.1.

SOUTHWEST ONCOLOGY GROUP
INTAKE CALENDAR

SWOG Patient ID ☐☐☐☐☐☐ **SWOG Study No.** S☐☐☐☐ **Registration Step** ☐

Patient Initials (L, F, M) ____________

Institution/Affiliate ____ Physician ________________________________

Participating Group: Group Name/Study No./Patient ID ______________ / ______________ / ______________

Instructions for the Nurse or Research Coordinator:

Give this intake calendar to the participant for her use with contact information filled in. Instruct the participant to bring her intake calendar to each visit.

This form does not need to be returned to the Data Operations Center.

If you have any questions contact: ________________________________ Telephone: ______________________

Your next appointment is: ________________________________

Instructions for the participant:

This is a monthly calendar on which you are to record the number of tablets you take each day. Be sure you have enough calendars to last until your next appointment. If you develop any side effects from the tablets, mark this on the calendar on the day you note the effect. Bring your calendars with you each time you have an appointment.

Special instructions:

Month: ________________ **Year:** __________

Sunday	Monday	Tuesday	Wednesday	Thursday	Friday	Saturday

19.3 Drugs Known to be Metabolized by Selected CYP450 Isoenzymes

Selected drugs known to be metabolized by CYP3A4

CYP3A4					
**SUBSTRATES**		**INHIBITORS**		**INDUCERS**	
**Generic Name**	**Trade Name**	**Generic Name**	**Trade Name**	**Generic Name**	**Trade Name**
Anti-neoplastics: *e.g.*<br>Docetaxel<br>Gefitinib<br>Irinotecan	 Taxotere<br>Iressa<br>Camptosar	Anti-arrhythmics: *e.g.*<br>Amiodarone<br>Diltiazem<br>Quinidine	 Cordarone, Pacerone<br>Cardizem, Dilacor XR<br>Cardioquin	Aminoglutethimide	Cytadren
Anti-virals: *e.g.*<br>Amprenivir<br>Rifampin	 Agenerase<br>Rifadin	Anti-virals: *e.g.*<br>Amprenavir<br>Indinavir<br>Nelfinavir<br>Ritonavir	 Agenerase<br>Crixivan<br>Viracept<br>Norvir	Antibiotics: *e.g.*<br>Rifabutin<br>Rifampin	 Rifadin<br>Mycobutin
Anxiolytics: *e.g.*<br>Diazepam<br>Sertraline	 Valium<br>Zoloft	Cimetadine	Tagamet	Anticonvulsants: *e.g.*<br>Carbamazapine<br>Phenytoin<br>Pentobarbital<br>Phenobarbital	 Tegretol<br>Dilantin<br>Nembutal<br>Luminal
Cyclosporine	Sandimmune	Cyclosporine	Sandimmune	*Hypericum perforatum* (2)	St. John's Wort
Anti-infectives: *e.g.*<br>Erythromycin<br>Tetracycline	 Erythrocin<br>Sumycin	Antibiotics: *e.g.*<br>Ciprofloxacin<br>Clarithromycin<br>Doxycycline<br>Enoxacin<br>Isoniazid<br>Telithromycin	 Cipro, Ciloxan<br>Biaxin<br>Adoxa, Periostat<br>Penetrex<br>Nydrazid, INH<br>Ketek		
Steroids: *e.g.*<br>Estrogens, conjugated<br>Estradiol<br>Progesterone	 Premarin<br>Climara<br>Crinone	Imatinib	Gleevec		
Haloperidol	Haldol	Haloperidol	Haldol		
Cardiovascular agents: *e.g.*<br>Digitoxin<br>Quinidine	 Crystodigin<br>Cardioquin	Diclofenac	Cataflam, Voltaren		
Anti-hypertensives: *e.g.*<br>Nicardipine<br>Verapamil	 Cardene<br>Calan, Chronovera	Vasodilators: *e.g.*<br>Nicardipine<br>Verapamil	 Cardene<br>Calan, Chronovera		
Anesthetics: *e.g.*<br>Ketamine<br>Lidocaine	 Xylocaine<br>Diprivan	Anesthetics: *e.g.*<br>Lidocaine<br>Propofol	 Xylocaine<br>Diprivan		
Nefazodone	Serzone	Anti-depressants: *e.g.*<br>Nefazodone<br>Sertraline	 Serzone<br>Zoloft		
Cocaine		Anti-fungals: *e.g.*<br>Itraconazole<br>Ketoconazole<br>Miconazole	 Sporanox<br>Nizoral<br>Lotrimin, Monistat		
Ketoconazole	Nizoral	Caffeine			
Sildenafil	Viagra	Grapefruit juice (1)			
Albuterol	Ventolin				
Carbamazapine	Tegretol				
Lovastatin	Mevacor				

When drugs classified as 'substrates' are co-administered with BAY 43-9006 (sorafenib), there is the potential for higher concentrations of the 'substrate'. When BAY 43-9006 (sorafenib) is co-administered with compounds classified as 'inhibitors', increased plasma concentrations of BAY 43-9006 (sorafenib) is the potential outcome. The coadministration of 'inducers' would potentially lower plasma BAY 43-9006 (sorafenib) concentrations.

Comprehensive list of drugs that may have potential interactions with CYP3A4

CYP3A4

Substrates			
Albuterol	Docetaxel	Ketoconazole	Quetiapine
Alfentanil	Doxepin	Lansoprazole	Quinidine
Alprazolam	Doxorubicin	Letrozole	Rabeprazole
Amlodipine	Doxycycline	Levomethadyl acetate hydrochloride	Repaglinide
Amprenavir	Efavirenz		Rifabutin
Aprepitant	Eletriptan	Levonorgestrel	Rifampin
Aripiprazole	Enalapril	Lidocaine	Ritonavir
Atazanavir	Eplerenone	Losartan	Saquinavir
Atorvastatin	Ergoloid mesylates	Lovastatin	Sertraline
Benzphetamine	Ergonovine	Medroxyprogesterone	Sibutramine
Bisoprolol	Ergotamine	Mefloquine	Sildenafil
Bortezomib	Erythromycin	Mestranol	Simvastatin
Bosentan	Escitalopram	Methadone	Sirolimus
Bromazepam	Estradiol	Methylergonovine	Sufentanil
Bromocriptine	Estrogens, conj., synthetic	Methysergide	Tacrolimus
Buprenorphine	Estrogens, conj., equine	Miconazole	Tamoxifen
Buspirone	Estrogens, conj., esterified	Midazolam	Tamsulosin
Busulfan	Estrone	Miglustat	Telithromycin
Carbamazapine	Estropipate	Mirtazapine	Teniposide
Cerivastatin	Ethinyl estradiol	Modafinil	Terbinafine
Chlordiazepoxide	Ethosuximide	Montelukast	Tetracycline
Chloroquine	Etoposide	Moricizine	Theophylline
Chlorpheniramine	Felbamate	Nateglinide	Tiagabine
Cisapride	Felodipine	Nefazodone	Ticlopidine
Citalopram	Fentanyl	Nelfinavir	Tolterodine
Clarithromycin	Flurazepam	Nevirapine	Toremifene
Clobazam	Flutamide	Nicardipine	Trazodone
Clonazepam	Fosamprenavir	Nifedipine	Triazolam
Clorazepate	Fulvestrant	Nimodipine	Trimethoprim
Cocaine	Gefitinib	Nisoldipine	Trimipramine
Colchicine	Halofantrine	Nitrendipine	Troleandomycin
Cyclophosphamide	Haloperidol	Norethindrone	Vardenafil
Cyclosporine	Ifosfamide	Norgestrel	Venlafaxine
Dantrolene	Imatinib	Ondansetron	Verapamil
Dapsone	Indinavir	Paclitaxel	Vinblastine
Delavirdine	Irinotecan	Pergolide	Vincristine
Diazepam	Isosorbide dinitrate	Phencyclidine	Vinorelbine
Digitoxin	Isosorbide mononitrate	Pimozide	Zolpidem
Dihydroergotamine	Isradipine	Pioglitazone	Zonisamide
Diltiazem	Itraconazole	Primaquine	Zopiclone
Disopyramide	Ketamine	Progesterone	

CYP3A4

Inhibitors			
Acetominophen	Diltiazem	Lovastatin	Progesterone
Acetazolamide	Disulfiram	Mefloquine	Propofol
Amioderone	Docetaxel	Mestranol	Propoxyphene
Amlodipine	Doxorubicin	Methadone	Quinidine
Amprenavir	Doxycycline	Methimazole	Quinine
Anastrozole	Drospirenone	Methoxsalen	Quinupristin
Aprepitant	Efavirenz	Methylprednisolone	Rabeprazole
Atazanavir	Enoxacin	Metronidazole	Risperidone
Atorvastatin	Entacapone	Miconazole	Ritonavir
Azelastine	Ergotamine	Midazolam	Saquinavir
Azithromycin	Erythromycin	Mifepristone	Selegiline
Betamethasone	Ethinyl estradiol	Mirtazapine	Sertraline
Bortezomib	Etoposide	Mitoxantrone	Sildenafil
Bromocriptine	Felodipine	Modafinil	Sirolimus
Caffiene	Fentanyl	Nefazodone	Sulconazole
Cerivastatin	Fluconazole	Nelfinavir	Tacrolimus
Chloramphenicol	Fluoxetine	Nevirapine	Tamoxifen
Chlorzoxazone	Fluvastatin	Nicardipine	Telithromycin
Cimetadine	Fluvoxamine	Nifedipine	Teniposide
Ciprofloxacin	Fosamprenavir	Nisoldipine	Testosterone
Cisapride	Glyburide	Nitrendipine	Tetracycline
Clarithromycin	Grapefruit juice	Nizatidine	Ticlopidine
Clemastine	Haloperidol	Norfloxacin	Tranylcypromine
Clofazimine	Hydralazine	Olanzapine	Trazodone
Clotrimazole	Ifosfamide	Omeprazole	Troleandomycin
Clozapine	Imatinib	Orphenadrine	Valproic acid
Cocaine	Indinavir	Oxybutynin	Venlafaxine
Cyclophosphamide	Irbesartan	Paroxetine	Verapimil
Cyclosporine	Isoniazid	Pentamidine	Vinblastine
Danazol	Isradapine	Pergolide	Vincristine
Delavirdine	Itraconazole	Phencyclidine	Vinorelbine
Desipramine	Ketoconazole	Pilocarpine	Zafirlukast
Dexmedetomidine	Lansoprazole	Pimozide	Ziprasidone
Diazepam	Lidocaine	Pravastatin	
Diclofenac	Lomustine	Prednisolone	
Dihydroergotamine	Losartan	Primaquine	

Inducers			
Aminoglutethimide	Nevirapine	Phenytoin	Rifapentine
Carbamazapine	Oxcarbazepine	Primidone	
Fosphenytoin	Pentobarbital	Rifabutin	
St. John's wort	Phenobarbital	Rifampin	

(Adapted from Cytochrome P-450 Enzymes and Drug metabolism. In: Lacy CF, Armstrong LL, Goldman MP, Lance LL eds. Drug Information Handbook 12TH ed. Hudson, OH; LexiComp Inc. 2004: 1619-1631.)

(1) Malhorta *et al*. (2000). Clin Pharmacol Ther. 69:14-23

(2) Mathijssen *et al*. (2002). J Natl Cancer Inst. 94:1247-1249
Frye *et al*. (2004). Clin Pharmacol Ther. 76:323-329

19.4 CARBOPLATIN DOSING WORKSHEET*

PATIENT'S INITIALS ____________________ **MEDICAL RECORD #** ____________________________

SWOG PATIENT # ______________________________

TO CALCULATE Creatinine Clearance (CrCl) from SERUM CREATININE:

CrCl = $\dfrac{\text{(140 - age) X wt. in kg.**}}{\text{72 X serum creatinine}}$ **X 0.85 (female) OR X 1.00 (male)**

CrCl = $\dfrac{\text{(140 - \quad) X (\quad)}}{\text{72 X \_\_\_\_\_\_\_\_\_\_\_\_\_\_\_\_\_\_\_\_}}$ **X 0.85 (female) OR X 1.00 (male)**

TO CALCULATE CARBOPLATIN DOSE WITH CALVERT FORMULA:

***USE CALCULATED CREATININE CLEARANCE (AS ABOVE) TO SUBSTITUTE FOR GFR**

(6)(*GFR + 25) = CARBOPLATIN DOSE PER CYCLE IN mg

(6)(________+ 25) = ____________________ mg of carboplatin

This is the TOTAL DOSE of carboplatin (not mg/m^2)

*** Carboplatin dose should be calculated using a serum creatinine value obtained within 3 days prior to each course therapy.**

****Use current (actual) weight.**

PLEASE RETAIN ORIGINAL WORK SHEET as part of patient's PERMANENT RECORD.

COPIES MAY BE MADE FOR PHARMACY and/or NURSING RECORDS.
